# Photoinduced C–H arylation of 1,3-azoles *via* copper/photoredox dual catalysis†

**DOI:** 10.1039/d4sc00393d

**Published:** 2024-04-16

**Authors:** Sven Trienes, Jiawei Xu, Lutz Ackermann

**Affiliations:** a Institut für Organische und Biomolekulare Chemie, Wöhler Research Institute for Sustainable Chemistry (WISCh), Georg-August-Universität Tammannstraße 2 37077 Göttingen Germany lutz.ackermann@chemie.uni-goettingen.de; b DZHK (German Centre for Cardiovascular Research) Potsdamer Straße 58 10875 Berlin Germany

## Abstract

The visible light-induced C–H arylation of azoles has been accomplished by dual-catalytic system with the aid of an inexpensive ligand-free copper(i)-catalyst in combination with a suitable photoredox catalyst. An organic photoredox catalyst, 10-phenylphenothiazine (PTH), was identified as effective, cost-efficient and environmentally-benign alternative to commonly-used, expensive Ir(iii)-based complexes. The method proved applicable for the C–H arylation of various azole derivatives, including oxazoles, benzoxazoles, thiazoles, benzothiazoles as well as more challenging imidazoles and benzimidazoles. Moreover, the derivatization of complex molecules and the gram scale synthesis of the natural product balsoxin reflected the synthetic utility of the developed strategy. Mechanistic studies were indicative of a single electron transfer-based (SET) mechanism with an aryl radical as key intermediate.

## Abstract 2

The photo-induced azole C–H arylation was realized with a dual-catalytic copper-based system. Exceedingly mild reaction conditions at ambient temperature and visible light irradiation enabled a broad scope with high functional group tolerance.

## Graphical abstract

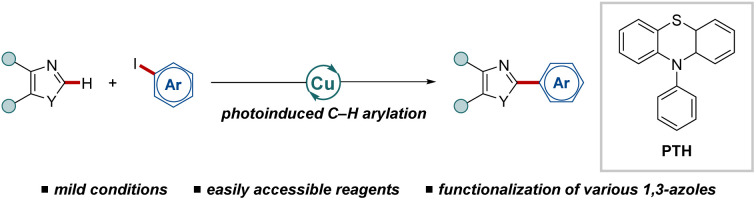

## Introduction

The direct functionalization of C–H bonds represents a highly efficient, powerful, and sustainable transformation in modern organic synthesis outperforming traditional cross-coupling reactions with regards to step- and atom-economy.^1–6^ Utilizing the C–H activation approach, valuable and important molecular scaffolds with broad applications ranging from material sciences^7,8^ to drug discovery^9–11^ and crop protection can be elegantly accessed.^12,13^

**Scheme 1:**
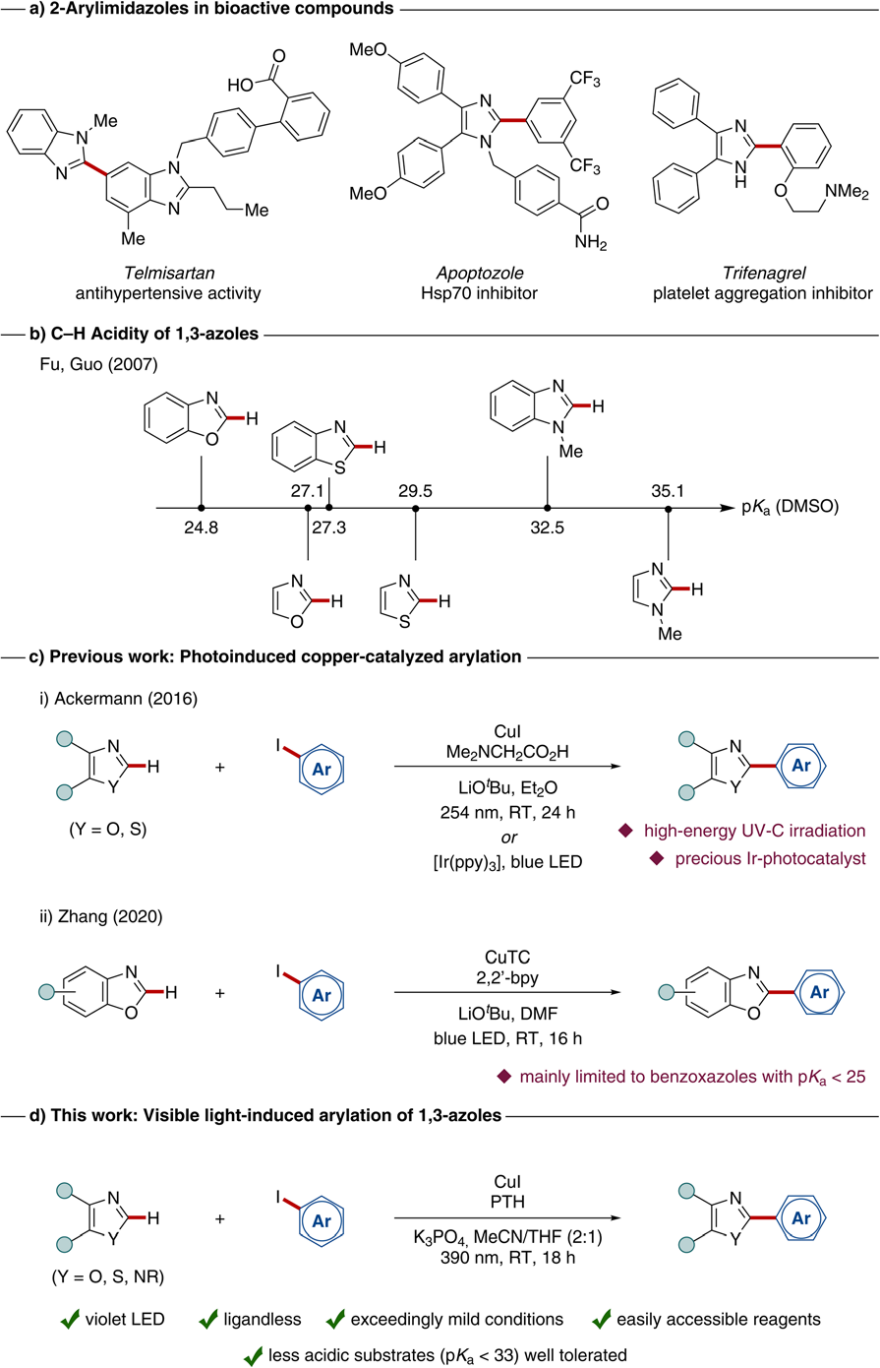
(a) 2-Aryl imidazole motifs in pharmaceuticals. (b) Predicted p*K*_a_-values for 1,3-azoles in DMSO.^26^ Copper-catalyzed C–H arylations of azoles *via* (c) photo-induced copper-catalyzed procedures for the arylation of oxazoles and thiazoles and (d) our approach for arylations under mild visible light irradiation.

As 2-aryl benzimidazoles and imidazoles often exhibit interesting bioactive properties,^14–21^ these structural motifs are therefore important building blocks in pharmacologically relevant molecules (Scheme 1a). Classical methods for their synthesis comprise the *de novo* synthesis of the heterocyclic core *via* condensation–cyclization reactions. Most of these strategies imply multi-step syntheses of starting materials and require stoichiometric amounts of toxic reagents, strong acids or harsh reaction conditions.^22–25^ Due to the high relevance of these scaffolds, a sustainable, mild, and efficient synthesis is highly desirable, as represented by the direct C–H arylation of the corresponding core heterocycles. In this context, the C–H acidity of the targeted heterocycle is an important indicator for the reactivity of a certain C–H bond (Scheme 1b).^26^

Although the application of 4d^27–31^ and 5d^32–36^ transition metals has been well-demonstrated for C–H activation strategies, their high price and rather toxic properties limit real application. During the last years, remarkable progress could be observed in the field of first row transition-metal catalyzed C–H functionalizations, thus highlighting earth-abundant and environmentally-benign 3d metals as powerful substitute for their heavier analogues.^37,38^ Especially, copper(i) salts proved to be a powerful catalyst in the direct functionalization of azole-based compounds, with major contributions by Daugulis,^39–41^ Miura,^42^ and Ackermann,^43,44^ among others.^45–47^ However, these methods typically require high reaction temperatures ranging from 100–160 °C.

In sharp contrast, the merger of photocatalysis^48–55^ with copper-catalyzed transformations allows to perform organic transformations under significantly milder conditions.^56^ In this context, our group reported in 2016 on a photoinduced C–H functionalization of benzoxazoles and benzothiazoles (Scheme 1c(i)) using UV-C irradiation.^57^ Interestingly, copper(i)-complexes were employed as dual-functional catalysts, which mediate the C–C bond formation and also serve as the photocatalyst. Additionally, a first example for the arylation using visible light in combination with an iridium-photocatalyst was reported in the same work.

More recent studies focused on the use of visible light, as demonstrated in cross-coupling reactions.^58^ In 2020, Zhang employed a photoactive copper(i) complex as sole catalyst for C–H arylations of benzoxazoles, benzothiazoles and electron-deficient thiophenes (Scheme 1c(ii)).^59^ However, known strategies for the photoinduced arylation of azoles are mainly limited to benzoxazoles and benzothiazoles with a p*K*_a_ value below 27.5.^26^ In contrast, only few precedents are known for the direct arylation of more challenging benzimidazoles (p*K*_a_ = 32.5) under photochemical conditions. In this context, our group has enabled the direct arylation utilizing an immobilized copper(i) catalyst,^60^ however, UV-C irradiation is typically required.

Within our program on photoinduced C–H functionalizations,^61–65^ we have now developed a strategy for the direct arylation of benzimidazoles at room temperature enabled by visible light irradiation (Scheme 1d). Key features of our method include (i) a dual catalytic, ligand-free system with a cost-efficient copper(i) precursor and an exogeneous photoredox catalyst which allows for (ii) visible light-induced transformations under (iii) mild conditions at ambient temperature with (iv) broad applicability to various azole-based compounds.

## Results and discussion

### Optimization of the reaction conditions

We commenced our studies by probing various reaction conditions to enable the envisioned copper-catalyzed arylation of benzimidazole 1a with aryl iodide 2a (Table 1) under blue LED (450 nm) irradiation. The desired arylation product 3a was obtained with 71% yield using CuI as copper source in combination with [Ir(*p*-F-ppy)_3_] as photoredox catalyst and K_3_PO_4_ as base in a solvent mixture of MeCN and THF (2 : 1) (entry 1). THF or MeCN as sole solvents furnished product 3a in significantly reduced yields of 38% or 7%, respectively (entries 2–3). Other bases, such as K_2_CO_3,_ led to a diminished yield, while LiO^*t*^Bu failed in the transformation (entries 4–5). Alternative copper(i) sources, such as CuTC (copper(i) thiophene-2-carboxylate) or CuOAc instead of CuI caused a slightly decreased efficacy in the arylation (entries 6–7). In contrast to [Ir(*p*-F-ppy)_3_] photoredox catalyst, the commonly used *fac*-[Ir(ppy)_3_] photoredox catalyst furnished product 3a in an unsatisfactory yield of 20% (entry 8). In order to circumvent the use of rare and expensive iridium(iii)-based catalysts and to reduce undesired, potential trace metal impurities in the product, we thereafter probed organic photoredox catalysts (entries 9–10). Acridinium salt [Mes-Acr]BF_4_ failed to enable the reaction (entry 9). In contrast, 10-phenylphenothiazine (PTH)^66,67^ exhibited notable efficacy as photoredox catalyst using violet LED irradiation with a wavelength of 390 nm (entry 10). The modified conditions furnished product 3a in 77% yield. 4-Bromotoluene (entry 11) proved viable as electrophile, albeit with lower catalytic efficacy.^68^ Control experiments confirmed the essential role of the copper(i) salt and the photochemical nature of the reaction (entry 12). Furthermore, the essential role and positive synergistic effects of the addition of a photoredox catalyst were highlighted in further control experiments (entries 13–14).^69^

**Table 1:** Optimization of reaction conditions

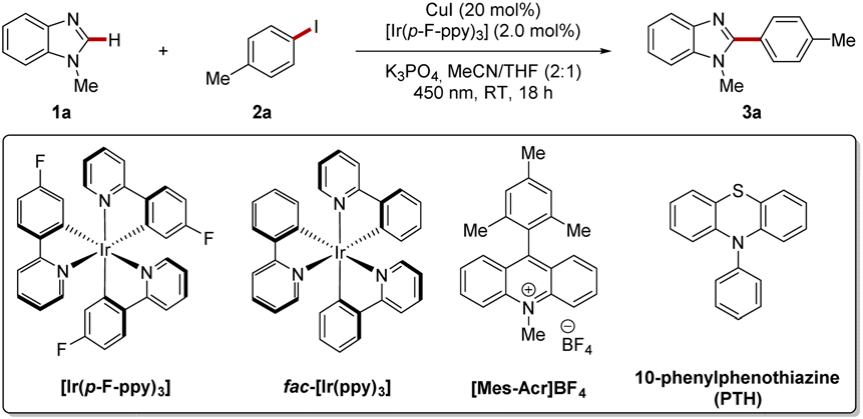		
Entry	Deviation from standard conditions	Yield^a^ (%)
1	None	71
2	THF instead of MeCN/THF (2 : 1)	38
3	MeCN instead of MeCN/THF (2 : 1)	7^b^
4	K_2_CO_3_ instead of K_3_PO_4_	58
5	LiO^*t*^Bu instead of K_3_PO_4_	0^b^
6	CuTC instead of CuI	62
7	CuOAc instead of CuI	63
8	*fac*-[Ir(ppy)_3_] instead of [Ir(*p*-F-ppy)_3_]	20
9	[Mes-Acr]BF_4_ instead of [Ir(*p*-F-ppy)_3_]	0^b^^,^^c^
10	PTH instead of [Ir(*p*-F-ppy)_3_] at 390 nm	77^d^
11	4-Br-Tol instead of 2a and PTH instead of [Ir(*p*-F-ppy)_3_] at 390 nm	45^b^
12	No CuI or no light	0^b^
13	Without photocatalyst at 450 nm	0^b^
14	Without photocatalyst at 390 nm	44

a Reaction conditions: 1a (0.25 mmol), 2a (0.75 mmol), CuI (20 mol%), [Ir(*p*-F-ppy)_3_] (2.0 mol%), K_3_PO_4_ (0.75 mmol), MeCN/THF (2 : 1) (1 mL), 35 °C, 18 h, under N_2_, blue LEDs (450 nm); yield of isolated products.

b The yield was determined by ^1^H-NMR spectroscopy using 1,3,5-trimethoxybenzene as internal standard.

c 10 mol% [Mes-Acr]BF_4_.

d 5.0 mol% PTH. Mes-Acr = 9-mesity-10-methylacridinium. PTH = 10-phenylphenothiazine.

### Photocatalysis robustness

**Scheme 2:**
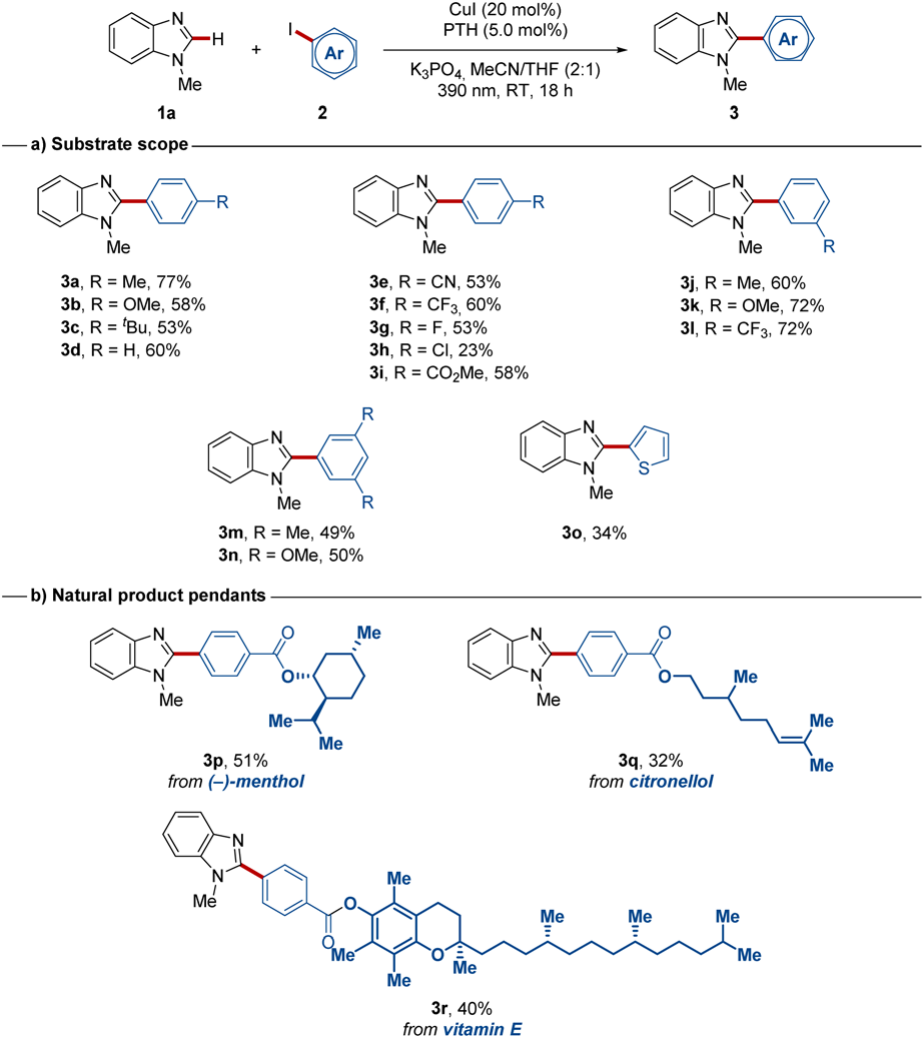
Substrate scope for aryl iodides in photo-induced copper(i)-catalyzed C–H arylations at room temperature.

With the optimized conditions in hand, we probed the versatility of the photo-induced copper-catalyzed C–H arylation with PTH for a set of differently substituted aryl iodides 2 (Scheme 2a). Both, electron-donating as well as electron-withdrawing substituents in various positions proved viable (3a–3o) with yields up to 77%. The mild photocatalysis also tolerated aryl iodides with sensitive functionalities, such as synthetically useful nitrile (3e) and ester (3i) substituents, which were transformed in a chemoselective manner. Also, the chloro-containing product 3h could be isolated, albeit with a reduced yield due to partial protodehalogenation. Finally, 2-iodothiophene (3o) and natural product-embedded aryl iodides (3p–3r) proved to be suitable substrates setting the stage for more complex molecule diversifications through the developed method (Scheme 2b).

**Scheme 3:**
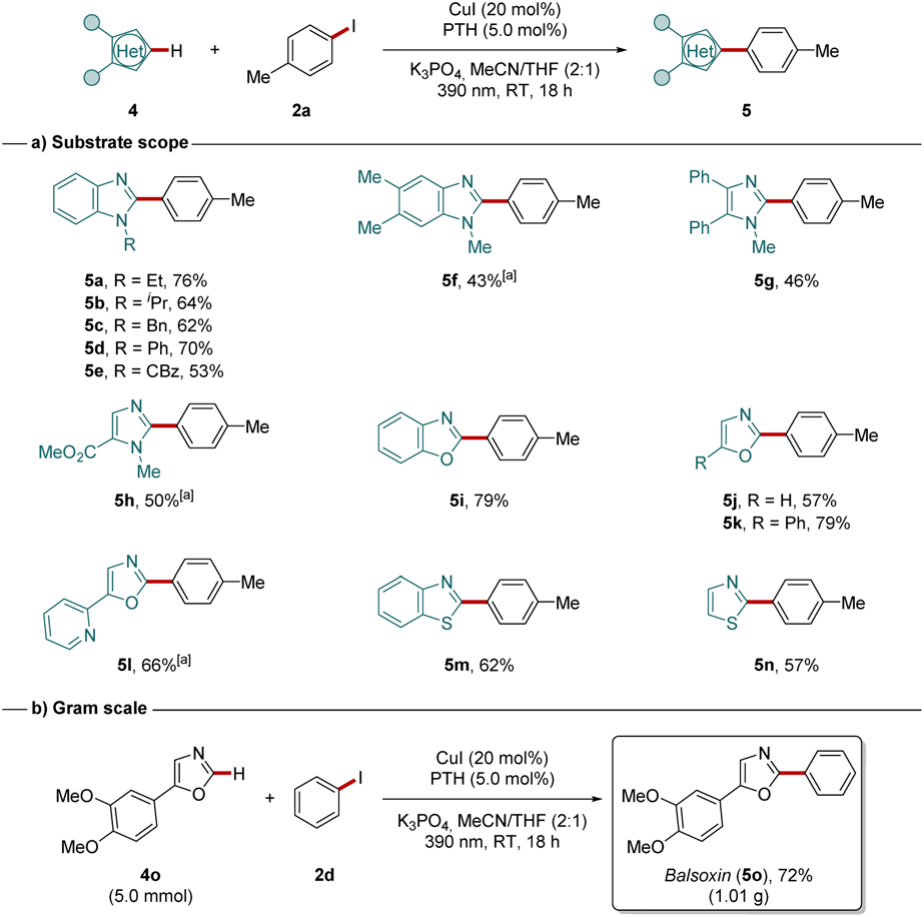
Substrate scope for differently substituted azole. [a] 4 (0.25 mmol), 2a (1.00 mmol), [Cu(MeCN)_4_]PF_6_ (30 mol%), PTH (5.0 mol%), K_3_PO_4_ (0.75 mmol), MeCN/THF (2 : 1, 0.5 mL), 390 nm, RT, 48 h.

Moreover, the photo-induced copper-catalyzed C–H arylation was also suitable for differently substituted benzimidazoles (5a–5f) (Scheme 3a). Additionally, 4,5- and 4-substituted imidazoles (5g, 5h), as well as other heterocyclic compounds with more acidic C–H bonds like benzoxazole (5i), oxazoles (5j–5l), benzothiazole (5m) and thiazole (5n) have been efficiently converted in the photoinduced arylation reaction. The scalability and synthetic utility of our approach were further demonstrated by the gram scale synthesis of the natural product balsoxin (5o), which was isolated in 72% yield (Scheme 3b).

**Scheme 4:**
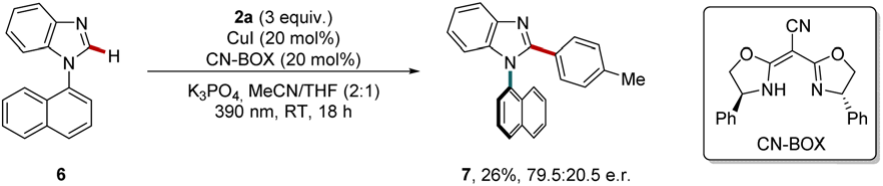
Initial results on the photo-induced atroposelective C–H arylation of benzimidazoles.

To further advance the developed methodology, we aimed to realize a concept for the arylation of benzimidazoles in an enantioselective fashion. At the outset of our studies, we identified that the introduction of a sterically demanding substituent at the N(1) position, as represented by naphthyl-substituted benzimidazole 6, resulted in an atropostable C–N axis through C–H arylation. Interestingly, the combination of CuI with the anionic cyano-bisoxazoline ligand (CN-BOX) achieved promising results after initial optimizations,^68^ furnishing 26% of the atropostable product 7 with 79.5 : 20.5 e.r. (Scheme 4).

### Mechanistic considerations

**Scheme 5:**
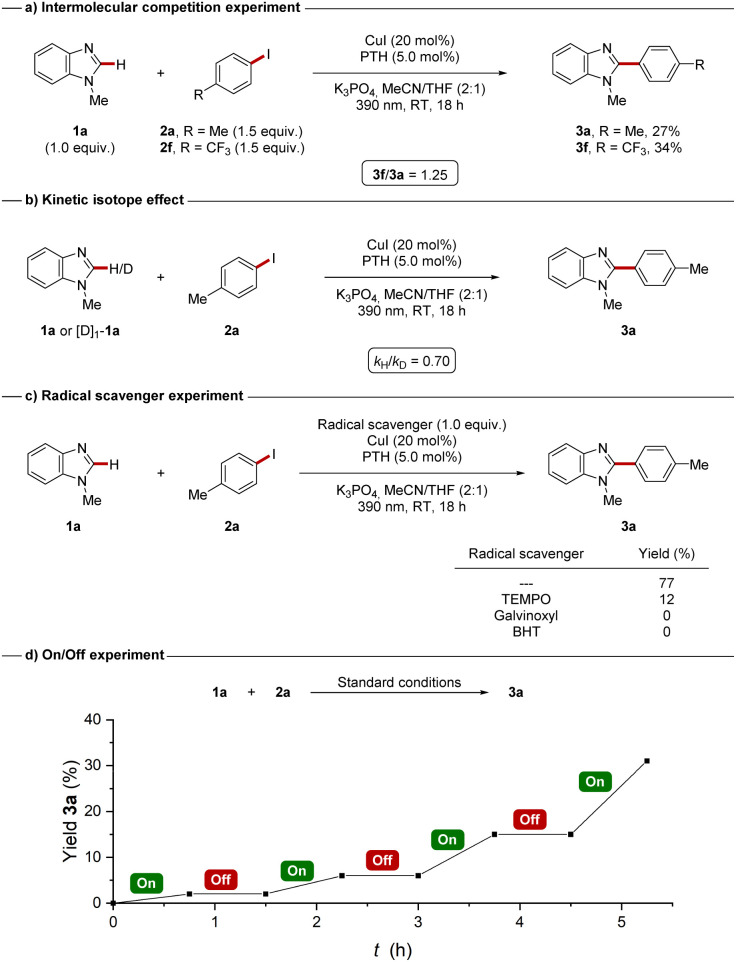
Key mechanistic experiments.

Next, we investigated the mode of action of the dual-catalytic C–H arylation by carrying out a set of mechanistic experiments. An intermolecular competition experiment between 4-iodotoluene (2a) and 4-iodobenzotrifluoride (2f) revealed an inherently higher reactivity of the electron-deficient 4-iodobenzotrifluoride (Scheme 5a). These findings could suggest a single-electron reduction of the aryl iodide.^70,71^ A kinetic isotope effect experiment revealed a rather unusual *k*_H_/*k*_D_ of 0.70 (Scheme 5b) which could indicate a change in hybridization of the C(2) of benzimidazole 1a. To probe, whether a SET-type regime is operative, radical scavenger experiments (Scheme 5c) were conducted. The presence of TEMPO (2,2,6,6-tetramethylpiperidinyloxyl) caused a significant inhibition of the arylation, while galvinoxyl or BHT (butylated hydroxytoluene) completely suppressed the reaction, thereby suggesting a radical mechanism. Moreover, the formation of the TEMPO- and galvinoxyl-tolyl adducts were detected *via* HRMS analysis, which further supported a single-electron reduction pathway generating an aryl radical species.^68^ The role of the violet LED irradiation was further elucidated by an on/off experiment. As the reaction completely ceased in the absence of light (Scheme 5d), a radical chain pathway seemed unlikely for the reaction. Additionally, a low quantum yield substantiated the absence of a radical chain mechanism.^68^ Fluorescence quenching studies at 390 nm furthermore showed an effective quenching of the excited state of the PTH photocatalyst by aryl iodide 2a indicating an one-electron oxidative quenching pathway.^68^

**Scheme 6:**
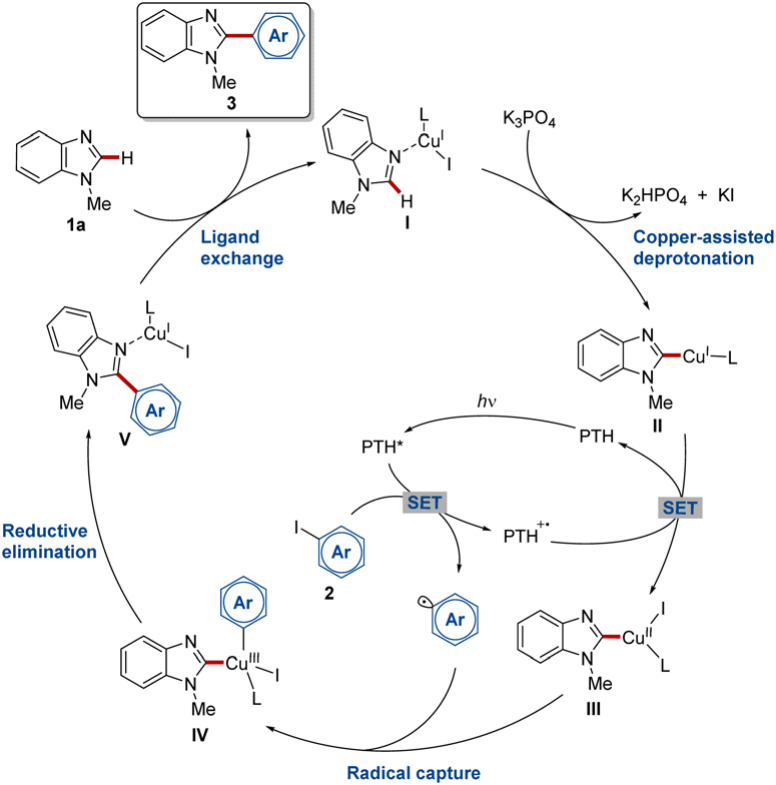
Proposed catalytic cycle (L = MeCN, THF, 1a).

Based on literature precedents and our mechanistic findings, a plausible mechanistic scenario for the photo-induced copper-catalyzed C–H arylation was proposed (Scheme 6). Starting with copper complex I which is formed *in situ* from copper(i) iodide through coordination of the substrate and solvent molecules. The coordination of copper at the Lewis-basic N(3) nitrogen of 1a causes an increased C–H acidity facilitating the copper-assisted C–H bond cleavage in C(2) position forming copper complex II.^72^ Oxidative quenching of the excited PTH* species through a SET process generates an aryl radical, which is captured by copper complex III formed by the oxidation of complex II and thus regenerating the photoredox catalyst. The resulting copper(iii) complex IV undergoes reductive elimination thereby forming the desired C–C bond. After ligand exchange with another benzimidazole molecule 1a, the product is released and, at the same time, the catalyst is regenerated.

## Conclusions

In conclusion, we introduced a versatile synergistic, copper-catalyzed C–H arylation which enables the efficient functionalization of a variety of azoles at ambient temperature. Notably, the C–H functionalization has not only been successfully performed on oxazoles and thiazoles, but also on more challenging azole-based compounds, including benzimidazoles. Exceedingly mild conditions enabled an ample scope. Additionally, the use of the readily available, inexpensive organic PTH as photoredox catalyst proved to be a powerful alternative to high-priced iridium-photocatalysts, thereby improving the cost-efficiency and the environmental footprint of our method.

## Data availability

The data supporting this article have been uploaded as part of the ESI.†

## Author contributions

Conceptualization, L. A.; funding acquisition, L. A.; investigation S. T. and J. X.; methodology, S. T. and J. X.; resources, L. A.; supervision, L. A.; writing – original draft, L. A. and S. T.; writing – reviewing & editing, L. A., S. T. and J. X.

## Conflicts of interest

There are no conflicts to declare.

## Supplementary Material

SC-015-D4SC00393D-s001
