# Supplementary material for: Photoinduced C–H arylation of 1,3-azoles via copper/photoredox dual catalysis
Source: Chem Sci. 2024 Apr 16;15(19):7293–9. doi: 10.1039/d4sc00393d (PMC11095366; doi:10.1039/d4sc00393d)

Electronic Supplementary Material (ESI) for Chemical Science.
This journal is © The Royal Society of Chemistry 2024

Supporting Information

Photoinduced C–H arylation of 1,3-azoles via copper/photoredox dual catalysis

Sven Trienes,[a,b] *Jiawei Xu,*[a] *and Lutz Ackermann*[a,b]*

[a] Institut für Organische und Biomolekulare Chemie and Wöhler Research Institute for Sustainable Chemistry (WISCh), Georg-August-Universität, Tammannstraße 2, 37077 Göttingen (Germany)

[b] DZHK (German Centre for Cardiovascular Research), Potsdamer Straße 58, 10875 Berlin (Germany)

Email: lutz.ackermann@chemie.uni-goettingen.de

http://www.ackermann.chemie.uni-goettingen.de

TABLE OF CONTENTS: **Page**

**1**	**General remarks**	**3**
**2**	**Synthesis of starting materials**	**4**
	2.1 Synthesis of 1,3-azoles	4
	2.2 Synthesis of aryl iodides	6
**4**	**Optimization studies**	**9**
**5**	**Light-induced copper-catalyzed C–H arylation**	**13**
**6**	**Characterization data**	**15**
**7**	**Gram scale synthesis**	**34**
**8**	**Synthesis of enantioenriched benzimidazole 7**	**35**
**9**	**Mechanistic experiments**	**37**
	9.1 Intermolecular competition experiment	37
	9.2 Kinetic isotope effect	38
	9.3 Radical scavenger experiments	40
	9.4 On/off experiment	43
	9.5 Determination of quantum yield	44
	9.6 Fluorescence quenching experiment	46
**10**	**References**	**48**
**11**	**NMR Spectra**	**50**

1 General remarks

General conditions: Catalytic reactions were performed under a N_2 atmosphere using pre-dried glassware and standard Schlenk techniques. THF and MeCN were purified by a MBraun MB SPS-800 solvent purification system, degassed by multiple cycles of freeze-pump-thaw method and dried over 4 Å molecular sieves. All substrates and reagents were obtained from commercial sources and were used without further purification except those mentioned in the following section.

Chromatographic methods: TLC was performed on Merck TLC Silica Gel 60 F_{254} with detection under UV light at 254 nm. Chromatographic separations were carried out on Merck Geduran SI-60 (40–63 µm, 230–400 mesh ASTM). GC-MS chromatograms were recorded on Agilent 7890B and Agilent 5977B. HPLC chromatograms were recorded on an Agilent 1290 Infinity using CHIRALPAK® IA-3 column (3.0 µm particle size; Ø: 4.6 mm and 250 mm length).

IR Spectroscopy: IR spectra were recorded on a Bruker FT-IR alpha-P device.

Mass spectrometry: ESI-MS was recorded on Bruker Daltonik micrOTOF and maXis. The ratios of mass to charge (*m*/*z*) are reported and the intensity relative to the base peak (I = 100) is given in parentheses.

Melting point: Melting points (m.p.) were measured on Stuart® melting point apparatus SMP3, values are uncorrected.

Spectroscopic methods: Nuclear magnetic resonance (NMR) spectroscopy was performed at 300 MHz, 400 MHz, 500 MHz (^{1}H NMR), 101 MHz, 126 MHz (^{13}C NMR, APT), 377 MHz (^{19}F NMR) on Bruker Avance III HD 300, Avance Neo 400, and Avance III HD 500 instruments. Chemical shifts (δ) are provided in ppm and spectra refered to solvent signal ($CDCl_3$: 7.26 ppm (^{1}H NMR); 77.16 ppm (^{13}C NMR)). Fluorescene spectra were measured on a Jasco spectrofluorometer FP-8500.

2 Synthesis of starting materials

All compounds except those shown in the following scheme were obtained from commercial sources. 10-Phenylphenothiazine (PTH) was synthesized through a procedure reported by Zhao and Guo.[1] Before use, PTH was further purified by Kugelrohr distillation. The shown compounds were synthesized according to literature procedures.

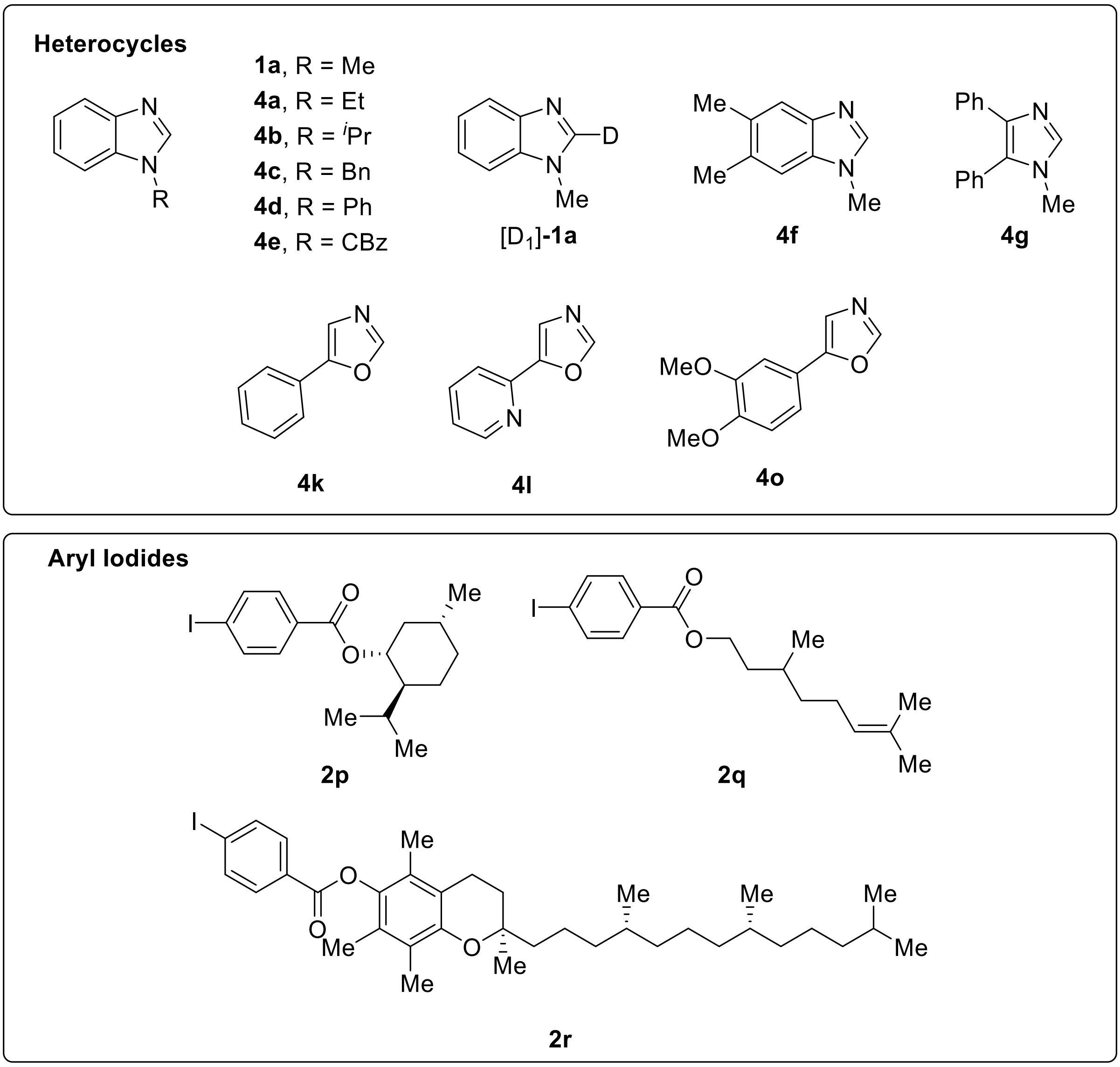

2.1 Synthesis of 1,3-azoles

The following compounds are known. Analytical data match with those reported in the literature.

1-Methyl-1*H*-benzo[*d*]imidazole (1a): Synthesized according to Breit with analytical data matching with reported data.[2]

1-Ethyl-1*H*-benzo[*d*]imidazole (4a): Synthesized according to Rit with analytical data matching with reported data.[3]

1-Isopropyl-1*H*-benzo[*d*]imidazole (4b): Synthesized according to Yagi with analytical data matching with reported data.[4]

1-Benzyl-1*H*-benzo[*d*]imidazole (4c): Synthesized according to Xia with analytical data matching with reported data.[5]

1-Phenyl-1*H*-benzo[*d*]imidazole (4d): Synthesized according to Dong with analytical data matching with reported data.[6]

1-Methyl-1*H*-benzo[*d*]imidazole-2-*d* ([D$_1$]-1a): Synthesized according to Ong with analytical data matching with reported data.[7]

1,5,6-Trimethyl-1*H*-benzo[*d*]imidazole (4f): Synthesized according to Breit.[2] Analytical data match with reported data.[4]

1-Methyl-4,5-diphenyl-1*H*-imidazole (4g): Synthesized according to Breit.[2] Analytical data match with reported data.[8]

5-Phenyloxazole (4k): Synthesized according to Piguel with analytical data matching with reported data.[9]

5-(Pyridin-2-yl)oxazole (4l): Synthesized according to Piguel.[9] Analytical data match with reported data.[10]

5-(3,4-Dimethoxyphenyl)oxazole (4o): Synthesized according to Piguel with analytical data matching with reported data.[9]

(1*R*,2*S*,5*R*)-2-Isopropyl-5-methylcyclohexyl 4-iodobenzoate (2p): Synthesized according Reiser with analytical data matching with reported data.[11]

Synthesis of benzyl 1*H*-benzo[*d*]imidazole-1-carboxylate (4e)

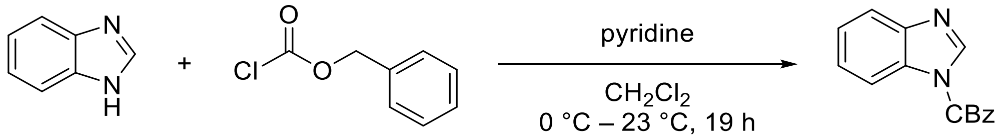

The synthesis follows a modified procedure described by Kwon.[12]

A round-bottom Schlenk flask was charged with benzimidazole (10 mmol, 1.0 equiv.), CH_2Cl_2 (25 mL) and pyridine (20 mmol, 2.0 equiv.). At 0 °C, benzyl chloroformate (15 mmol, 1.5 equiv.) was added slowly. The resulting solution was stirred at 0 °C for 1 h and for 18 h at ambient temperature. Afterwards, all volatiles were removed under reduced pressure and the

residue was purified by column chromatography on silica gel (CH_2Cl_2/EtOAc 1:1) to obtain the desired product as colorless solid (2.27 g, 90%).

M.p.: 74 °C.

^{1}H NMR (400 MHz, $CDCl_3$): δ = 8.48 (s, 1H), 8.04 – 8.00 (m, 1H), 7.82 – 7.77 (m, 1H), 7.50 (dd, J = 7.8, 1.9 Hz, 2H), 7.47 – 7.33 (m, 5H), 5.51 (s, 2H) ppm.

^{13}C NMR (101 MHz, $CDCl_3$): δ = 149.5 (C_q), 144.1 (C_q), 141.8 (CH), 134.3 (C_q), 131.4 (C_q), 129.3 (CH), 129.1 (CH), 128.9 (CH), 125.7 (CH), 124.7 (CH), 120.9 (CH), 114.5 (CH), 69.8 (CH_2).

IR (ATR): $\tilde{\nu}$ = 1745, 1451, 1389, 1359, 1343, 1285, 1235, 1054, 744, 697 cm^{-1}.

MS (ESI) *m/z* (relative intensity): 527 (60) $[2M+Na]^+$, 275 (100) $[M+Na]^+$.

HRMS (ESI): *m/z* calcd. for $C_{15}H_{12}N_2NaO_2^+$ $[M+Na]^+$ 275.0791, found 275.0796.

2.2 Synthesis of aryl iodides

General Procedure A:

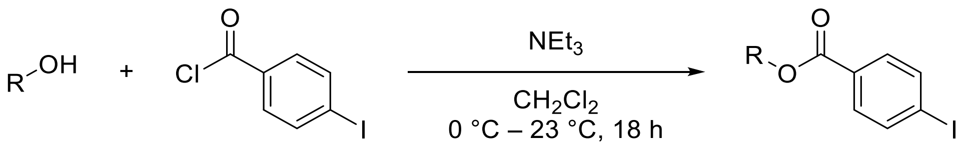

In a round-bottom Schlenk flask, the corresponding alcohol (5.0 mmol, 1.0 equiv.) and NEt_3 (6.0 mmol, 1.2 equiv.) were dissolved in CH_2Cl_2 (40 mL). At 0 °C, 4-iodobenzoyl chloride (6.0 mmol, 1.1 equiv.) was added. The resulting solution was stirred at ambient temperature for 18 h. Afterwards, the reaction was quenched by the addition of water (40 mL). The aqueous phase was extracted with CH_2Cl_2 (3 x 50 mL). Combined organic phases were dried over Na_2SO_4. After filtration and removal of the solvent under reduced pressure, the residue was purified by column chromatography on silica gel to give the corresponding aryl iodide.

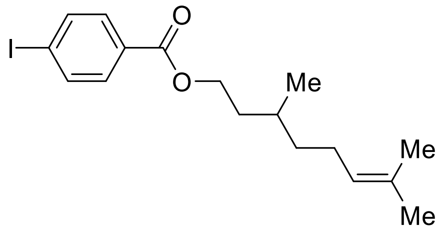

3,7-Dimethyloct-6-en-1-yl 4-iodobenzoate (2q)

Following general procedure A, citronellol was used for the synthesis of compound **2r** which was obtained after column chromatography (hexane/EtOAc 25:1) as colorless oil (1.72 g, 89%).

^{1}H NMR (400 MHz, $CDCl_3$): δ = 7.79 (d, J = 8.5 Hz, 2H), 7.73 (d, J = 8.5 Hz, 2H), 5.09 (tt, J = 7.0, 1.4 Hz, 1H), 4.41 – 4.28 (m, 2H), 2.09 – 1.90 (m, 2H), 1.88 – 1.74 (m, 1H), 1.71 – 1.51 (m, 8H), 1.46 – 1.32 (m, 1H), 1.23 (dddd, J = 13.6, 9.3, 7.5, 6.2 Hz, 1H), 0.96 (d, J = 6.5 Hz, 3H) ppm.

^{13}C NMR (101 MHz, $CDCl_3$): δ = 166.3 (C_q), 137.8 (CH), 131.6 (C_q), 131.1 (CH), 130.1 (C_q), 124.6 (CH), 100.7 (C_q), 63.9 (CH_2), 37.1 (CH_2), 35.6 (CH_2), 29.7 (CH_3), 25.9 (CH_3), 25.5 (CH_2), 19.6 (CH_3), 17.8 (CH) ppm.

IR (ATR): $\tilde{\nu}$ = 2908, 1719, 1586, 1393, 1264, 1176, 1099, 1007, 845, 752 cm^{-1}.
MS (ESI) m/z (relative intensity): 795 (25) $[2M+Na]^+$, 409 (100) $[M+Na]^+$.
HRMS (ESI): m/z calcd. for $C_{17}H_{23}O_2INa^+$ $[M+Na]^+$ 409.0635, found 409.0635.

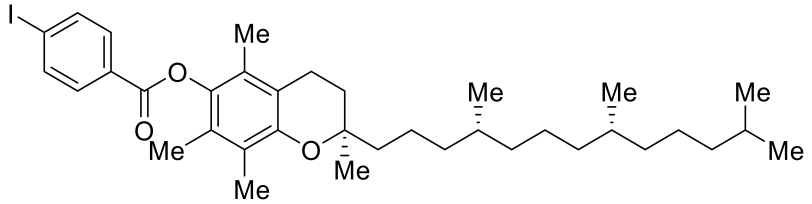

(*R*)-2,5,7,8-Tetramethyl-2-((4*R*,8*R*)-4,8,12-trimethyltridecyl)chroman-6-yl 4-iodobenzoate (2r)

Following general procedure A but at 40 °C instead of room temperature, α-tocopherol was used for the synthesis of compound **2s** which was obtained after column chromatography (hexane/EtOAc 250:1 to 25:1) as colorless oil (2.97 g, 90%).

^{1}H NMR (400 MHz, $CDCl_3$): δ = 7.97 (d, J = 8.5 Hz, 2H), 7.89 (d, J = 8.5 Hz, 2H), 2.63 (t, J = 6.8 Hz, 2H), 2.14 (s, 3H), 2.05 (s, 3H), 2.01 (s, 3H), 1.89 – 1.76 (m, 1H), 1.61 – 1.05 (m, 25H), 0.89 – 0.86 (m, 12H) ppm.

^{13}**C NMR** (101 MHz, $CDCl_3$): δ = 164.8 (C_q), 149.7 (C_q), 140.6 (C_q), 138.1 (CH), 131.7 (CH), 129.2 (C_q), 126.9 (C_q), 125.1 (C_q), 123.3 (C_q), 117.6 (C_q), 101.5 (C_q), 75.2 (C_q), 40.6 (CH_2), 39.8 (CH_2), 39.5 (CH_2), 37.6 (CH_2), 37.4 (CH_2), 32.9 (CH), 32.9 (CH), 32.8 (CH), 31.4 (CH_2), 31.1 (CH_2), 28.1 (CH_3), 25.0 (CH_2), 24.6 (CH_2), 24.3 (CH_3), 23.8 (CH_3), 22.9 (CH_3), 22.8 (CH_3), 21.2 (CH_2), 20.8 (CH_2), 19.9 (CH_3), 19.8 (CH_3), 13.2 (CH_3), 12.3 (CH_3), 12.0 (CH_3) ppm.

IR (ATR): $\tilde{\nu}$ = 2952, 2925, 1736, 1587, 1462, 1393, 1268, 1093, 1008, 751 cm^{-1}.

MS (ESI) *m*/*z* (relative intensity): 1343 (55) $[2M+Na]^+$, 683 (100) $[M+Na]^+$

HR MS (ESI): *m*/*z* calcd. for $C_{36}H_{53}INaO_3^+$ $[M+Na]^+$ 683.2932, found 683.2937.

4 Optimization studies

Table S1. Optimization of photoredox catalyst.

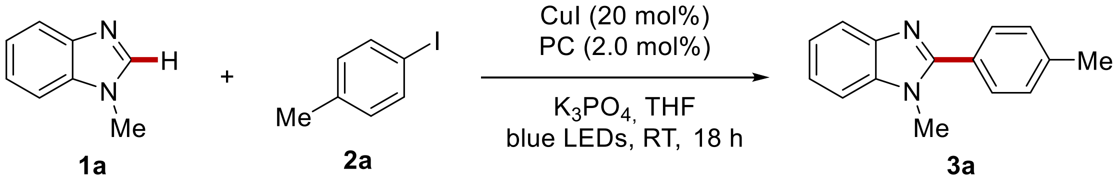

Entry	Photoredox catalyst	Yield (%)[a]
1	[Ir(ppy)$_3$]	11
2	[Ir(*p*-F-ppy)$_3$]	33
3	[Ru(bpy)$_3$]Cl$_2$ · 6 H$_2$O	5[b]
4	[Ru(bpy)$_3$](PF$_6$)$_2$	0[b]
5	[Cu(dmp)$_2$]Cl	0[b]
6	[Mes-Acr]BF$_4$	7[b]
7	Eosin Y	12[b]
8	DDQ	0[b]

[a] Reaction conditions: **1a** (0.25 mmol), **2a** (0.75 mmol), CuI (20 mol%), photoredox catalyst (PC) (2.0 mol%), K_3PO_4 (0.75 mmol), THF (1 mL), RT, 18 h, under N_2, blue LEDs; yield of isolated product. [b] Yield determined by ^{1}H NMR spectroscopy using 1,3,5-trimethoxybenzene as internal standard. ppy = 2-Phenylpyridine. bpy = 2,2'-Bipyridine. dmp = 2,9-Dimethyl-1,10-phenantroline. Mes-Acr = 9-Mesityl-10-methylacridinium. DDQ = 2,3-Dichloro-5,6-dicyano-1,4-benzoquinone.

Table S2. Optimization of solvent.

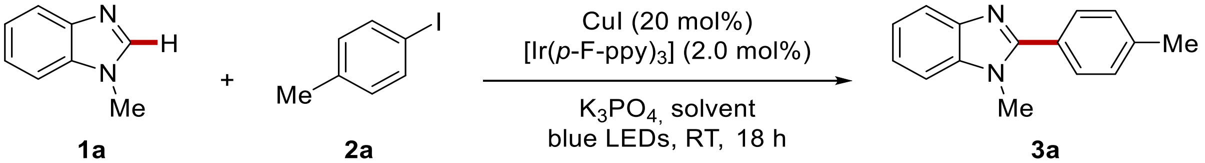

Entry	Solvent	Yield (%)[a]
1	THF	38
2	Et$_2$O	11[b]
3	1,4-dioxane	4[b]
4	DCE	0[b]
5	DMF	0[b]

6	DMA	0[b]
7	MeCN	7[b]
8	DMA/THF (1:1)	0[b]
9	MeCN/THF (3:1)	68
10	MeCN/THF (2:1)	71
11	MeCN/THF (1:1)	59
12	MeCN/THF (1:2)	65
13	MeCN/THF (1:3)	60

[a] Reaction conditions: **1a** (0.25 mmol), **2a** (0.75 mmol), CuI (20 mol%), [Ir(*p*-F-ppy)$_3$] (2.0 mol%), K_3PO_4 (0.75 mmol), solvent (1 mL), RT, 18 h, under N_2, blue LEDs; yield of isolated products. [b] Yield determined by ^{1}H NMR spectroscopy using 1,3,5-trimethoxybenzene as the internal standard.

Table S3. Optimization of copper source.

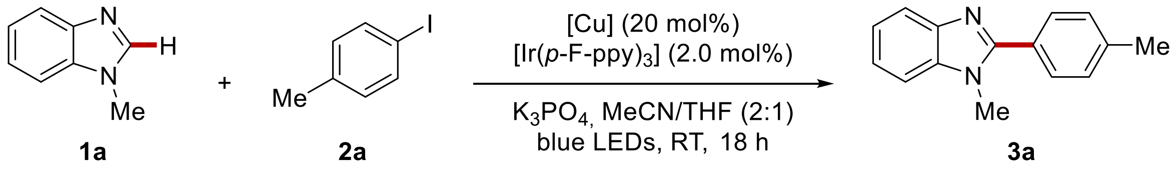

Entry	[Cu]	Yield (%)[a]
1	CuI	71
2	CuBr	51
3	CuOAc	63
4	CuTC	62
5	[Cu(MeCN)$_4$]PF$_6$	71
6	Cu_2O	0[b]

[a] Reaction conditions: **1a** (0.25 mmol), **2a** (0.75 mmol), [Cu] (20 mol%), [Ir(*p*-F-ppy)$_3$] (2.0 mol%), K_3PO_4 (0.75 mmol), MeCN/THF (2:1) (1 mL), RT, 18 h, under N_2, blue LEDs; yield of isolated products. [b] Yield determined by ^{1}H NMR spectroscopy using 1,3,5-trimethoxybenzene as internal standard. TC = thiophene-2-carboxylate.

Table S4. Optimization of base.

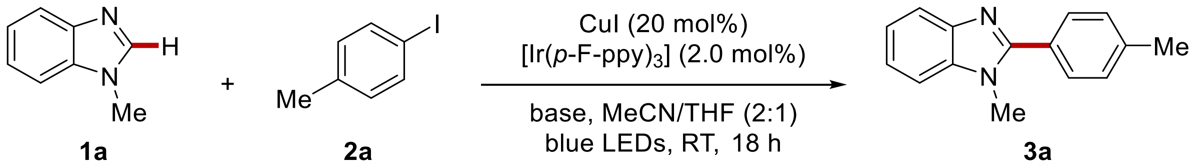

Entry	Base	Yield (%)[a]
1	K_3PO_4	71
2	K_2CO_3	55
3	Cs_2CO_3	36[b]
4	LiO*t*Bu	0[b]
5	KO*t*Bu	0[b]
6	KOAc	0[b]
7	KOPiv	0[b]
8	DIPEA	0[b]

[a] Reaction conditions: **1a** (0.25 mmol), **2a** (0.75 mmol), CuI (20 mol%), [Ir(*p*-F-ppy)3] (2.0 mol%), base (0.75 mmol), MeCN/THF (2:1) (1 mL), RT, 18 h, under N_2, blue LEDs; yield of isolated products. [b] Yield determined by ^{1}H NMR spectroscopy using 1,3,5-trimethoxybenzene as internal standard. Piv = Pivaloyl. DIPEA = *N*,*N*-Diisopropylethylamine.

Table S5. Optimization of photoredox catalyst, catalyst loading and excess of aryl halide.

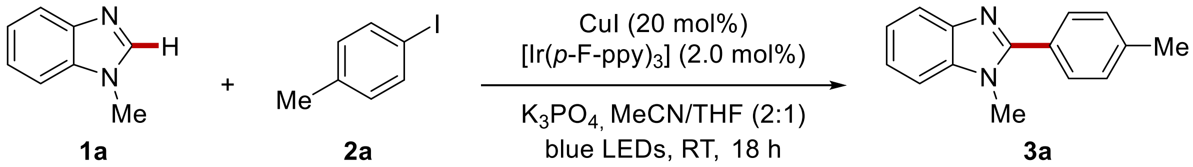

Entry	Deviation from standard conditions	Yield (%)[a]
1	---	71
2	PTH instead of [Ir(*p*-F-ppy)3]	77[b]
3	15 mol % CuI	57
4	10 mol % CuI	40
5	2.0 equiv. **2a**	57
6	1.5 equiv. **2a**	55
7	1.2 equiv. **2a**	39
8	**S1** instead of **2a**	17[c]
9	**S2** instead of **2a**	0[c]

10	**S1** instead of **2a** and PTH instead of [Ir(*p*-F-ppy)$_3$]	45^{b,c}
11	**S2** instead of **2a** and PTH instead of [Ir(*p*-F-ppy)$_3$]	0^{b,c}
12	**S3** instead of **2a** and PTH instead of [Ir(*p*-F-ppy)$_3$]	0^{b,c}
13	**S4** instead of **2a** and PTH instead of [Ir(*p*-F-ppy)$_3$]	0^{b,c}

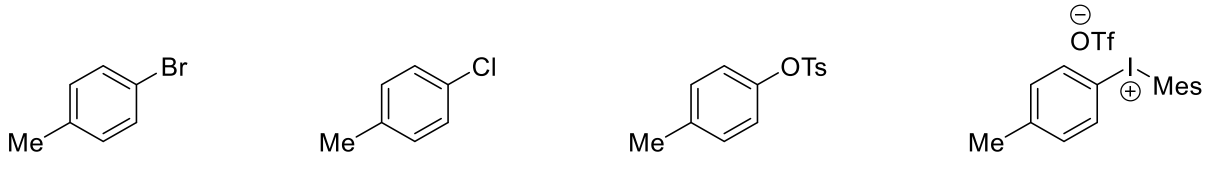

S1 **S2** **S3** **S4**

^{a} Reaction conditions: **1a** (0.25 mmol), **2a** (0.75 mmol), CuI (20 mol%), [Ir(*p*-F-ppy)$_3$] (2.0 mol%), K_3PO_4 (0.75 mmol), MeCN/THF (2:1) (1 mL), RT, 18 h, under N_2, blue LEDs (450 nm); yield of isolated products. ^{b} PTH (5.0 mol%), violet LEDs (390 nm). ^{c} Yield determined by ^{1}H NMR spectroscopy using 1,3,5-trimethoxybenzene as internal standard. PTH = 10-Phenylphenothiazine. Tol = Tolyl.

Table S6. Control experiments.

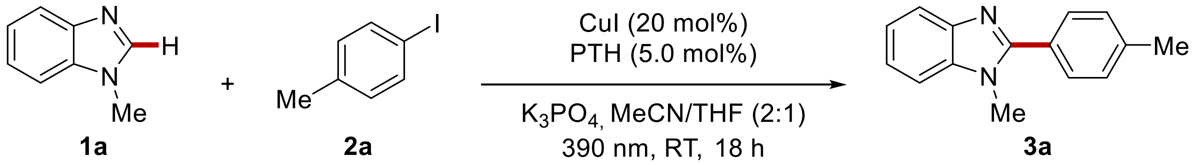

1a **2a** **3a**

Entry	Deviation from standard conditions	Yield (%)^a
1	---	77
2	no CuI	0^b
3	no photocatalyst (450 nm)	0^b
4	no photocatalyst (390 nm)	44
5	no light	0^b

^{a} Reaction conditions: **1a** (0.25 mmol), **2a** (0.75 mmol), CuI (20 mol%), [Ir(*p*-F-ppy)$_3$] (2.0 mol%), K_3PO_4 (0.75 mmol), MeCN/THF (2:1) (1 mL), RT, 18 h, under N_2, 390 nm; yield of isolated products. ^{b} Yield determined by ^{1}H NMR spectroscopy using 1,3,5-trimethoxybenzene as internal standard.

5 Light-induced copper-catalyzed C–H arylation

General procedure B: Light-induced copper-catalyzed C–H arylation

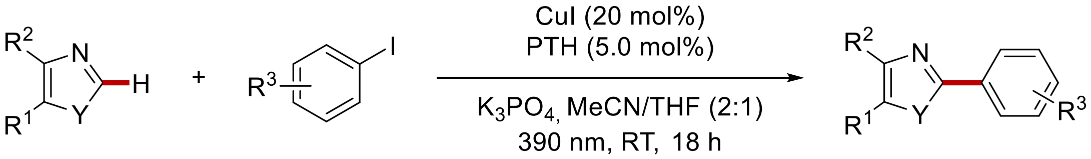

The corresponding azole (0.25 mmol, 1.0 equiv.), aryl iodide (0.75 mmol, 3.0 equiv.), CuI (9.5 mg, 50 µmol, 20 mol%), 10-phenylphenothiazine (PTH) (3.4 mg, 12.5 µmol, 5.0 mol%) and K_3PO_4 (159 mg, 0.75 mmol, 3.0 equiv.) were filled in in an oven-dried 10 mL vial equipped with a teflon-coated stir bar. The vial was sealed with a rubber septum and was evacuated and purged with N_2 three times before the addition of MeCN (0.67 mL) and THF (0.33 mL). Liquid starting materials were added before the solvent. Afterwards, the vial was wrapped with parafilm and the reaction mixture was stirred under violet light irradiation (390 nm, 2 × Kessil PR160L, internal temperature of the reaction mixture was determined to be 35 °C). After 18 h, the mixture was filtered through a pad of silica gel which was washed with EtOAc. The filtrate was concentrated *in vacuo*. Purification of the residue via column chromatography on silica gel yielded the arylated azoles.

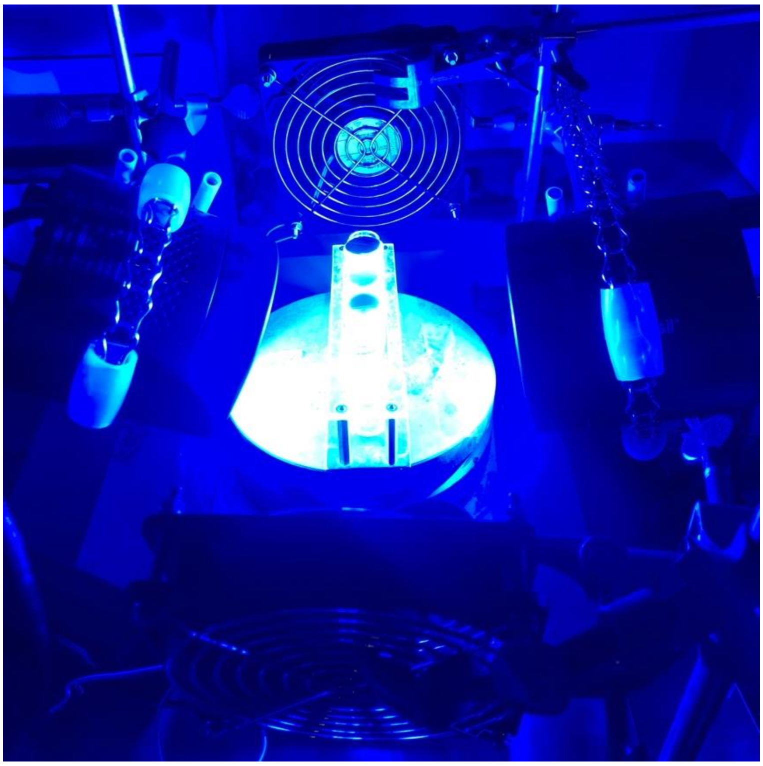

Figure S1. Reaction setup equipped with 2 × Kessil lamps A360N and 2 × fans.

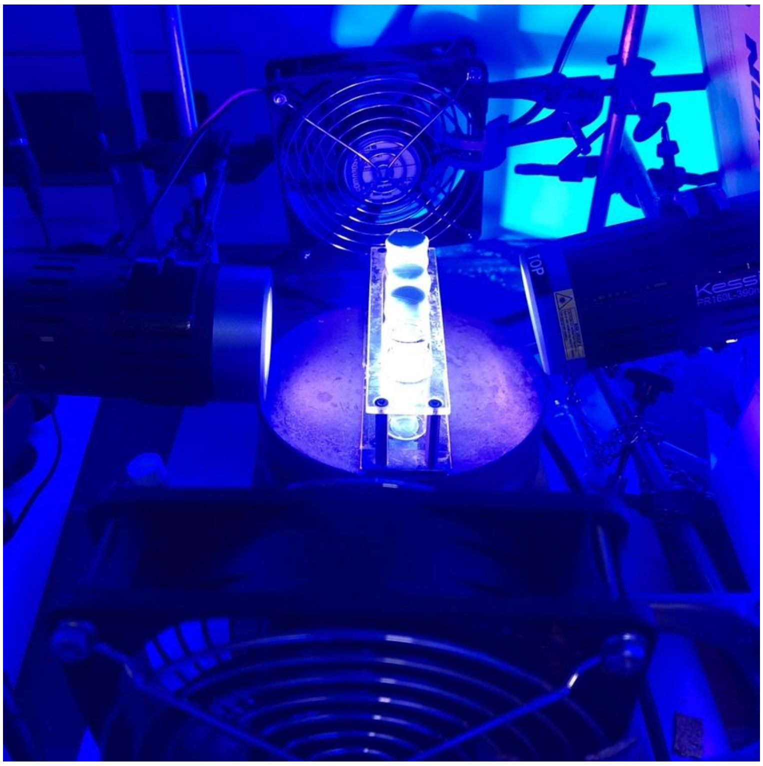

Figure S2. Reaction setup equipped with 2 × Kessil lamps PR160L and 2 × fans.

6 Characterization data

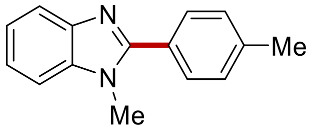

1-Methyl-2-(*p*-tolyl)-1*H*-benzo[*d*]imidazole (3a)

The general procedure B was followed using 1-methylbenzimidazole (33.0 mg, 0.25 mmol) and 4-iodotoluene (164 mg, 0.75 mmol). Purification by column chromatography on silica gel (hexane/EtOAc 5:1) yielded **3a** (42.8 mg, 77%) as yellow solid.

M.p.: 118 °C.

^{1}H NMR (400 MHz, $CDCl_3$): δ = 7.84 – 7.80 (m, 1H), 7.69 – 7.63 (m, 2H), 7.39 – 7.36 (m, 1H), 7.34 – 7.29 (m, 4H), 3.84 (s, 3H), 2.44 (s, 3H) ppm.

^{13}C NMR (101 MHz, $CDCl_3$): δ = 154.1 (C_q), 143.1 (C_q), 140.0 (C_q), 136.7 (C_q), 129.5 (CH), 129.4 (CH), 127.4 (C_q), 122.7 (CH), 122.4 (CH), 119.9 (CH), 109.6 (CH), 31.8 (CH_3), 21.6 (CH_3) ppm.

IR (ATR): $\tilde{\nu}$ = 1612, 1438, 1381, 831, 820, 747, 725, 700, 570, 498 cm^{-1}.

MS (ESI) *m*/*z* (relative intensity): 467 (8) $[2M+Na]^+$, 245 (7) $[M+Na]^+$, 223 (100) $[M+H]^+$.

HRMS (ESI): *m*/*z* calcd. for $C_{15}H_{15}N_2^+$ $[M+H]^+$ 223.1230, found 223.1233.

The spectral data are in accordance with those reported in the literature.[13]

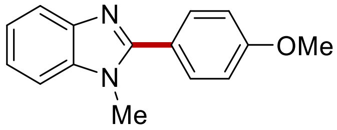

2-(4-Methoxyphenyl)-1-methyl-1*H*-benzo[*d*]imidazole (3b)

The general procedure B was followed using 1-methylbenzimidazole (33.0 mg, 0.25 mmol) and 4-iodoanisole (175 mg, 0.75 mmol). Purification by column chromatography on silica gel (hexane/EtOAc 4:1) yielded **3b** (34.6 mg, 58%) as yellow solid.

M.p.: 116 °C.

^{1}H NMR (400 MHz, $CDCl_3$): δ = 7.86 – 7.77 (m, 1H), 7.73 (d, J = 8.8 Hz, 2H), 7.43 – 7.34 (m, 1H), 7.34 – 7.28 (m, 2H), 7.05 (d, J = 8.9 Hz, 2H), 3.89 (s, 3H), 3.86 (s, 3H) ppm.

^{13}C NMR (101 MHz, $CDCl_3$): δ = 160.9 (C_q), 154.0 (C_q), 143.2 (C_q), 136.8 (C_q), 131.0 (CH), 122.8 (C_q), 122.6 (CH), 122.4 (CH), 119.8 (CH), 114.3 (CH), 109.6 (CH), 55.6 (CH_3), 31.9 (CH_3) ppm.

IR (ATR): $\tilde{\nu}$ = 1705, 1612, 1461, 1434, 1281, 1252, 1176, 1025, 838, 746 cm^{-1}.

MS (ESI) *m*/*z* (relative intensity): 499 (40) $[2M+Na]^+$, 261 (35) $[M+Na]^+$, 239 (100) $[M+H]^+$.

HRMS (ESI): *m*/*z* calcd. for $C_{15}H_{15}N_2O^+$ $[M+H]^+$ 239.1179, found 239.1184.

The spectral data are in accordance with those reported in the literature.[13]

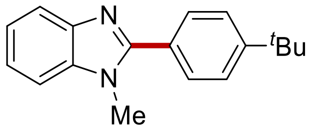

2-[4-(*tert*-Butyl)phenyl]-1-methyl-1*H*-benzo[*d*]imidazole (3c)

The general procedure B was followed using 1-methylbenzimidazole (33.0 mg, 0.25 mmol) and 1-(*tert*-butyl)-4-iodobenzene (195 mg, 0.75 mmol). Purification by column chromatography on silica gel (hexane/EtOAc 4:1) yielded **3c** (35.0 mg, 53%) as colorless solid.

M.p.: 154 °C.

^{1}H NMR (400 MHz, $CDCl_3$): δ = 7.85 – 7.81 (m, 1H), 7.71 (d, *J* = 8.5 Hz, 2H), 7.54 (d, *J* = 8.4 Hz, 2H), 7.40 – 7.36 (m, 1H), 7.33 – 7.28 (m, 2H), 3.86 (s, 3H), 1.38 (s, 9H) ppm.

^{13}C NMR (101 MHz, $CDCl_3$): δ = 154.0 (C_q), 153.1 (C_q), 143.2 (C_q), 136.7 (C_q), 129.3 (CH), 127.4 (C_q), 125.7 (CH), 122.7 (CH), 122.4 (CH), 119.9 (CH), 109.6 (CH), 35.0 (C_q), 31.8 (CH_3), 31.4 (CH_3) ppm.

IR (ATR): $\tilde{\nu}$ = 2947, 1613, 1485, 1461, 1435, 1379, 1326, 837, 746, 735 cm^{-1}.

MS (ESI) *m*/*z* (relative intensity): 551 (12) $[2M+Na]^+$, 529 (5) $[2M+H]^+$, 287 (10) $[M+Na]^+$, 265 (100) $[M+H]^+$.

HRMS (ESI): *m*/*z* calcd. for $C_{18}H_{21}N_2^+$ $[M+H]^+$ 265.1699, found 265.1704.

The spectral data are in accordance with those reported in the literature.[14]

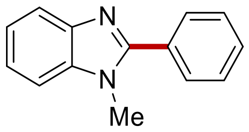

1-Methyl-2-phenyl-1*H*-benzo[*d*]imidazole (3d)

The general procedure B was followed using 1-methylbenzimidazole (33.0 mg, 0.25 mmol) and 4-iodobenzene (153 mg, 0.75 mmol). Purification by column chromatography on silica gel (hexane/EtOAc 5:1) yielded **3d** (31.2 mg, 60%) as brown solid.

M.p.: 68 °C.

^{1}H NMR (400 MHz, $CDCl_3$): δ = 7.86 – 7.82 (m, 1H), 7.79 – 7.74 (m, 2H), 7.55 – 7.49 (m, 3H), 7.41 – 7.37 (m, 1H), 7.35 – 7.30 (m, 2H), 3.84 (s, 3H) ppm.

^{13}**C NMR** (101 MHz, $CDCl_3$): δ = 153.9 (C_q), 143.1 (C_q), 136.7 (C_q), 130.3 (C_q), 129.8 (CH), 129.5 (CH), 128.8 (CH), 122.8 (CH), 122.5 (CH), 119.9 (CH), 109.7 (CH), 31.7 (CH_3) ppm.

IR (ATR): $\tilde{\nu}$ = 3053, 1469, 1461, 1441, 1379, 1327, 1022, 776, 733, 694 cm^{-1}.

MS (ESI) *m*/*z* (relative intensity): 439 (40) [2M+Na]$^+$, 231 (34) [M+Na]$^+$, 209 (100) [M+H]$^+$.

HRMS (ESI): *m*/*z* calcd. for $C_{14}H_{13}N_2^+$ [M+H]$^+$ 209.1073, found 209.1083.

The spectral data are in accordance with those reported in the literature.[15]

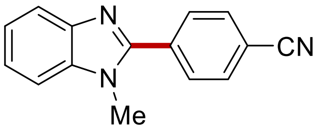

4-(1-Methyl-1*H*-benzo[*d*]imidazol-2-yl)benzonitrile (3e)

The general procedure B was followed using 1-methylbenzimidazole (33.0 mg, 0.25 mmol) and 4-iodobenzonitrile (172 mg, 0.75 mmol). Purification by column chromatography on silica gel (hexane/EtOAc 2:1) yielded **3e** (30.9 mg, 60%) as colorless solid.

M.p.: 203 °C.

^{1}**H NMR** (400 MHz, $CDCl_3$): δ = 7.93 (d, *J* = 8.1 Hz, 2H), 7.85 – 7.82 (m, 3H), 7.43 (dd, *J* = 7.2, 2.0 Hz, 1H), 7.40 – 7.33 (m, 2H), 3.91 (s, 3H) ppm.

^{13}**C NMR** (101 MHz, $CDCl_3$): δ = 151.6 (C_q), 143.1 (C_q), 136.9 (C_q), 134.8 (C_q), 132.6 (CH), 130.1 (CH), 123.8 (CH), 123.2 (CH), 120.4 (CH), 118.4 (C_q), 113.5 (C_q), 110.0 (CH), 32.0 (CH_3) ppm.

IR (ATR): $\tilde{\nu}$ = 2224, 1611, 1461, 1437, 1408, 1328, 1278, 1051, 845, 749 cm^{-1}.

MS (ESI) *m*/*z* (relative intensity): 256 (35) [M+Na]$^+$, 234 (100) [M+H]$^+$.

HRMS (ESI): *m*/*z* calcd. for $C_{15}H_{12}N_3^+$ [M+H]$^+$ 234.1026, found 234.1030.

The spectral data are in accordance with those reported in the literature.[16]

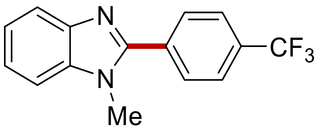

1-Methyl-2-[4-(trifluoromethyl)phenyl]-1*H*-benzo[*d*]imidazole (3f)

The general procedure B was followed using 1-methylbenzimidazole (33.0 mg, 0.25 mmol) and 1-iodo-4-(trifluoromethyl)benzene (204 mg, 0.75 mmol). Purification by column chromatography on silica gel (hexane/EtOAc 4:1) yielded **3f** (37.3 mg, 54%) as colorless solid.

M.p.: 104 °C.

^{1}**H NMR** (400 MHz, $CDCl_3$): δ = 7.91 (d, J = 8.1 Hz, 2H), 7.86 – 7.82 (m, 1H), 7.79 (d, J = 8.1 Hz, 2H), 7.43 – 7.39 (m, 1H), 7.38 – 7.31 (m, 2H), 3.88 (s, 3H) ppm.

^{13}**C NMR** (101 MHz, $CDCl_3$): δ = 152.2 (C_q), 143.1 (C_q), 136.8 (C_q), 134.0 (C_q), 131.7 (q, J = 32.7 Hz, C_q), 130.0 (CH), 125.8 (q, J = 3.6 Hz, CH), 124.0 (q, J = 273.2 Hz, C_q), 123.5 (CH), 123.0 (CH), 120.3 (CH), 109.9 (CH), 31.9 (CH_3) ppm.

^{19}**F NMR** (377 MHz, $CDCl_3$): δ = –62.8 ppm.

IR (ATR): $\tilde{\nu}$ = 1619, 1462, 1324, 1170, 1124, 1072, 1016, 850, 744, 420 cm^{-1}.

MS (ESI) *m*/*z* (relative intensity): 575 (55) $[2M+Na]^+$, 299 (38) $[M+Na]^+$, 277 (100) $[M+H]^+$.

HRMS (ESI): *m*/*z* calcd. for $C_{15}H_{12}F_3N_2^+$ $[M+H]^+$ 277.0947, found 277.0955.

The spectral data are in accordance with those reported in the literature.[15]

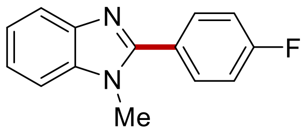

2-(4-Fluorophenyl)-1-methyl-1*H*-benzo[*d*]imidazole (3g)

The general procedure B was followed using 1-methylbenzimidazole (33.0 mg, 0.25 mmol) and 1-fluoro-4-iodobenzene (166 mg, 0.75 mmol). Purification by column chromatography on silica gel (hexane/EtOAc 6:1) yielded **3g** (19.8 mg, 35%) as brown solid.

M.p.: 96 °C.

^{1}**H NMR** (400 MHz, $CDCl_3$): δ = 7.84 – 7.80 (m, 1H), 7.76 (dd, J = 8.8, 5.3 Hz, 2H), 7.41 – 7.38 (m, 1H), 7.36 – 7.29 (m, 2H), 7.22 (dd, J = 8.7, 8.7 Hz, 2H), 3.85 (s, 3H) ppm.

^{13}**C NMR** (101 MHz, $CDCl_3$): δ = 163.8 (d, J = 250.3 Hz, C_q), 153.0 (C_q), 143.0 (C_q), 136.7 (C_q), 131.5 (d, J = 8.4 Hz, CH), 126.6 (d, J = 3.6 Hz, C_q), 123.0 (CH), 122.7 (CH), 120.0 (CH), 116.0 (d, J = 21.8 Hz, CH), 109.8 (CH), 31.8 (CH_3) ppm.

^{19}**F NMR** (377 MHz, $CDCl_3$): δ = –110.6 ppm.

IR (ATR): $\tilde{\nu}$ = 1606, 1486, 1460, 1437, 1380, 1278, 1223, 1158, 841, 743 cm^{-1}.

MS (ESI) *m*/*z* (relative intensity): 475 (45) $[2M+Na]^+$, 249 (38) $[M+Na]^+$, 227 (100) $[M+H]^+$.

HRMS (ESI): *m*/*z* calcd. for $C_{14}H_{12}FN_2^+$ $[M+H]^+$ 227.0979, found 227.0982.

The spectral data are in accordance with those reported in the literature.[13]

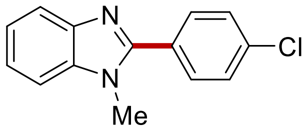

2-(4-Chlorophenyl)-1-methyl-1*H*-benzo[*d*]imidazole (3h)

The general procedure B was followed using 1-methylbenzimidazole (33.0 mg, 0.25 mmol) and 1-chloro-4-iodobenzene (179 mg, 0.75 mmol). Purification by column chromatography on silica gel (hexane/EtOAc 6:1) yielded **3h** (14.0 mg, 23%) as brown solid.

M.p.: 107 °C.

^{1}H NMR (300 MHz, $CDCl_3$): δ = 7.84 – 7.79 (m, 1H), 7.70 (d, *J* = 8.5 Hz, 2H), 7.50 (d, *J* = 8.5 Hz, 2H), 7.40 – 7.29 (m, 3H), 3.84 (s, 3H) ppm.

^{13}C NMR (101 MHz, $CDCl_3$): δ = 152.7 (C_q), 143.1 (C_q), 136.7 (C_q), 136.2 (C_q), 130.9 (CH), 129.2 (CH), 128.9 (C_q), 123.2 (CH), 122.8 (CH), 120.1 (CH), 109.8 (CH), 31.9 (CH_3) ppm.

IR (ATR): $\tilde{\nu}$ = 2925, 1470, 1459, 1434, 1402, 1326, 1092, 1013, 836, 741 cm^{-1}.

MS (ESI) *m*/*z* (relative intensity): 509 (65) $[M(^{35}Cl)+M(^{37}Cl)+Na]^+$, 507 (100) $[M(^{35}Cl)+M(^{35}Cl)+Na]^+$, 267 (20) $[M(^{37}Cl)+Na]^+$, 265 (65) $[M(^{35}Cl)+Na]^+$, 245 (12) $[M(^{37}Cl)+H]^+$, 243 (40) $[M(^{35}Cl)+H]^+$.

HRMS (ESI): *m*/*z* calcd. for $C_{14}H_{11}N_2{}^{35}ClNa^+$ $[M+Na]^+$ 265.0503, found 265.0505.

The spectral data are in accordance with those reported in the literature.[16]

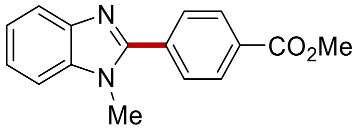

Methyl 4-(1-methyl-1*H*-benzo[*d*]imidazol-2-yl)benzoate (3i)

The general procedure B was followed using 1-methylbenzimidazole (33.0 mg, 0.25 mmol) and methyl 4-iodobenzoate (195 mg, 0.75 mmol). Purification by column chromatography on silica gel (hexane/EtOAc 3:1) yielded **3i** (38.6 mg, 58%) as brown solid.

M.p.: 114 °C.

^{1}H NMR (400 MHz, $CDCl_3$): δ = 8.19 (d, *J* = 8.3 Hz, 2H), 7.86 (d, *J* = 8.2 Hz, 2H), 7.85 – 7.82 (m, 1H), 7.43 – 7.39 (m, 1H), 7.38 – 7.30 (m, 2H), 3.96 (s, 3H), 3.88 (s, 3H) ppm.

^{13}C NMR (101 MHz, $CDCl_3$): δ = 166.7 (C_q), 152.7 (C_q), 143.1 (C_q), 136.8 (C_q), 134.7 (C_q), 131.2 (C_q), 130.0 (CH), 129.6 (CH), 123.4 (CH), 122.9 (CH), 120.3 (CH), 109.9 (CH), 52.5 (CH_3), 32.0 (CH_3) ppm.

IR (ATR): $\tilde{\nu}$ = 2950, 1722, 1612, 1461, 1434, 1274, 1109, 1017, 864, 746 cm^{-1}.

MS (ESI) *m*/*z* (relative intensity): 555 (100) $[2M+Na]^+$, 289 (56) $[M+Na]^+$, 267 (100) $[M+H]^+$.

HRMS (ESI): *m*/*z* calcd. for $C_{16}H_{15}N_2O_2^+$ [M+H]$^+$ 267.1128, found 267.1130.

The spectral data are in accordance with those reported in the literature.[16]

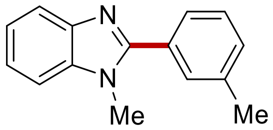

1-Methyl-2-(*m*-tolyl)-1*H*-benzo[*d*]imidazole (3j)

The general procedure B was followed using 1-methylbenzimidazole (33.0 mg, 0.25 mmol) and 3-iodotoluene (164 mg, 0.75 mmol). Purification by column chromatography on silica gel (hexane/EtOAc 5:1) yielded **3j** (33.3 mg, 60%) as brown oil.

^{1}H NMR (400 MHz, $CDCl_3$): δ = 7.88 – 7.78 (m, 1H), 7.63 (s, 1H), 7.52 (d, *J* = 7.6 Hz, 1H), 7.43 – 7.35 (m, 2H), 7.34 – 7.28 (m, 3H), 3.85 (s, 3H), 2.45 (s, 3H) ppm.

^{13}C NMR (101 MHz, $CDCl_3$): δ = 154.1 (C_q), 143.1 (C_q), 138.7 (C_q), 136.7 (C_q), 130.6 (CH), 130.3 (CH), 130.2 (C_q), 128.5 (CH), 126.4 (CH), 122.8 (CH), 122.5 (CH), 119.9 (CH), 109.7 (CH), 31.8 (CH_3), 21.5 (CH_3) ppm.

IR (ATR): $\tilde{\nu}$ = 3050, 1483, 1458, 1436, 1377, 1326, 1287, 877, 790, 743 cm^{-1}.

MS (ESI) *m*/*z* (relative intensity): 245 (15) [M+Na]$^+$, 223 (100) [M+H]$^+$.

HRMS (ESI): *m*/*z* calcd. for $C_{15}H_{15}N_2^+$ [M+H]$^+$ 223.1230, found 223.1234.

The spectral data are in accordance with those reported in the literature.[17]

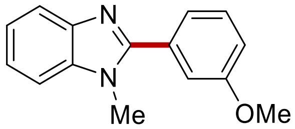

2-(3-Methoxyphenyl)-1-methyl-1*H*-benzo[*d*]imidazole (3k)

The general procedure B was followed using 1-methylbenzimidazole (33.0 mg, 0.25 mmol) and 3-iodoanisole (176 mg, 0.75 mmol). Purification by column chromatography on silica gel (hexane/EtOAc 5:1) yielded **3k** (42.8 mg, 72%) as yellow solid.

M.p.: 87 °C.

^{1}H NMR (400 MHz, $CDCl_3$): δ = 7.88 – 7.78 (m, 1H), 7.46 – 7.36 (m, 2H), 7.35 – 7.28 (m, 4H), 7.05 (ddd, *J* = 8.3, 2.6, 1.0 Hz, 1H), 3.88 (s, 3H), 3.86 (s, 3H) ppm.

^{13}C NMR (101 MHz, $CDCl_3$): δ = 159.9 (C_q), 153.7 (C_q), 143.0 (C_q), 136.7 (C_q), 131.6 (C_q), 129.8 (CH), 122.9 (CH), 122.6 (CH), 121.8 (CH), 120.0 (CH), 116.0 (CH), 114.8 (CH), 109.7 (CH), 55.6 (CH_3), 31.8 (CH_3) ppm.

IR (ATR): $\tilde{\nu}$ = 2938, 1706, 1579, 1453, 1376, 1238, 1033, 880, 735, 692 cm^{-1}.

MS (ESI) *m*/*z* (relative intensity): 499 (100) [2M+Na]$^+$, 261 (40) [M+Na]$^+$, 239 (95) [M+H]$^+$.
HRMS (ESI): *m*/*z* calcd. for $C_{15}H_{15}N_2O^+$ [M+H]$^+$ 239.1179, found 239.1186.
The spectral data are in accordance with those reported in the literature.[15]

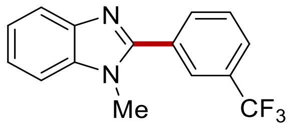

1-Methyl-2-(3-(trifluoromethyl)phenyl)-1*H*-benzo[*d*]imidazole (3l)
The general procedure B was followed using 1-methylbenzimidazole (33.0 mg, 0.25 mmol) and 1-iodo-3-(trifluoromethyl)benzene (204 mg, 0.75 mmol). Purification by column chromatography on silica gel (hexane/EtOAc 5:1) yielded **3l** (49.8 mg, 92%) as yellow oil.
^{1}H NMR (400 MHz, $CDCl_3$): δ = 8.07 (s, 1H), 7.96 (d, *J* = 8.2 Hz, 1H), 7.88 – 7.80 (m, 1H), 7.76 (d, *J* = 8.1 Hz, 1H), 7.66 (dd, *J* = 7.8, 7.8 Hz, 1H), 7.44 – 7.39 (m, 1H), 7.38 – 7.29 (m, 2H), 3.88 (s, 3H) ppm.
^{13}C NMR (101 MHz, $CDCl_3$): δ = 152.1 (C_q), 142.7 (C_q), 136.6 (C_q), 132.7 (CH), 131.3 (q, *J* = 32.8 Hz, C_q), 131.1 (C_q), 129.4 (CH), 126.6 (q, *J* = 3.6 Hz, CH), 126.4 (q, *J* = 3.6 Hz, CH), 123.5 (CH), 123.4 (q, *J* = 272.4 Hz, C_q), 123.0 (CH), 122.5 (CH), 119.8 (CH), 31.9 (CH_3) ppm.
^{19}F-NMR (377 MHz, $CDCl_3$): δ = –62.7 ppm.
IR (ATR): $\tilde{\nu}$ = 2357, 1733, 1717, 1699, 1684, 1419, 849, 808, 745, 699 cm^{-1}.
MS (ESI) *m*/*z* (relative intensity): 575 (30) [2M+Na]$^+$, 299 (15), 277 (100) [M+H]$^+$.
HRMS (ESI): *m*/*z* calcd. for $C_{15}H_{12}F_3N_2^+$ [M+H]$^+$ 277.0947, found 277.0954.
The spectral data are in accordance with those reported in the literature.[18]

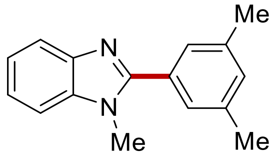

2-(3,5-Dimethylphenyl)-1-methyl-1*H*-benzo[*d*]imidazole (3m)
The general procedure B was followed using 1-methylbenzimidazole (33.0 mg, 0.25 mmol) and 1-iodo-3,5-dimethylbenzene (174 mg, 0.75 mmol). Purification by column chromatography on silica gel (hexane/EtOAc 5:1) yielded **3m** (28.9 mg, 49%) as orange oil.
^{1}H NMR (400 MHz, $CDCl_3$): δ = 7.87 – 7.78 (m, 1H), 7.40 – 7.35 (m, 3H), 7.33 – 7.29 (m, 2H), 7.14 (s, 1H), 3.85 (s, 3H), 2.40 (s, 6H) ppm.

^{13}**C NMR** (101 MHz, $CDCl_3$): δ = 154.2 (C_q), 143.1 (C_q), 138.4 (C_q), 136.7 (C_q), 131.5 (CH), 130.1 (C_q), 127.3 (CH), 122.7 (CH), 122.4 (CH), 119.9 (CH), 109.6 (CH), 31.8 (CH_3), 21.4 (CH_3) ppm.

IR (ATR): $\tilde{\nu}$ = 2918, 1601, 1473, 1440, 1375, 1324, 1283, 1006, 855, 742 cm^{-1}.

MS (ESI) *m*/*z* (relative intensity): 495 (30) $[2M+Na]^+$, 259 (20) $[M+Na]^+$, 237 (100) $[M+H]^+$.

HRMS (ESI): *m*/*z* calcd. for $C_{16}H_{17}N_2^+$ $[M+H]^+$ 237.1386, found 237.1390.

The spectral data are in accordance with those reported in the literature.[19]

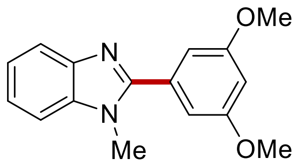

2-(3,5-Dimethoxyphenyl)-1-methyl-1*H*-benzo[*d*]imidazole (3n)

The general procedure B was followed using 1-methylbenzimidazole (33.0 mg, 0.25 mmol) and 1-iodo-3,5-dimethoxybenzene (198 mg, 0.75 mmol). Purification by column chromatography on silica gel (hexane/EtOAc 5:1) yielded **3n** (33.5 mg, 50%) as brown solid.

M.p.: 103 °C.

^{1}**H NMR** (400 MHz, $CDCl_3$): δ = 7.86 – 7.80 (m, 1H), 7.41 – 7.36 (m, 1H), 7.34 – 7.27 (m, 2H), 6.89 (d, *J* = 2.3 Hz, 2H), 6.60 (t, *J* = 2.3 Hz, 1H), 3.86 (s, 3H), 3.86 (s, 6H) ppm.

^{13}**C NMR** (101 MHz, $CDCl_3$): δ = 161.0 (C_q), 153.7 (C_q), 142.9 (C_q), 136.7 (C_q), 132.0 (C_q), 123.0 (CH), 122.6 (CH), 119.9 (CH), 109.8 (CH), 107.6 (CH), 102.1 (CH), 55.7 (CH_3), 31.9 (CH_3) ppm.

IR (ATR): $\tilde{\nu}$ = 1595, 1454, 1383, 1325, 1291, 1206, 1157, 1063, 1040, 692 cm^{-1}.

MS (ESI) *m*/*z* (relative intensity): 559 (30) $[2M+Na]^+$, 291 (20) $[M+Na]^+$, 269 (100) $[M+H]^+$.

HRMS (ESI): *m*/*z* calcd. for $C_{16}H_{17}N_2O_2^+$ $[M+H]^+$ 269.1285, found 269.1291.

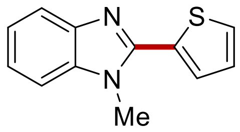

1-Methyl-2-(thiophen-2-yl)-1*H*-benzo[*d*]imidazole (3o)

The general procedure B was followed using 1-methylbenzimidazole (33.0 mg, 0.25 mmol) and 2-iodothiophene (158 mg, 0.75 mmol). Purification by column chromatography on silica gel (hexane/EtOAc 8:1 to 6:1) yielded **3o** (18.2 mg, 34%) as yellow oil.

^{1}H NMR (400 MHz, $CDCl_3$): δ = 7.86 – 7.74 (m, 1H), 7.58 (dd, J = 3.7, 1.1 Hz, 1H), 7.53 (dd, J = 5.0, 1.1 Hz, 1H), 7.39 – 7.35 (m, 1H), 7.33 – 7.28 (m, 2H), 7.20 (dd, J = 5.1, 3.7 Hz, 1H), 4.00 (s, 3H) ppm.

^{13}C NMR (101 MHz, $CDCl_3$): δ = 148.0 (C_q), 142.9 (C_q), 136.7 (C_q), 132.6 (C_q), 128.8 (CH), 128.2 (CH), 128.0 (CH), 123.1 (CH), 122.9 (CH), 119.9 (CH), 109.5 (CH), 31.8 (CH_3) ppm.

IR (ATR): $\tilde{\nu}$ = 2925, 1460, 1441, 1411, 1385, 1324, 1286, 1227, 740, 712 cm^{-1}.

MS (ESI) *m/z* (relative intensity): 237 (35) $[M+Na]^+$, 215 (100) $[M+H]^+$.

HRMS (ESI): *m/z* calcd. for $C_{12}H_{11}N_2S^+$ $[M+H]^+$ 215.0637, found 215.0642.

The spectral data are in accordance with those reported in the literature.[13]

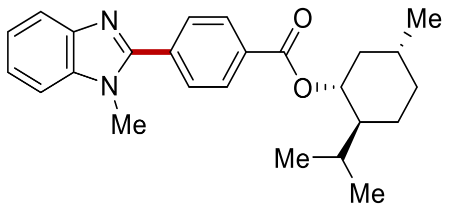

(1*R*,2*S*,5*R*)-2-Isopropyl-5-methylcyclohexyl 4-(1-methyl-1*H*-benzo[*d*]imidazol-2-yl)benzoate (3p)

The general procedure B was followed using 1-methylbenzimidazole (33.0 mg, 0.25 mmol) and (1*R*,2*S*,5*R*)-2-isopropyl-5-methylcyclohexyl 4-iodobenzoate (290 mg, 0.75 mmol). Purification by column chromatography on silica gel (hexane/EtOAc 7.5:1) yielded **3p** (49.8 mg, 51%) as colorless oil.

^{1}H NMR (400 MHz, $CDCl_3$): δ = 8.21 (d, J = 8.4 Hz, 2H), 7.89 – 7.84 (m, 3H), 7.45 – 7.41 (m, 1H), 7.40 – 7.32 (m, 2H), 4.98 (td, J = 10.9, 4.4 Hz, 1H), 3.91 (s, 3H), 2.21 – 2.11 (m, 1H), 1.99 (pd, J = 6.9, 2.7 Hz, 1H), 1.81 – 1.69 (m, 2H), 1.64 – 1.53 (m, 2H), 1.21 – 1.10 (m, 2H), 1.00 – 0.90 (m, 1H), 0.95 (d, J = 2.2 Hz, 3H), 0.94 (d, J = 2.7 Hz, 3H), 0.82 (d, J = 7.0 Hz, 3H) ppm.

^{13}C NMR (101 MHz, $CDCl_3$): δ = 165.6 (C_q), 152.7 (C_q), 142.8 (C_q), 136.7 (C_q), 134.2 (C_q), 132.1 (C_q), 130.0 (CH), 129.6 (CH), 123.5 (CH), 123.0 (CH), 120.2 (CH), 109.9 (CH), 75.5 (CH), 47.4 (CH), 41.1 (CH_2), 34.4 (CH_2), 32.0 (CH), 31.6 (CH), 26.7 (CH), 23.8 (CH_2), 22.2 (CH_3), 20.9 (CH_3), 16.7 (CH_3) ppm.

IR (ATR): $\tilde{\nu}$ = 2955, 1711, 1613, 1459, 1272, 1177, 1096, 1017, 778, 738 cm^{-1}.

MS (ESI) *m/z* (relative intensity): 781 (50) $[2M+H]^+$, 413 (14) $[M+Na]^+$, 391 (100) $[M+H]^+$.

HRMS (ESI): *m/z* calcd. for $C_{25}H_{31}N_2O_2^+$ $[M+H]^+$ 391.2380, found 391.2377.

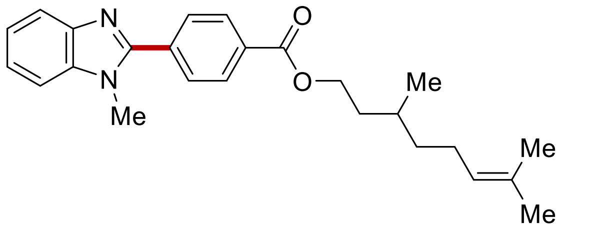

3,7-Dimethyloct-6-en-1-yl 4-(1-methyl-1*H*-benzo[*d*]imidazol-2-yl)benzoate (3q)

The general procedure B was followed using 1-methylbenzimidazole (33.0 mg, 0.25 mmol) and 3,7-dimethyloct-6-en-1-yl 4-iodobenzoate (290 mg, 0.75 mmol). Purification by column chromatography on silica gel (hexane/EtOAc 7.5:1) yielded **3q** (31.0 mg, 32%) as colorless oil.

^{1}H NMR (400 MHz, $CDCl_3$): δ = 8.19 (d, J = 8.4 Hz, 2H), 7.90 – 7.80 (m, 3H), 7.44 – 7.39 (m, 1H), 7.38 – 7.29 (m, 2H), 5.11 (m, 1H), 4.45 – 4.36 (m, 2H), 3.89 (s, 3H), 2.11 – 1.96 (m, 2H), 1.93 – 1.75 (m, 1H), 1.74 – 1.53 (m, 8H), 1.42 (m, 1H), 1.26 (m, 1H), 0.99 (d, J = 6.5 Hz, 3H) ppm.

^{13}C NMR (101 MHz, $CDCl_3$): δ = 166.2 (C_q), 152.7 (C_q), 143.1 (C_q), 136.8 (C_q), 134.5 (C_q), 131.6 (C_q), 131.6 (C_q), 130.0 (CH), 129.5 (CH), 124.7 (CH), 123.4 (CH), 122.9 (CH), 120.2 (CH), 109.9 (CH), 64.0 (CH_2), 37.1 (CH_2), 35.6 (CH_2), 31.9 (CH_3), 29.7 (CH_3), 25.9 (CH), 25.5 (CH_2), 19.7 (CH_3), 17.8 (CH_3) ppm.

IR (ATR): $\tilde{\nu}$ = 2910, 1713, 1714, 1461, 1379, 1268, 1178, 1097, 1017, 738 cm^{-1}.

MS (ESI) *m*/*z* (relative intensity): 781 (17) $[2M+H]^+$, 391 (100) $[M+H]^+$.

HRMS (ESI): *m*/*z* calcd. for $C_{25}H_{31}N_2O_2^+$ $[M+H]^+$ 391.2380, found 391.2370.

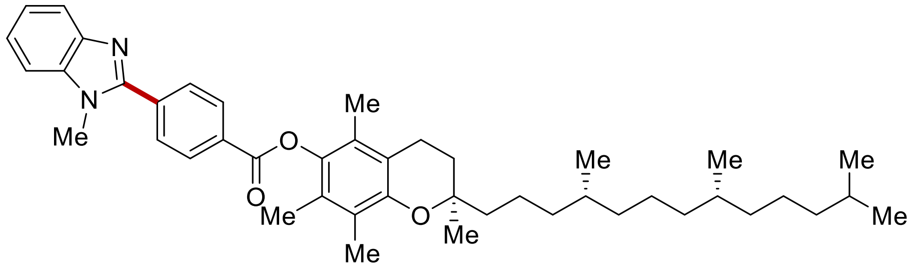

(*R*)-2,5,7,8-Tetramethyl-2-((4*R*,8*R*)-4,8,12-trimethyltridecyl)chroman-6-yl 4-(1-methyl-1*H*-benzo[*d*]imidazol-2-yl)benzoate (3r)

The general procedure B was followed using 1-methylbenzimidazole (33.0 mg, 0.25 mmol) and (*R*)-2,5,7,8-tetramethyl-2-((4*R*,8*R*)-4,8,12-trimethyltridecyl)chroman-6-yl 4-iodobenzoate (495 mg, 0.75 mmol). Purification by column chromatography on silica gel (hexane/EtOAc 5:1) yielded **3r** (66.5 mg, 40%) as colorless oil.

^{1}**H NMR** (500 MHz, $CDCl_3$): δ = 8.41 (d, J = 8.5 Hz, 2H), 7.96 (d, J = 8.6 Hz, 2H), 7.91 – 7.84 (m, 1H), 7.47 – 7.41 (m, 1H), 7.41 – 7.32 (m, 2H), 3.94 (s, 3H), 2.64 (t, J = 6.8 Hz, 2H), 2.14 (s, 3H), 2.09 (s, 3H), 2.05 (s, 3H), 1.88 – 1.81 (m, 1H), 1.69 – 1.49 (m, 5H), 1.44 – 1.36 (m, 3H), 1.32 – 1.22 (m, 11H), 1.17 – 1.06 (m, 6H), 0.90 – 0.83 (m, 12H).

^{13}**C NMR** (126 MHz, $CDCl_3$): δ = 164.8 (C_q), 152.6 (C_q), 149.7 (C_q), 143.2 (C_q), 140.7 (C_q), 136.9 (C_q), 135.1 (C_q), 130.7 (C_q), 130.6 (CH), 129.8 (CH), 127.0 (C_q), 125.2 (C_q), 123.5 (CH), 123.4 (C_q), 123.0 (CH), 120.3 (CH), 117.7 (C_q), 109.9 (CH), 75.3 (C_q), 40.6 (CH_2), 39.8 (CH_2), 39.5 (CH_2), 37.6 (CH_2), 37.4 (CH_2), 33.0 (CH), 32.9 (CH), 32.8 (CH), 32.0 (CH_3), 31.4 (CH_2), 31.2 (CH_2), 28.1 (CH_3), 25.0 (CH_2), 24.6 (CH_2), 24.4 (CH_3), 23.9 (CH_3), 22.9 (CH_3), 22.8 (CH_3), 21.2 (CH_2), 20.8 (CH_2), 19.9 (CH_3), 19.8 (CH_3), 13.3 (CH_3), 12.4 (CH_3), 12.0 (CH_3) ppm.

IR (ATR): $\tilde{\nu}$ = 2952, 2924, 1731, 1461, 1271, 1234, 1090, 1016, 862, 738 cm^{-1}.

MS (ESI) *m/z* (relative intensity): 1329 (35) $[2M+H]^+$, 687 (35) $[M+Na]^+$, 665 (100) $[M+H]^+$.

HRMS (ESI): *m/z* calcd. for $C_{44}H_{61}N_2O_3^+$ $[M+H]^+$ 665.4677, found 665.4669.

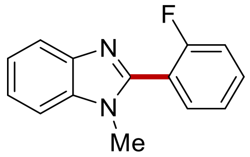

2-(2-Fluorophenyl)-1-methyl-1*H*-benzo[*d*]imidazole (3s)

The general procedure B was followed using 1-methylbenzimidazole (33.0 mg, 0.25 mmol) and 1-fluoro-2-iodobenzene (167 mg, 0.75 mmol). Purification by column chromatography on silica gel (hexane/EtOAc 6:1) yielded **3s** (6.8 mg, 12%) as yellow solid.

^{1}**H NMR** (400 MHz, $CDCl_3$): δ = 7.87 – 7.83 (m, 1H), 7.74 (ddd, J = 7.4, 7.4, 1.9 Hz, 1H), 7.56 – 7.50 (m, 1H), 7.45 – 7.42 (m, 1H), 7.38 – 7.31 (m, 3H), 7.27 – 7.22 (m, 1H), 3.76 (d, J = 2.5 Hz, 3H) ppm.

^{13}**C NMR** (101 MHz, $CDCl_3$): δ = 160.3 (d, J =250.0 Hz, C_q), 149.5 (C_q), 143.3 (C_q), 136.4 (C_q), 132.7 (d, J = 2.9 Hz, CH), 132.2 (d, J = 8.4 Hz, CH), 124.9 (d, J = 3.6 Hz, CH), 123.1 (CH), 122.6 (CH), 120.2 (CH), 118.6 (d, J = 14.7 Hz, C_q), 116.0 (d, J = 21.4 Hz, CH), 109.8 (CH), 31.2 (d, J = 5.8 Hz, CH_3) ppm.

^{19}**F NMR** (377 MHz, $CDCl_3$): δ = –113.1 ppm.

IR (ATR): $\tilde{\nu}$ = 2924, 2854, 1622, 1474, 1452, 1356, 1328, 1226, 765, 744 cm^{-1}.

MS (ESI) *m/z* (relative intensity): 475 (60) $[2M+Na]^+$, 249 (55) $[M+Na]^+$, 227 (100) $[M+H]^+$.

HRMS (ESI): *m/z* calcd. for $C_{14}H_{12}FN_2^+$ $[M+H]^+$ 227.0979, found 227.0986.

The spectral data are in accordance with those reported in the literature.[20]

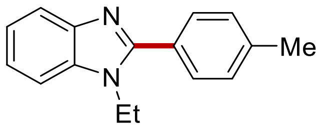

1-Ethyl-2-(*p*-tolyl)-1*H*-benzo[*d*]imidazole (5a)

The general procedure B was followed using 1-ethyl-1*H*-benzo[*d*]imidazole (36.5 mg, 0.25 mmol) and 4-iodotoluene (164 mg, 0.75 mmol). Purification by column chromatography on silica gel (hexane/EtOAc 5:1) yielded **5a** (45.0 mg, 76%) as yellow oil.

^{1}H NMR (400 MHz, $CDCl_3$): δ = 7.87 – 7.78 (m, 1H), 7.62 (d, *J* = 8.2 Hz, 2H), 7.47 – 7.38 (m, 1H), 7.35 – 7.29 (m, 4H), 4.29 (q, *J* = 7.2 Hz, 2H), 2.45 (s, 3H), 1.47 (t, *J* = 7.3 Hz, 3H) ppm.

^{13}C NMR (101 MHz, $CDCl_3$): δ = 153.8 (C_q), 143.3 (C_q), 134.0 (C_q), 135.5 (C_q), 129.6 (CH), 129.3 (CH), 127.8 (C_q), 122.7 (CH), 122.4 (CH), 120.0 (CH), 110.0 (CH), 39.7 (CH_2), 21.6 (CH_3), 15.4 (CH_3) ppm.

IR (ATR): $\tilde{\nu}$ = 2357, 1733, 1699, 1520, 1457, 1419, 824, 746, 503, 455 cm^{-1}.

MS (ESI) *m*/*z* (relative intensity): 495 (20) $[2M+Na]^+$, 473 (1) $[2M+H]^+$, 259 (20) $[M+Na]^+$, 237 (100) $[M+H]^+$.

HRMS (ESI): *m*/*z* calcd. for $C_{16}H_{17}N_2$ $[M+H]^+$ 237.1386, found 237.1388.

The spectral data are in accordance with those reported in the literature.[21]

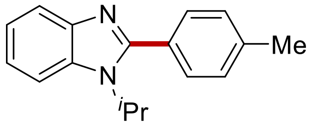

1-Isopropyl-2-(*p*-tolyl)-1*H*-benzo[*d*]imidazole (5b)

The general procedure B was followed using 1-isopropyl-1*H*-benzo[*d*]imidazole (40.1 mg, 0.25 mmol) and 4-iodotoluene (164 mg, 0.75 mmol). Purification by column chromatography on silica gel (hexane/EtOAc 5:1) yielded **5b** (40.1 mg, 64%) as brown solid.

M.p.: 156 °C.

^{1}H NMR (400 MHz, $CDCl_3$): δ = 7.86 – 7.79 (m, 1H), 7.68 – 7.59 (m, 1H), 7.55 (d, *J* = 8.1 Hz, 2H), 7.33 (d, *J* = 7.8 Hz, 2H), 7.31 – 7.22 (m, 2H), 4.84 (hept, *J* = 7.0 Hz, 1H), 2.45 (s, 3H), 1.65 (d, *J* = 7.0 Hz, 6H) ppm.

^{13}**C NMR** (101 MHz, $CDCl_3$): δ = 153.9 (C_q), 143.9 (C_q), 139.9 (C_q), 133.6 (C_q), 129.5 (CH), 129.5 (CH), 128.2 (C_q), 122.2 (CH), 122.1 (CH), 120.3 (CH), 112.4 (CH), 48.9 (CH), 21.6 (CH_3), 21.6 (CH_3) ppm.
IR (ATR): $\tilde{\nu}$ = 2357, 1733, 1717, 1699, 1684, 1457, 1260, 823, 748, 609 cm^{-1}.
MS (ESI) *m/z* (relative intensity): 773 (22) $[3M+Na]^+$, 523 (40) $[2M+Na]^+$, 501 (6) $[2M+H]^+$, 273 (25) $[M+Na]^+$, 251 (100) $[M+H]^+$.
HRMS (ESI): *m/z* calcd. for $C_{17}H_{19}N_2^+$ $[M+H]^+$ 251.1543, found 251.1550.

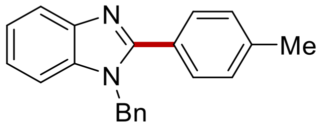

1-Benzyl-2-(*p*-tolyl)-1*H*-benzo[*d*]imidazole (5c)

The general procedure B was followed using 1-benzyl-1*H*-benzo[*d*]imidazole (52.1 mg, 0.25 mmol) and 4-iodotoluene (164 mg, 0.75 mmol). Purification by column chromatography on silica gel (hexane/EtOAc 5:1) yielded **5c** (46.3 mg, 62%) as colorless solid.
M.p.: 137 °C.
^{1}**H NMR** (400 MHz, $CDCl_3$): δ = 7.87 (d, *J* = 8.0 Hz, 1H), 7.59 (d, *J* = 8.1 Hz, 2H), 7.36 – 7.16 (m, 8H), 7.11 (d, *J* = 6.7 Hz, 2H), 5.45 (s, 2H), 2.41 (s, 3H) ppm.
^{13}**C NMR** (101 MHz, $CDCl_3$): δ = 154.5 (C_q), 143.4 (C_q), 140.2 (C_q), 136.7 (C_q), 136.2 (C_q), 129.6 (CH), 129.3 (CH), 129.2 (CH), 127.9 (CH), 127.3 (C_q), 126.1 (CH), 123.0 (CH), 122.7 (CH), 120.0 (CH), 110.6 (CH), 48.5 (CH_2), 21.6 (CH_3) ppm.
IR (ATR): $\tilde{\nu}$ = 3030, 1614, 1482, 1453, 1384, 1354, 822, 745, 728, 695 cm^{-1}.
MS (ESI) *m/z* (relative intensity): 619 (85) $[2M+Na]^+$, 321 (50) $[M+Na]^+$, 299 (100) $[M+H]^+$.
HRMS (ESI): *m/z* calcd. for $C_{21}H_{19}N_2^+$ $[M+H]^+$ 299.1543, found 299.1546.
The spectral data are in accordance with those reported in the literature.[22]

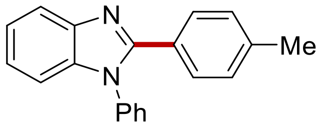

1-Phenyl-2-(*p*-tolyl)-1*H*-benzo[*d*]imidazole (5d)

The general procedure B was followed using 1-phenyl-1*H*-benzo[*d*]imidazole (48.6 mg, 0.25 mmol) and 4-iodotoluene (164 mg, 0.75 mmol). Purification by column chromatography on silica gel (hexane/EtOAc 5:1) yielded **5d** (49.8 mg, 70%) as yellow oil.

^{1}**H NMR** (400 MHz, $CDCl_3$): δ = 7.88 (d, *J* = 7.9 Hz, 1H), 7.56 – 7.42 (m, 5H), 7.33 (ddd, *J* = 8.0, 4.7, 1.8 Hz, 3H), 7.29 – 7.21 (m, 2H), 7.11 (d, *J* = 8.0 Hz, 2H), 2.34 (s, 3H) ppm.
^{13}**C NMR** (101 MHz, $CDCl_3$): δ = 152.7 (C_q), 143.2 (C_q), 139.7 (C_q), 137.3 (C_q), 137.3 (C_q), 130.0 (CH), 129.5 (CH), 129.2 (CH), 128.6 (CH), 127.6 (CH), 127.2 (C_q), 123.3 (CH), 123.0 (CH), 119.9 (CH), 110.5 (CH), 21.5 (CH_3) ppm.
IR (ATR): $\tilde{\nu}$ = 2357, 1717, 1699, 1655, 1323, 1250, 1185, 741, 606, 509 cm^{-1}.
MS (ESI) *m*/*z* (relative intensity): 875 (23) $[3M+Na]^+$, 591 (32) $[2M+Na]^+$, 569 (5) $[2M+H]^+$, 307 (14) $[M+Na]^+$, 285 (100) $[M+H]^+$.
HRMS (ESI): *m*/*z* calcd. for $C_{20}H_{16}N_2Na^+$ $[M+Na]^+$ 307.1206, found 307.1205.
The spectral data are in accordance with those reported in the literature.[23]

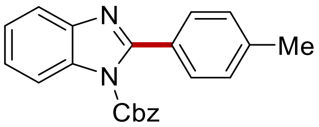

Benzyl 2-(*p*-tolyl)-1*H*-benzo[*d*]imidazole-1-carboxylate (5e)

The general procedure B was followed using benzyl 1*H*-benzo[*d*]imidazole-1-carboxylate (63.1 mg, 0.25 mmol) and 4-iodotoluene (164 mg, 0.75 mmol). Purification by column chromatography on silica gel (hexane/EtOAc 3:1) yielded **5e** (45.4 mg, 53%) as brown solid.
M.p.: 123 °C.
^{1}**H NMR** (400 MHz, $CDCl_3$): δ = 8.05 – 7.97 (m, 1H), 7.83 – 7.74 (m, 1H), 7.52 (d, *J* = 8.1 Hz, 2H), 7.41 – 7.30 (m, 5H), 7.22 – 7.19 (m, 4H), 5.33 (s, 2H), 2.41 (s, 3H) ppm.
^{13}**C NMR** (101 MHz, $CDCl_3$): δ = 154.2 (C_q), 150.3 (C_q), 142.9 (C_q), 140.0 (C_q), 134.0 (C_q), 133.7 (C_q), 129.4 (CH), 128.9 (CH), 128.9 (C_q), 128.8 (CH), 128.8 (CH), 128.7 (CH), 125.2 (CH), 124.8 (CH), 120.3 (CH), 115.0 (CH), 69.7 (CH_2), 21.6 (CH_3) ppm.
IR (ATR): $\tilde{\nu}$ = 2357, 1743, 1699, 1602, 1568, 1247, 1066, 822, 748, 697 cm^{-1}.
MS (ESI) *m*/*z* (relative intensity): 707 (98) $[2M+Na]^+$, 365 (92) $[M+Na]^+$, 343 (100) $[M+H]^+$.
HRMS (ESI): *m*/*z* calcd. for $C_{22}H_{19}N_2O_2^+$ $[M+H]^+$ 343.1441, found 343.1433.

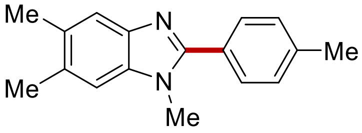

1,5,6-Trimethyl-2-(*p*-tolyl)-1*H*-benzo[*d*]imidazole (5f)

Following a modification of general procedure B, the title compound was obtained by using 1,5,6-trimethyl-1*H*-benzo[*d*]imidazole (40.1 mg, 0.25 mmol), 4-iodotoluene (218 mg, 1.00 mmol), $[Cu(MeCN)_4]PF_6$ (28.0 mg, 75 µmol), 10-phenylphenothiazine (PTH) (3.4 mg, 12.5 µmol) and K_3PO_4 (159 mg, 0.75 mmol, 3.0 equiv.) in MeCN/THF (2:1, 0.5 mL). After stirring for 48 h under 390 nm irradiation, the product **5f** was obtained as yellow solid (26.9 mg, 43%) through the purification by column chromatography on silica gel (hexane/EtOAc 5:1).

M.p.: 109 °C.

^{1}H NMR (400 MHz, $CDCl_3$): δ = 7.64 (d, J = 8.2 Hz, 2H), 7.57 (s, 1H), 7.31 (d, J = 7.9 Hz, 2H), 7.14 (s, 1H), 3.80 (s, 3H), 2.43 (s, 3H), 2.42 (s, 3H), 2.40 (s, 3H) ppm.

^{13}C NMR (101 MHz, $CDCl_3$): δ = 153.2 (C_q), 141.6 (C_q), 139.7 (C_q), 135.3 (C_q), 131.9 (C_q), 131.3 (C_q), 129.4 (CH), 129.4 (CH), 127.6 (C_q), 119.9 (CH), 109.9 (CH), 31.8 (CH_3), 21.6 (CH_3), 20.7 (CH_3), 20.4 (CH_3) ppm.

IR (ATR): $\tilde{\nu}$ = 2918, 1481, 1461, 1437, 1381, 1321, 1019, 997, 823, 728 cm^{-1}.

MS (ESI) *m*/*z* (relative intensity): 523 (45) $[2M+Na]^+$, 273 (25) $[M+Na]^+$, 251 (100) $[M+H]^+$.

HRMS (ESI): *m*/*z* calcd. for $C_{17}H_{19}N_2^+$ $[M+H]^+$ 251.1543, found 251.1546.

The spectral data are in accordance with those reported in the literature.[13]

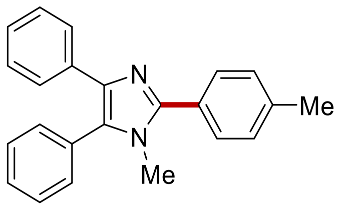

1-Methyl-4,5-diphenyl-2-(*p*-tolyl)-1*H*-imidazole (5g)

The general procedure B was followed using 1-methyl-4,5-diphenyl-1*H*-imidazole (58.6 mg, 0.25 mmol) and 4-iodotoluene (164 mg, 0.75 mmol). Purification by column chromatography on silica gel (hexane/EtOAc 5:1) yielded **5g** (37.0 mg, 46%) as yellow solid.

M.p.: 215 °C.

^{1}H NMR (400 MHz, $CDCl_3$): δ = 7.64 (d, J = 8.2 Hz, 2H), 7.57 – 7.53 (m, 2H), 7.50 – 7.39 (m, 5H), 7.30 (d, J = 7.8 Hz, 2H), 7.25 – 7.17 (m, 2H), 7.17 – 7.10 (m, 1H), 3.49 (s, 3H), 2.43 (s, 3H) ppm.

^{13}C NMR (101 MHz, $CDCl_3$): δ = 148.2 (C_q), 138.8 (C_q), 137.8 (C_q), 134.9 (C_q), 131.5 (C_q), 131.0 (CH), 130.4 (C_q), 129.4 (CH), 129.1 (CH), 129.1 (CH), 128.6 (CH), 128.3 (C_q), 128.2 (CH), 127.1 (CH), 126.4 (CH), 33.3 (CH_3), 21.5 (CH_3) ppm.

IR (ATR): $\tilde{\nu}$ = 1601, 1503, 1484, 960, 825, 776, 731, 698, 512, 413 cm^{-1}.

MS (ESI) *m*/*z* (relative intensity): 671 (55) [2M+Na]$^+$, 347 (30) [M+Na]$^+$, 325 (100) [M+H]$^+$.
HRMS (ESI): *m*/*z* calcd. for $C_{23}H_{20}N_2Na^+$ [M+Na]$^+$ 347.1519, found 347.1522.
The spectral data are in accordance with those reported in the literature.[24]

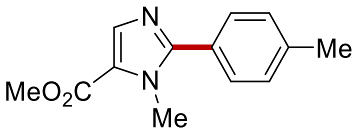

Methyl 1-methyl-2-(*p*-tolyl)-1*H*-imidazole-5-carboxylate (5h)

Following a modification of general procedure B, the title compound was obtained by using methyl 1-methyl-1*H*-imidazole-5-carboxylate (35.0 mg, 0.25 mmol), 4-iodotoluene (218 mg, 1.00 mmol), $[Cu(MeCN)_4]PF_6$ (28.0 mg, 75 µmol), 10-phenylphenothiazine (PTH) (3.4 mg, 12.5 µmol) and K_3PO_4 (159 mg, 0.75 mmol, 3.0 equiv.) in MeCN/THF (2:1, 0.5 mL). After stirring for 48 h under 390 nm irradiation, the product **5h** was obtained as colorless solid (28.8 mg, 50%) through the purification by column chromatography on silica gel (hexane/EtOAc 5:1).

M.p.: 112 °C.

^{1}H NMR (400 MHz, $CDCl_3$): δ = 7.82 (s, 1H), 7.51 (d, *J* = 8.1 Hz, 2H), 7.29 (d, *J* = 8.1 Hz, 2H), 3.94 (s, 3H), 3.87 (s, 3H), 2.42 (s, 3H) ppm.

^{13}C NMR (101 MHz, $CDCl_3$): δ = 161.3 (C_q), 153.0 (C_q), 139.9 (C_q), 137.4 (CH), 129.5 (CH), 129.3 (CH), 126.9 (C_q), 123.9 (C_q), 51.6 (CH_3), 34.3 (CH_3), 21.5 (CH_3) ppm.

IR (ATR): $\tilde{\nu}$ = 2949, 1702, 1519, 1449, 1359, 1298, 1218, 1111, 826, 506 cm^{-1}.

MS (ESI) *m*/*z* (relative intensity): 483 (100) [2M+Na]$^+$, 253 (47) [M+Na]$^+$, 231 (92) [M+H]$^+$.

HRMS (ESI): *m*/*z* calcd. for $C_{13}H_{15}N_2O_2^+$ [M+H]$^+$ 231.1128, found 231.1139.

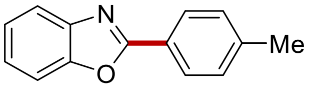

2-(*p*-Tolyl)benzo[*d*]oxazole (5i)

The general procedure B was followed using benzoxazole (29.8 mg, 0.25 mmol) and 4-iodotoluene (164 mg, 0.75 mmol). Purification by column chromatography on silica gel (hexane/EtOAc 25:1) yielded **5i** (41.3 mg, 79%) as yellow solid.

M.p.: 116 °C.

^{1}H NMR (400 MHz, $CDCl_3$): δ = 8.15 (d, *J* = 8.2 Hz, 2H), 7.79 – 7.74 (m, 1H), 7.59 – 7.54 (m, 1H), 7.38 – 7.29 (m, 4H), 2.44 (s, 3H) ppm.

^{13}C NMR (101 MHz, $CDCl_3$): δ = 163.4 (C_q), 150.8 (C_q), 142.3 (C_q), 142.2 (C_q), 129.8 (CH), 127.7 (CH), 125.0 (CH), 124.6 (CH), 124.5 (C_q), 120.0 (CH), 110.6 (CH), 21.8 (CH_3) ppm.

IR (ATR): $\tilde{\nu}$ = 1622, 1556, 1502, 1452, 1243, 1178, 1056, 821, 745, 502 cm^{-1}.

MS (ESI) *m*/*z* (relative intensity): 210 (100) $[M+H]^+$.

HRMS (ESI): *m*/*z* calcd. for $C_{14}H_{12}NO^+$ $[M+H]^+$ 210.0913, found 210.0920.

The spectral data are in accordance with those reported in the literature.[25]

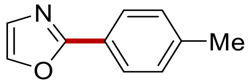

2-(*p*-Tolyl)oxazole (5j)

The general procedure B was followed using oxazole (17.3 mg, 0.25 mmol) and 4-iodotoluene (164 mg, 0.75 mmol). Purification by column chromatography on silica gel (hexane/EtOAc 20:1) yielded **5j** (22.7 mg, 57%) as yellow oil.

^{1}H NMR (400 MHz, $CDCl_3$): δ = 7.94 (d, *J* = 8.2 Hz, 2H), 7.68 (s, 1H), 7.26 (d, *J* = 8.0 Hz, 2H), 7.21 (s, 1H), 2.40 (s, 3H) ppm.

^{13}C NMR (101 MHz, $CDCl_3$): δ = 162.3 (C_q), 140.8 (C_q), 138.4 (CH), 129.6 (CH), 128.4 (CH), 126.5 (CH), 125.0 (C_q), 21.6 (CH_3) ppm.

IR (ATR): $\tilde{\nu}$ = 1613, 1494, 1362, 1261, 1139, 1103, 919, 824, 730, 504 cm^{-1}.

MS (ESI) *m*/*z* (relative intensity): 160 (100) $[M+H]^+$.

HRMS (ESI): *m*/*z* calcd. for $C_{10}H_{10}NO^+$ $[M+H]^+$ 160.0757, found 160.0760.

The spectral data are in accordance with those reported in the literature.[26]

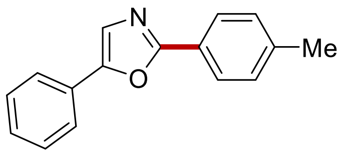

5-Phenyl-2-(*p*-tolyl)oxazole (5k)

The general procedure B was followed using 5-phenyloxazole (36.3 mg, 0.25 mmol) and 4-iodotoluene (164 mg, 0.75 mmol). Purification by column chromatography on silica gel (hexane/EtOAc 10:1) yielded **5k** (22.7 mg, 57%) as yellow solid.

M.p.: 66 °C.

^{1}H NMR (400 MHz, $CDCl_3$): δ = 8.01 (d, *J* = 8.3 Hz, 2H), 7.75 – 7.68 (m, 2H), 7.48 – 7.40 (m, 3H), 7.36 – 7.31 (m, 1H), 7.31 – 7.27 (m, 2H), 2.42 (s, 3H) ppm.

^{13}C NMR (101 MHz, $CDCl_3$): δ = 161.5 (C_q), 151.0 (C_q), 140.7 (C_q), 129.6 (CH), 129.0 (CH), 128.4 (CH), 128.2 (C_q), 126.4 (CH), 124.9 (C_q), 124.2 (CH), 123.5 (CH), 21.6 (CH_3) ppm.

IR (ATR): $\tilde{\nu}$ = 1612, 1497, 1134, 1020, 952, 824, 761, 730, 689, 486 cm^{-1}.

MS (ESI) *m*/*z* (relative intensity): 493 (50) $[2M+Na]^+$, 258 (100) $[M+Na]^+$, 236 (72) $[M+H]^+$.

HRMS (ESI): *m*/*z* calcd. for $C_{16}H_{13}NONa^+$ $[M+Na]^+$ 258.0889, found 258.0893.

The spectral data is in accordance with those reported in the literature.[27]

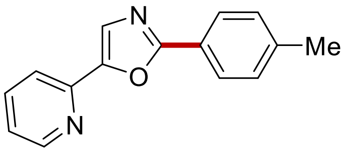

5-(Pyridin-2-yl)-2-(*p*-tolyl)oxazole (5l)

Following a modification of general procedure B, the title compound was obtained by using 5-(pyridin-2-yl)oxazole (36.5 mg, 0.25 mmol), 4-iodotoluene (218 mg, 1.00 mmol), $[Cu(MeCN)_4]PF_6$ (28.0 mg, 75 μmol), 10-phenylphenothiazine (PTH) (3.4 mg, 12.5 μmol) and K_3PO_4 (159 mg, 0.75 mmol, 3.0 equiv.) in MeCN/THF (2:1, 0.5 mL). After stirring for 48 h under 390 nm irradiation, the product **5l** was obtained as yellow solid (39.0 mg, 66%) through purification by column chromatography on silica gel (hexane/EtOAc 10:1).

M.p.: 114 °C.

^{1}H NMR (400 MHz, $CDCl_3$): δ = 8.64 (d, *J* = 4.8 Hz, 1H), 8.03 (d, *J* = 7.9 Hz, 2H), 7.84 – 7.70 (m, 3H), 7.29 (d, *J* = 8.0 Hz, 2H), 7.22 (dd, *J* = 7.1, 4.9 Hz, 1H), 2.42 (s, 3H) ppm.

^{13}C NMR (101 MHz, $CDCl_3$): δ = 162.4 (C_q), 150.6 (C_q), 150.1 (CH), 147.6 (C_q), 141.2 (C_q), 137.0 (CH), 129.7 (CH), 127.0 (CH), 126.7 (CH), 124.7 (C_q), 122.8 (CH), 119.2 (CH), 21.7 (CH_3) ppm.

IR (ATR): $\tilde{\nu}$ = 1604, 1576, 1491, 1468, 1425, 1133, 955, 822, 777, 730 cm^{-1}.

MS (ESI) *m*/*z* (relative intensity): 495 (42) $[2M+Na]^+$, 259 (100) $[M+Na]^+$, 237 (30) $[M+H]^+$.

HRMS (ESI): *m*/*z* calcd. for $C_{15}H_{12}N_2ONa^+$ $[M+Na]^+$ 259.0842, found 259.0849.

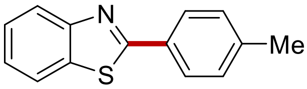

2-(*p*-Tolyl)benzo[*d*]thiazole (5m)

The general procedure B was followed using benzothiazole (33.8 mg, 0.25 mmol) and 4-iodotoluene (164 mg, 0.75 mmol). Purification by column chromatography on silica gel (hexane/EtOAc 20:1) yielded **5m** (34.9 mg, 62%) as brown solid.

M.p.: 76 °C.

^{1}H NMR (400 MHz, $CDCl_3$): δ = 8.07 (d, J = 8.1 Hz, 1H), 8.00 (d, J = 8.1 Hz, 2H), 7.89 (d, J = 8.0 Hz, 1H), 7.49 (ddd, J = 8.3, 7.2, 1.3 Hz, 1H), 7.37 (ddd, J = 7.6, 7.2, 1.2 Hz, 1H), 7.30 (d, J = 8.0, 7.6, 1.3 Hz, 2H), 2.43 (s, 3H) ppm.

^{13}C NMR (101 MHz, $CDCl_3$): δ = 168.4 (C_q), 154.3 (C_q), 141.6 (C_q), 135.1 (C_q), 131.1 (C_q), 129.9 (CH), 127.6 (CH), 126.4 (CH), 125.1 (CH), 123.2 (CH), 121.7 (CH), 21.7 (CH_3) ppm.

IR (ATR): $\tilde{\nu}$ = 3023, 1610, 1483, 1434, 1313, 1229, 962, 818, 759, 729 cm^{-1}.

MS (ESI) *m*/*z* (relative intensity): 248 (7) $[M+Na]^+$, 226 (100) $[M+H]^+$.

HRMS (ESI): *m*/*z* calcd. for $C_{14}H_{12}NS^+$ $[M+H]^+$ 226.0685, found 226.0687.

The spectral data is in accordance with those reported in the literature.[15]

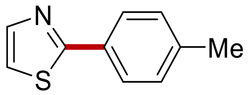

2-(*p*-Tolyl)thiazole (5n)

The general procedure B was followed using benzothiazole (21.3 mg, 0.25 mmol) and 4-iodotoluene (164 mg, 0.75 mmol). Purification by column chromatography on silica gel (hexane/EtOAc 30:1) yielded **5n** (25.0 mg, 57%) as colorless oil.

^{1}H NMR (400 MHz, $CDCl_3$): δ = 7.87 (d, J = 8.2 Hz, 2H), 7.85 (d, J = 3.3 Hz, 1H), 7.29 (d, J = 3.3 Hz, 1H), 7.26 (d, J = 8.1 Hz, 2H), 2.40 (s, 3H) ppm.

^{13}C NMR (101 MHz, $CDCl_3$): δ = 168.8 (C_q), 143.7 (CH), 140.4 (C_q), 131.1 (C_q), 129.8 (CH), 126.6 (CH), 118.5 (CH), 21.5 (CH_3) ppm.

IR (ATR): $\tilde{\nu}$ = 2920, 1484, 1143, 1056, 973, 817, 723, 710, 632, 480 cm^{-1}.

MS (ESI) *m*/*z* (relative intensity): 198 (23) $[M+Na]^+$, 176 (100) $[M+H]^+$.

HRMS (ESI): *m*/*z* calcd. for $C_{10}H_{10}NS^+$ $[M+H]^+$ 176.0528, found 176.0534.

The spectral data are in accordance with those reported in the literature.[28]

7 Gram scale synthesis

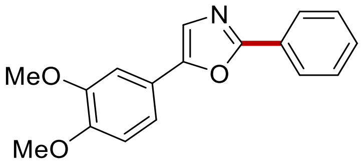

5-(3,4-Dimethoxyphenyl)-2-phenyloxazole (5o)

5-(3,4-Dimethoxyphenyl)oxazole (1.03 g, 5.0 mmol, 1.0 equiv.), CuI (190 mg, 1.0 mmol, 20 mol%), PTH (68 mg, 0.25 mmol, 5.0 mol%) and K_3PO_4 (3.18 g, 15 mmol, 3.0 equiv.) were filled in a 100 mL round-bottom Schlenk-flask equipped with a teflon-coated magnetic stir bar. After evacuating and backfilling with N_2 for three times, iodobenzene (3.06 g, 15 mmol, 3.0 equiv.) was added before the addition of MeCN (13.3 mL) and THF (6.7 mL). The resulting mixture was stirred for 18 h under violet LED irradiation (390 nm) using the standard experimental setup for photoreactions. Afterwards, the crude reaction mixture was filtered through a short plug of silica which was washed with EtOAc (250 mL). The solvent was removed under reduced pressure. Purification of the residue by column chromatography (hexane/EtOAc 3:1) yielded balsoxin (**5o**) (1.01 g, 72%) as yellow solid.

M.p.: 96 °C.

^{1}H NMR (400 MHz, $CDCl_3$): δ = 8.13 – 8.05 (m, 2H), 7.53 – 7.40 (m, 3H), 7.34 (s, 1H), 7.31 (d, J = 8.2 Hz, 1H), 7.19 (d, J = 2.2 Hz, 1H), 6.94 (d, J = 8.3 Hz, 1H), 3.98 (s, 3H), 3.93 (s, 3H) ppm.

^{13}C NMR (101 MHz, $CDCl_3$): δ = 160.8 (C_q), 151.4 (C_q), 149.6 (C_q), 149.5 (C_q), 130.3 (CH), 128.9 (CH), 127.7 (C_q), 126.3 (CH), 122.4 (CH), 121.2 (C_q), 117.4 (CH), 111.6 (CH), 107.6 (CH), 56.2 (CH_3), 56.1 (CH_3) ppm.

IR (ATR): $\tilde{\nu}$ = 2937, 1594, 1505, 1253, 1221, 1142, 1025, 856, 805, 710 cm^{-1}.

MS (ESI) *m*/*z* (relative intensity): 304 (95) $[M+Na]^+$, 282 (100) $[M+H]^+$.

HRMS (ESI): *m*/*z* calcd. for $C_{17}H_{16}NO_3^+$ $[M+H]^+$ 282.1125, found 282.1125.

The spectral data are in accordance with those reported in the literature.[9]

8 Ligand optimization atroposelective synthesis of benzimidazole 7

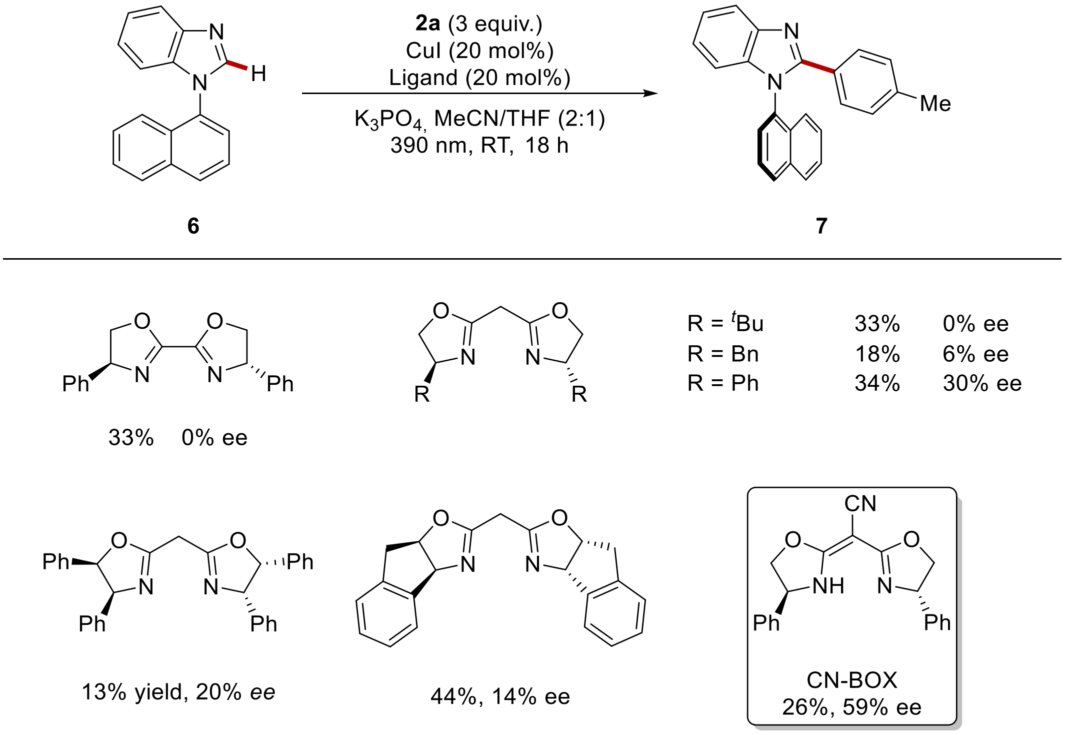

Scheme S1. Ligand optimization for the atroposelective, copper-catalyzed arylation.

Experimental Procedure using CN-BOX ligand

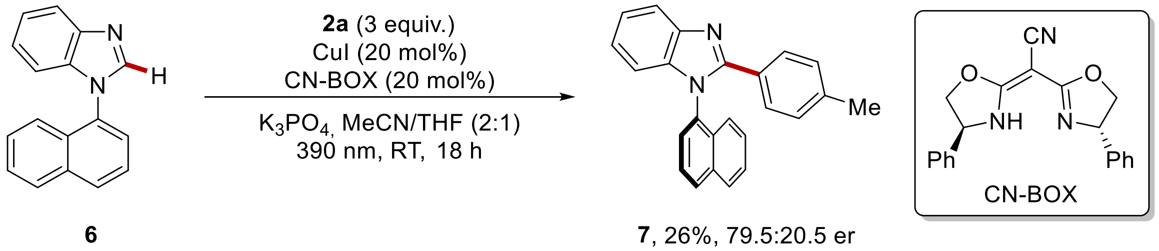

In a glovebox, CuI (3.8 mg, 20 µmol, 20 mol%) and CN-BOX (6.6 mg, 20 µmol, 20 mol%) were filled in an oven-dried 10 mL vial and MeCN (0.5 mL) and THF (0.25 mL) were added subsequently. The resulting mixture was stirred for 30 min at ambient temperature before 1-(naphthalen-1-yl)-1*H*-benzo[*d*]imidazole (24.4 mg, 0.1 mmol, 1.0 equiv.), 4-iodotoluene (65 mg, 0.3 mmol, 3.0 equiv.) and K_3PO_4 (64 mg, 0.3 mmol, 3.0 equiv.) were added. The vial was sealed with a rubber septum and wrapped with parafilm. The vial was transferred out of the glovebox and the reaction mixture was stirred at ambient temperature (fan cooling) under violet LED irradiation (390 nm) for 18 h. Afterwards, the crude reaction mixture was filtered through a short plug of silica which was washed with EtOAc (20 mL). The solvent was removed under reduced pressure. Purification of the residue by column chromatography

(hexane/EtOAc 5:1) yielded 1-(naphthalen-1-yl)-2-(*p*-tolyl)-1*H*-benzo[*d*]imidazole (**7**) (8.7 mg, 26%) as yellow solid.

M.p.: 163 °C.

^{1}H NMR (400 MHz, $CDCl_3$): δ = 8.01 (td, J = 8.5, 1.1 Hz, 2H), 7.95 (dt, J = 8.2, 1.0 Hz, 1H), 7.60 – 7.53 (m, 2H), 7.45 – 7.37 (m, 5H), 7.34 (ddd, J = 8.2, 7.1, 1.1 Hz, 1H), 7.16 (ddd, J = 8.3, 7.1, 1.1 Hz, 1H), 6.97 (d, J = 8.1 Hz, 2H), 6.85 (dd, J = 8.1, 1.0 Hz, 1H), 2.25 (s, 3H).

^{13}C NMR (101 MHz, $CDCl_3$): δ = 153.6 (C_q), 143.1 (C_q), 139.7 (C_q), 138.4 (C_q), 134.6 (C_q), 133.9 (C_q), 130.4 (C_q), 129.7 (CH), 129.2 (CH), 128.8 (CH), 128.6 (CH), 127.8 (CH), 127.2 (C_q), 127.1 (CH), 126.7 (CH), 125.8 (CH), 123.3 (CH), 123.1 (CH), 123.0 (CH), 119.8 (CH), 111.0 (CH), 21.4 (CH_3) ppm.

IR (ATR): $\tilde{\nu}$ = 1597, 1450, 1411, 1367, 1318, 1265, 825, 802, 774, 744 cm^{-1}.

MS (ESI) *m*/*z* (relative intensity): 691 (40) $[2M+Na]^+$, 669 (15) $[2M+H]^+$, 357 $[M+H]^+$ (15), 335 (100) $[M+H]^+$.

HRMS (ESI): *m*/*z* calcd. for $C_{24}H_{19}N_2^+$ $[M+H]^+$ 335.1548, found 335.1543.

R_t (IA-3 column, *n*-hexane/*i*-PrOH 90/10, 1.0 mL/min, 250.4 nm): tr(major) = 9.58 min, tr(minor) = 17.54 min, 79.5:20.5 er

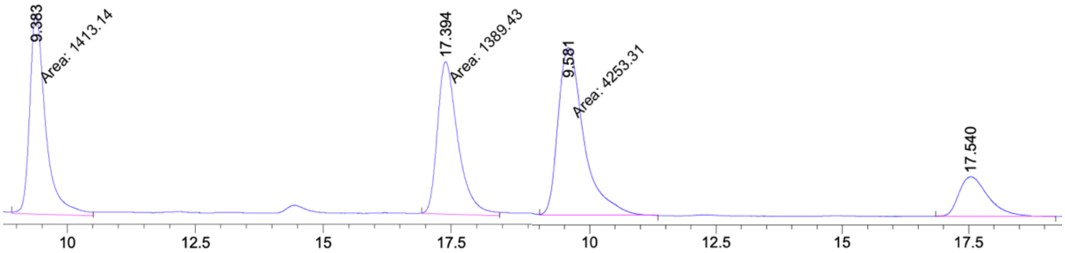

Peak #	RetTime [min]	Type	Width [min]	Area [mAU*s]	Height [mAU]	Area %
1	9.579	BB	0.5440	2991.61133	83.50253	79.3887
2	17.543	BB	0.4835	776.69781	19.34105	20.6113

Peak #	RetTime [min]	Type	Width [min]	Area [mAU*s]	Height [mAU]	Area %
1	9.383	MM	0.3452	912.23560	44.04128	50.0324
2	17.394	MM	0.4502	911.05365	33.73127	49.9676

9 Mechanistic experiments

9.1 Intermolecular competition experiment

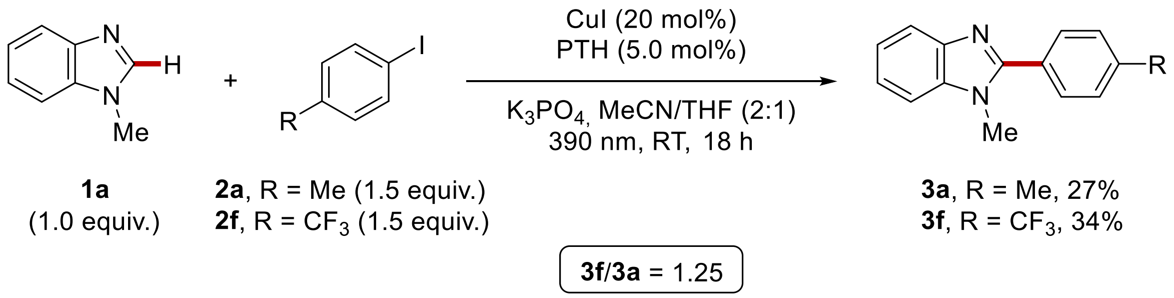

1-Methylbenzimidazole (**1a**) (33.0 mg, 0.25 mmol), 4-iodotoluene (**2a**) (81.8 mg, 375 µmol), 1-iodo-4-(trifluoromethyl)benzene (**2f**) (102 mg, 375 µmol), CuI (9.5 mg, 50.0 µmol, 20 mol%), PTH (3.4 mg, 12.5 µmol, 5.0 mol%) and K_3PO_4 (159 mg, 0.75 mmol, 3.0 equiv.) were filled in a 10 mL vial equipped with a teflon-coated stir bar. The vial was sealed with a rubber septum and was evacuated and purged with N_2 three times before MeCN (0.67 mL) and THF (0.33 mL) were added. Afterwards, the vial was wrapped with parafilm. The resulting solution was stirred under violet light irradiation (2 × Kessil PR160L, internal temperature was determined to be 35 °C). After 18 h, the resulting mixture was filtered through a short pad of silica gel and washed with EtOAc and the solvent was removed under reduced pressure. The crude mixture was analyzed by ^{1}H NMR spectroscopy using 1,3,5-trimethoxybenzene as the internal standard affording a product ratio **3f**/**3a** = 1.25.

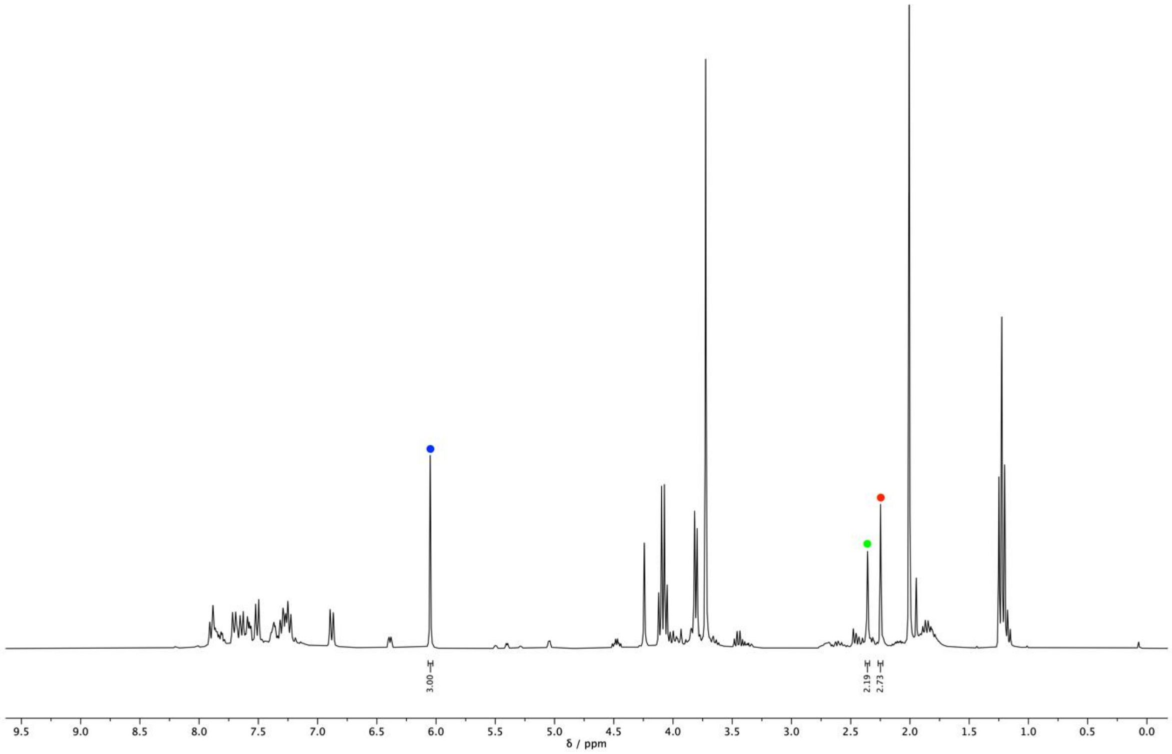

Figure S3. ^{1}H NMR spectrum of competition experiment between **2a** and **2f** with benzimidazole **1a**. Blue dot = 1,3,5-trimethoxybenzene (internal standard), green dot = **3a** (Me), red dot = **3f** (CF_3).

9.2 Kinetic isotope effect

The kinetic isotope effect (KIE) was determined running two independent reactions in parallel with either 1-methylbenzimidazole (**1a**) or 1-methyl-1*H*-benzo[*d*]imidazole-2-*d* ([D]$_1$-**1a**).

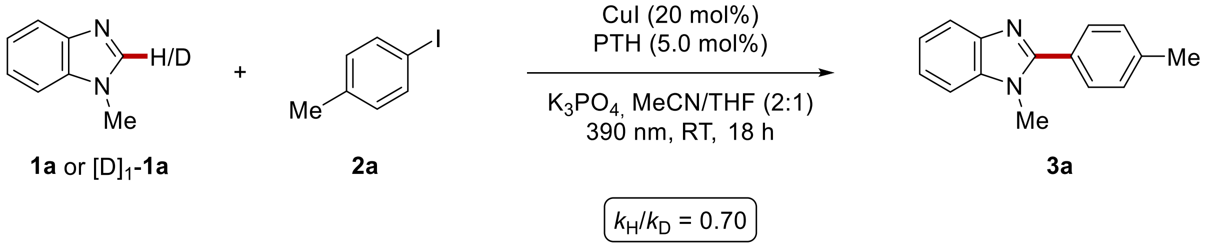

Following general procedure B, the reactions were set up using 1-methylbenzimidazole (**1a**) 66.1 mg, 0.50 mmol, 1.0 equiv.) or 2-deutero-1-methylbenzimidazole ([D]$_1$-**1a**) (66.6 mg, 0.50 mmol, 1.0 equiv.), 4-iodotoluene (327 mg, 1.50 mmol, 3.00 equiv.), CuI (19.0 mg, 0.10 mmol, 20 mol%), PTH (6.9 mg, 25.0 µmol, 5.0 mol%), K_3PO_4 (318 mg, 1.50 mmol,

3.0 equiv.) and 1,3,5-trimethoxybenzene (18.0 mg, 10.7 mmol) in MeCN (1.33 mL) and THF (0.67 mL). The reactions were stirred under 390 nm irradiation. Aliquots of 20 µL were taken after 20, 40, 60, 80, 100, 120, 140, 160, 180, 200 and 220 min and filtered through a short plug of silica gel. The silica plug was flushed with EtOAc and the resulting solution was analyzed by GC.

A linear regression was applied for the reaction in the interval 100 to 180 min. The quotient of the obtained slopes gave the KIE value.

Table S7. Yield *versus* time profile for **1a** and $[D]_1$-**1a**.

***t* (min)**	**H (1st run)**	**H (2nd run)**	**D (1st run)**	**D (2nd run)**
20	0.6	0.6	0.6	0.6
40	1.2	1.1	1.2	1.2
60	2.0	2.0	2.2	2.1
80	3.8	3.6	3.8	3.9
100	6.6	6.2	7.5	6.8
120	11.2	9.6	13.0	11.4
140	16.5	15.1	19.8	18.9
160	20.3	21.0	28.0	28.3
180	28.1	26.0	35.8	36.1
200	28.7	30.5	41.3	40.3
220	30.7	34.0	44.7	43.0
Rate	0.26025	0.25539	0.35821	0.37785
R^2 (linear fit)	0.9860	0.9936	0.9944	0.9879

$$\mathrm{KIE} = k_H / k_D$$

$$\mathrm{KIE}_1 = 0.26025/0.3821 = 0.7265$$

$$\mathrm{KIE}_2 = 0.25539/0.37785 = 0.6759$$

Overall KIE = 0.70

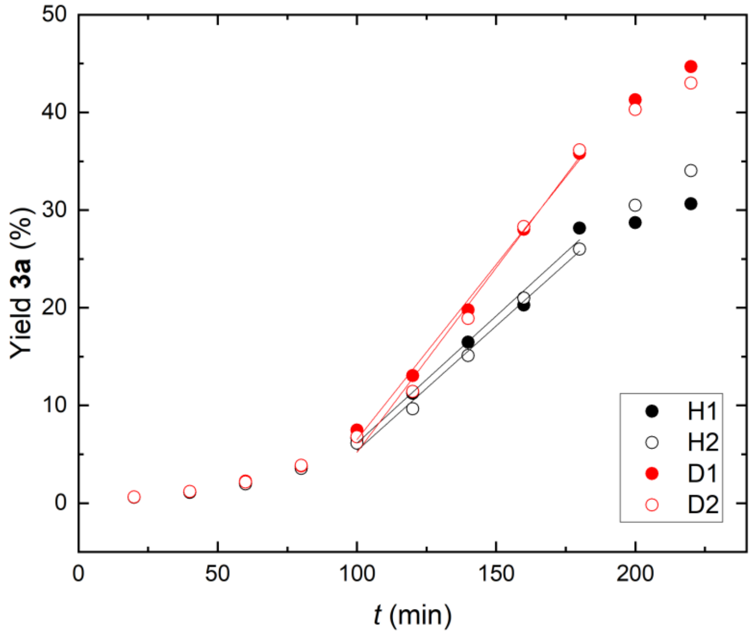

Figure S4. Conversion *versus* time profile of **3a** for **1a** and [D]$_1$-**1a**.

9.3 Radical scavenger experiments

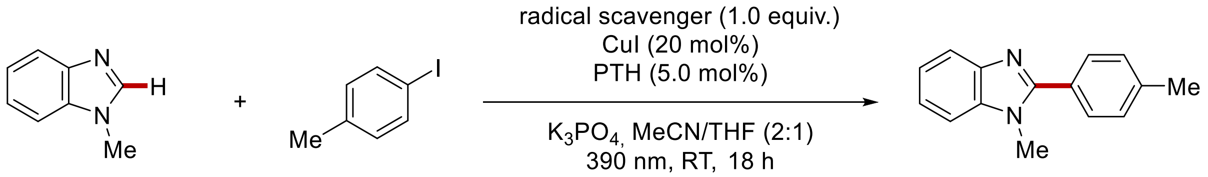

To an oven-dried 10 mL vial with a teflon-coated stir bar 1-methylbenzimidazole (33.0 mg, 0.25 mmol, 1.0 equiv.), 4-iodotoluene (164 mg, 0.75 mmol, 3.0 equiv.), CuI (50.0 µmol, 20 mol%), PTH (3.4 mg, 12.5 µmol, 5.0 mol%), K_3PO_4 (159 mg, 0.75 mmol, 3.0 equiv.) and the radical scavenger (1.0 equiv.) were added. The vial was sealed with a rubber septum and evacuated and purged with N_2 three times before the addition of MeCN (0.67 mL) and THF (0.33 mL). After wrapping the vial with parafilm, the resulting solution was stirred under violet light irradiation. After 18 h, the mixture was filtered through a pad of silica gel and washed with EtOAc. The filtrate was concentrated *in vacuo* and the crude reaction mixture was analyzed by HRMS. Further purification by column chromatography on silica gel (hexane/EtOAc 5:1) afforded product **3a**.

Table S8. Product yield of **3a** in radical scavenger experiment.

Entry	Radical scavenger (1.0 equiv.)	Yield (%)
1	---	77
2	TEMPO	12
3	Galvinoxyl	0
4	BHT	0

Yield of isolated products. TEMPO = 2,2,6,6-tetramethylpiperidinyloxyl. BHT = butylated hydroxytoluene.

a)

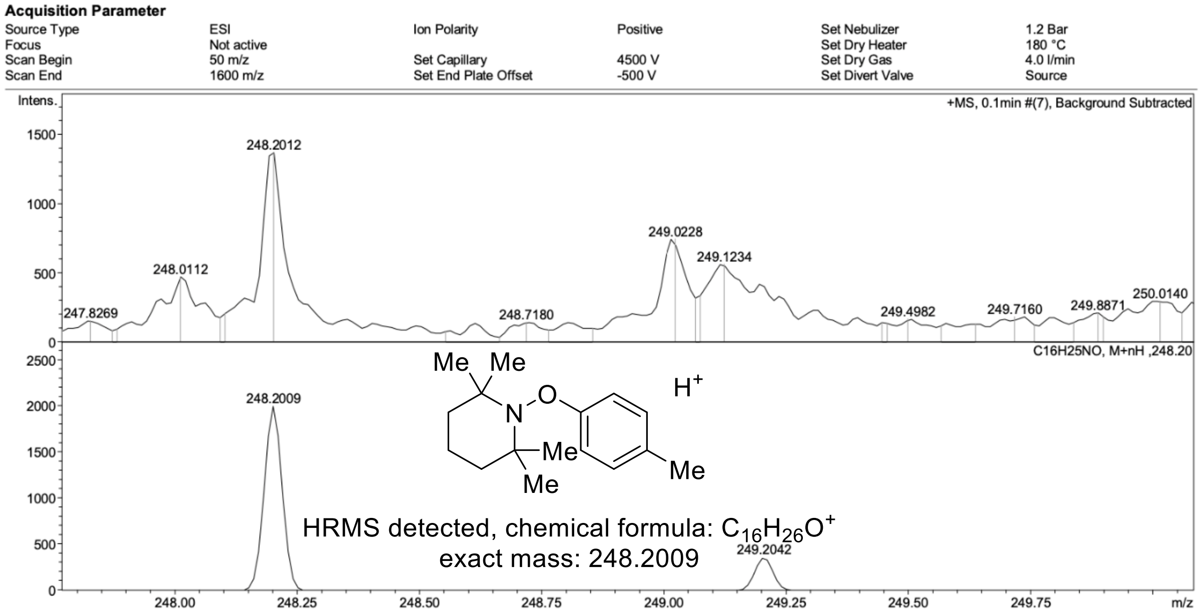

b)

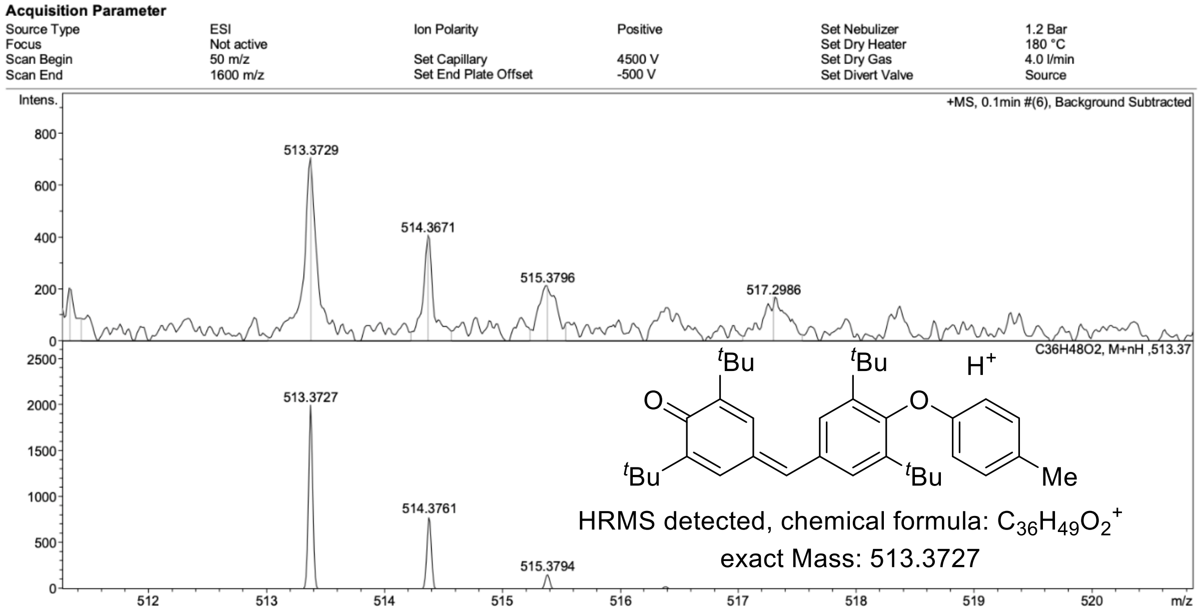

Figure S5. HRMS spectra of a) TEMPO-tolyl and b) galvinoxyl-tolyl adduct. Top: measured, bottom: simulated.

9.4 On/off experiment

A 10 mL glass vial was charged with 1-methylbenzimidazole (66.1 mg, 0.50 mmol, 1.0 equiv.) and 4-iodotoluene (327 mg, 1.5 mmol, 3.0 equiv.), CuI (19.0 mg, 0.10 mmol, 20 mol%), PTH (6.9 mg, 25.0 µmol, 5.0 mol %), K_3PO_4 (318 mg, 1.50 mmol, 3.0 equiv.) and 1,3,5-trimethoxybenzene (19.8 mg, 0.12 mmol) was added as internal standard. The vial was sealed with a rubber septum and wrapped with parafilm. The vial was evacuated and purged with N_2 three times before the addition of MeCN (1.33 mL) and THF (0.67 mL). Starting with lights turned on, aliquots of 20 µL were taken every 45 min. In the same interval, the light was turned on or off, respectively. The aliquots were diluted with $CDCl_3$, filtered through a short plug of silica gel and analyzed by ^{1}H NMR spectroscopy to determine the yield of product **3a**.

Table S9. Yield *versus* time profile for arylated benzimidazole **3a** in the on/off experiment.

**Entry**	**Light**	***t* (min)**	**Yield (%)**
1	On	45	2
2	Off	90	2
3	On	135	6
4	Off	180	6
5	On	225	15
6	Off	270	15
7	On	315	31

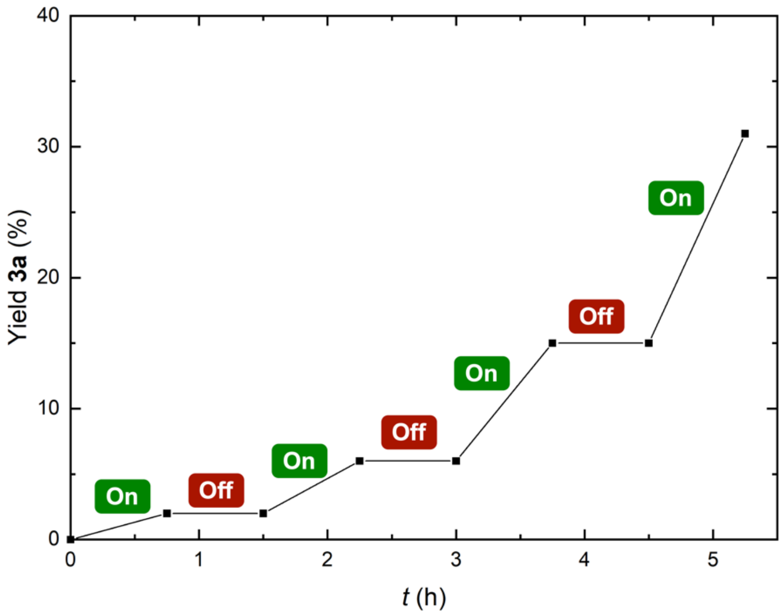

Figure S6. On/off experiment showing the influence of the visible light irradiation on the formation of **3a**.

9.5 Determination of quantum yield

The quantum yield measurements were conducted by following a literature procedure.[29] All preparations were carried out in the dark.

1) Preparation of ferrioxalate solution.

   In a volumetric flask, potassium ferrioxalate trihydrate (295 mg) and H_2SO_4 (140 µL) were diluted to finale volume of 50 mL H_2O.

2) Preparation of buffer solution.

   In a volumetric flask, NaOAc (2.48 g) and H_2SO_4 (0.5 mL) were diluted to a finale volume of 50 mL.

The quantum yield was determined by irradiating the ferrioxalate solution (2 mL) for 10 s with the standard reaction setup for 390 nm reactions. Subsequently, buffer solution (4 mL) containing 1,10-phenantroline (2 mg) was added and the resulting solution was diluted to a finale volume of 10 mL. The sample solution was then stored in the dark for 1 h to enable complete complex formation. Afterwards, the absorbance of this solution was measured using a quartz cuvette ($l = 1$ cm) at 510 nm (with $\epsilon = 11100\ \mathrm{M^{-1}cm^{-1}}$).[30] Following the same

procedure for a non-irradiated sample, the quantum yield was determined based on the different absorbances.

Calculation number of photons.

Absorbance A_0 of Fe^{2+} at 510 nm (non-irradiated sample): A_0 = 0.0256

Absorbance A of Fe^{2+} at 510 nm (irradiated sample): A = 4.0312 (after 10 s irradiation)

Difference Absorbance: $A - A_0 = 4.0312 - 0.0256 = 4.0056$

$$[\mathrm{Fe}^{2+}] = \frac{A - A_0}{\epsilon(\mathrm{Fe}^{2+}_{510\ \mathrm{nm}}) \cdot l} = \frac{4.0056}{11100\ \mathrm{M}^{-1}\mathrm{cm}^{-1} \cdot 1\ \mathrm{cm}} = 3.6086 \cdot 10^{-4}\ \mathrm{M}$$

$$n(\mathrm{Fe}^{2+}) = 3.6086 \cdot 10^{-4}\ \mathrm{M} \cdot 0.010\ \mathrm{L} = 3.6086 \cdot 10^{-6}\ \mathrm{mol}$$

With a quantum yield $\Phi = 1.14$ for the ferrioxalate actinometer at 405 nm[30] (*Annotation*: *It is assumed that the value for the quantum yield is also applicable for 390 nm irradiation*):

$$n(\mathrm{photons}) = \frac{n(\mathrm{Fe}^{2+})}{\Phi} = \frac{3.6086 \cdot 10^{-6}\ \mathrm{mol}}{1.14} = 3.1654 \cdot 10^{-6}\ \mathrm{mol}$$

$$n(\mathrm{photons/s}) = \frac{n(\mathrm{photons})}{t} = \frac{3.1654 \cdot 10^{-6}\ \mathrm{mol}}{10\ \mathrm{s}} = 3.1654 \cdot 10^{-7}\ \mathrm{mol\ s}^{-1}$$

With the overall product formation rate $n(\mathrm{product/s}) = 2.1485 \cdot 10^{-8}\ \mathrm{mol\ s}^{-1}$ obtained from the KIE experiment, the quantum yield is:

$$\boxed{\mathrm{Quantum\ Yield} = \frac{n(\mathrm{product/s})}{n(\mathrm{photons/s})} = \frac{2.1485 \cdot 10^{-8}\ \mathrm{mol\ s}^{-1}}{3.1654 \cdot 10^{-7}\ \mathrm{mol\ s}^{-1}} = 0.07 = 7\%}$$

9.6 Fluorescence quenching experiment

The fluorescence quenching experiment was performed by preparing sample solutions of PTH [PTH] = 2 mM and varying concentrations of 4-iodotoluene (**2a**) in MeCN/THF (2:1). Before the measurement, the sample solutions were degassed by sparging with N_2. Fluoresence spectra were determined with a fixed excitation wavelength of 390 nm. The Stern-Volmer plot was obtained by plotting the I_0/I ratio against the quencher concentration after determining the intensity of the fluorescence emission spectrum of PTH at 445 nm.

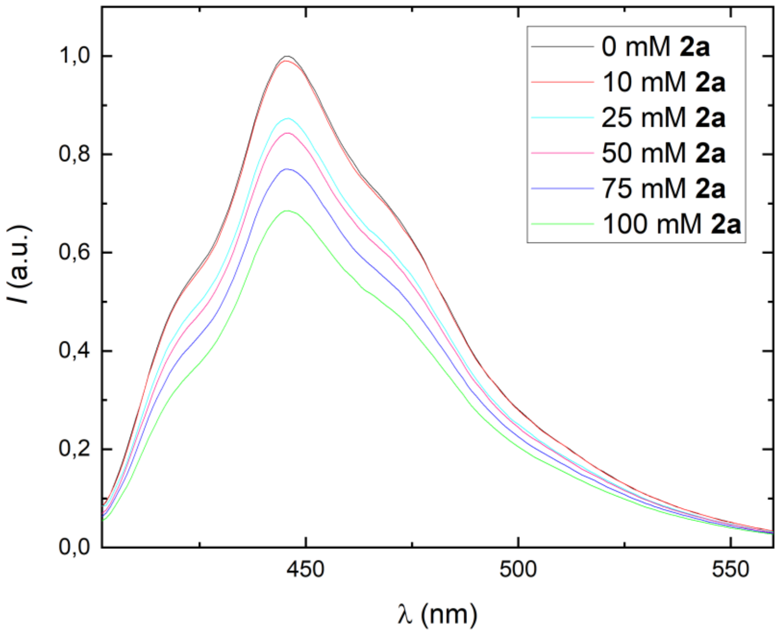

Figure S7. Fluorescence emission spectra of PTH with varying concentrations of **2a**.

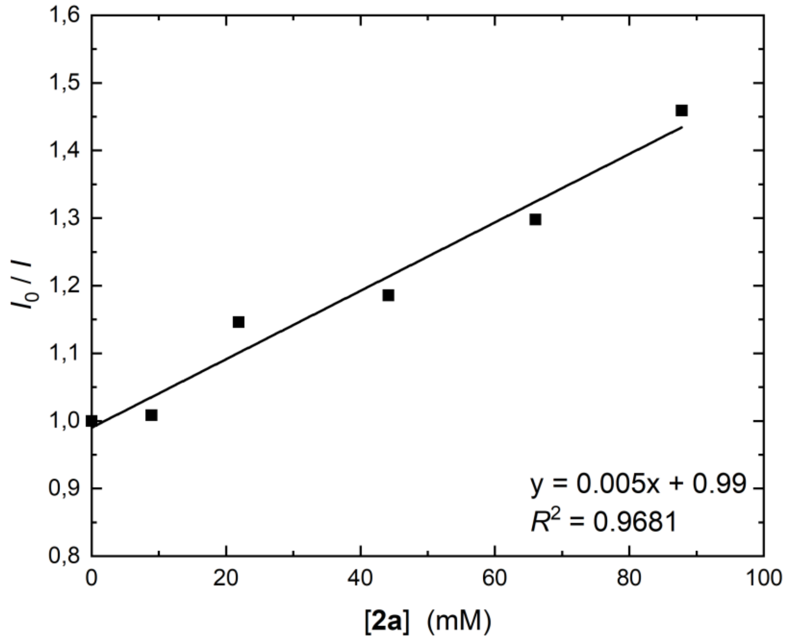

Figure S8. Stern-Volmer plot for PTH with increasing concentation of 4-iodotoluene.

10 References

[1] Y. Zhang, D. Jiang, Z. Fang, N. Zhu, N. Sun, W. He, C. Liu, L. Zhao, K. Guo, Photomediated core modification of organic photoredox catalysts in radical addition: mechanism and applications. *Chem. Sci.* **2021**, *12*, 9432–9441.

[2] Y. Dong, B. Breit, Cu-Catalyzed C–H Allylation of Benzimidazoles with Allenes. *Org. Lett.* **2021**, *23*, 6765–6769.

[3] R. C. Nishad, A. Rit, Self-Assembly of Benzimidazole-Derived Tris-NHC Ligands and AgI-Ions to Hexanuclear Organometallic Cages and Their Unusual Transmetalation Chemistry. *Chem. Eur. J.* **2021**, *27*, 594-599.

[4] C. M. Crudden, J. H. Horton, M. R. Narouz, Z. Li, C. A. Smith, K. Munro, C. J. Baddeley, C. R. Larrea, B. Drevniok, B. Thanabalasingam, A. B. McLean, O. V. Zenkina, I. I. Ebralidze, Z. She, H.-B. Kraatz, N. J. Mosey, L. N. Saunders, A. Yagi, Simple direct formation of self-assembled N-heterocyclic carbene monolayers on gold and their application in biosensing. *Nature Communications* **2016**, *7*, 12654.

[5] G.-L. Gao, C. Yang, W. Xia, Selective C–H trifluoromethylation of benzimidazoles through photoredox catalysis. *Chemical Communications* **2017**, *53*, 1041-1044.

[6] J.-Q. Chen, X. Liu, J. Guo, Z.-B. Dong, A Chemoselective and Desulfurative Chan–Lam Coupling: C–N Bond Formation between Benzimidazoline-2-Thiones and Arylboronic Acids. *Eur. J. Org. Chem.* **2020**, *2020*, 2414-2424.

[7] W.-C. Lee, C.-H. Wang, Y.-H. Lin, W.-C. Shih, T.-G. Ong, Tandem Isomerization and C–H Activation: Regioselective Hydroheteroarylation of Allylarenes. *Org. Lett.* **2013**, *15*, 5358–5361.

[8] F. Bellina, S. Cauteruccio, A. Di Fiore, R. Rossi, Regioselective Synthesis of 4,5-Diaryl-1-methyl-1H-imidazoles Including Highly Cytotoxic Derivatives by Pd-Catalyzed Direct C-5 Arylation of 1-Methyl-1H-imidazole with Aryl Bromides. *Eur. J. Org. Chem.* **2008**, *2008*, 5436-5445.

[9] F. Besselièvre, F. Mahuteau-Betzer, D. S. Grierson, S. Piguel, Ligandless Microwave-Assisted Pd/Cu-Catalyzed Direct Arylation of Oxazoles. *J. Org. Chem.* **2008**, *73*, 3278–3280.

[10] C. Hardouin, M. J. Kelso, F. A. Romero, T. J. Rayl, D. Leung, I. Hwang, B. F. Cravatt, D. L. Boger, Structure−Activity Relationships of α-Ketooxazole Inhibitors of Fatty Acid Amide Hydrolase. *J. Med. Chem.* **2007**, *50*, 3359-3368.

[11] N. Wurzer, U. Klimczak, T. Babl, S. Fischer, R. A. Angnes, D. Kreutzer, A. Pattanaik, J. Rehbein, O. Reiser, Heck-Type Coupling of Fused Bicyclic Vinylcyclopropanes: Synthesis of 1,2-Dihydropyridines, 2,3-Dihydro-1H-azepines, 1,4-Cyclohexadienes, and 2H-Pyrans. *ACS Catal.* **2021**, *11*, 12019–12028.

[12] C.-H. Kung, C.-H. Kwon, Carbamate derivatives of felbamate as potential anticonvulsant agents. *Medicinal Chemistry Research* **2010**, *19*, 498-513.

[13] Z.-S. Gu, W.-X. Chen, L.-X. Shao, N-Heterocyclic Carbene-Palladium(II)-1-Methylimidazole Complex-Catalyzed Direct C–H Bond Arylation of (Benz)imidazoles with Aryl Chlorides. *J. Org. Chem.* **2014**, *79*, 5806–5811.

[14] X. Jin, Y. Liu, Q. Lu, D. Yang, J. Sun, S. Qin, J. Zhang, J. Shen, C. Chu, R. Liu, Formation of C✓N bonds by the release of H2: a new strategy for synthesis of imines and benzazoles. *Org. Biomol. Chem.* **2013**, *11*, 3776–3780.

[15] J. Huang, J. Chan, Y. Chen, C. J. Borths, K. D. Baucom, R. D. Larsen, M. M. Faul, A Highly Efficient Palladium/Copper Cocatalytic System for Direct Arylation of Heteroarenes: An Unexpected Effect of Cu(Xantphos)I. *J. Am. Chem. Soc.* **2010**, *132*, 3674–3675.

[16] W. Zhang, Q. Zeng, X. Zhang, Y. Tian, Y. Yue, Y. Guo, Z. Wang, Ligand-Free CuO Nanospindle Catalyzed Arylation of Heterocycle C–H Bonds. *J. Org. Chem.* **2011**, *76*, 4741–4745.

[17] J. C. Lewis, J. Y. Wu, R. G. Bergman, J. A. Ellman, Microwave-Promoted Rhodium-Catalyzed Arylation of Heterocycles through C□H Bond Activation. *Angew. Chem. Int. Ed.* **2006**, *45*, 1589–1591.

[18] T. B. Nguyen, L. Ermolenko, M. Corbin, A. Al-Mourabit, Fe/S-catalyzed decarboxylative redox condensation of arylacetic acids with nitroarenes. *Org. Chem. Front.* **2014**, *1*, 1157–1160.

[19] T.-H. Wang, R. Ambre, Q. Wang, W.-C. Lee, P.-C. Wang, Y. Liu, L. Zhao, T.-G. Ong, Nickel-Catalyzed Heteroarenes Cross Coupling via Tandem C–H/C–O Activation. *ACS Catal.* **2018**, *8*, 11368–11376.

[20] H.-B. Zhao, Z.-W. Hou, Z.-J. Liu, Z.-F. Zhou, J. Song, H.-C. Xu, Amidinyl Radical Formation through Anodic N−H Bond Cleavage and Its Application in Aromatic C−H Bond Functionalization. *Angew. Chem. Int. Ed.* **2017**, *56*, 587–590.

[21] H. Shi, T. Wu, P. Jiang, X. Jin, H. Zhu, Synthesis and Optical Properties of Novel Fluorescence-Traced Benzimidazolium Bromides. *J. Heterocycl. Chem.* **2014**, *51*, E71–E76.

[22] M. M. Guru, M. A. Ali, T. Punniyamurthy, Copper-Mediated Synthesis of Substituted 2-Aryl-N-benzylbenzimidazoles and 2-Arylbenzoxazoles via C–H Functionalization/C–N/C–O Bond Formation. *J. Org. Chem.* **2011**, *76*, 5295–5308.

[23] J. Jayabharathi, V. Thanikachalam, K. Jayamoorthy, Synthesis of some fluorescent benzimidazole derivatives using cobalt(ii) hydroxide as highly efficient catalyst – spectral and physico-chemical studies and ESIPT process. *Photochem. Photobiol. Sci.* **2013**, *12*, 1761–1773.

[24] N.-t. Zhang, C.-c. Zeng, C. M. Lam, R. K. Gbur, R. D. Little, Triarylimidazole Redox Catalysts: Electrochemical Analysis and Empirical Correlations. *J. Org. Chem.* **2013**, *78*, 2104–2110.

[25] Y. Kawashita, N. Nakamichi, H. Kawabata, M. Hayashi, Direct and Practical Synthesis of 2-Arylbenzoxazoles Promoted by Activated Carbon. *Org. Lett.* **2003**, *5*, 3713–3715.

[26] K. Lee, C. M. Counceller, J. P. Stambuli, Nickel-Catalyzed Synthesis of Oxazoles via C−S Activation. *Org. Lett.* **2009**, *11*, 1457–1459.

[27] H. Hachiya, K. Hirano, T. Satoh, M. Miura, Nickel-Catalyzed Direct C□H Arylation and Alkenylation of Heteroarenes with Organosilicon Reagents. *Angew. Chem. Int. Ed.* **2010**, *49*, 2202–2205.

[28] J. Jensen, N. Skjaerbaek, P. Vedsø, Preparation of 2- and 5-Aryl Substituted Thiazoles via Palladium-Catalyzed Negishi Cross-Coupling. *Synthesis* **2001**, 128–134.

[29] I. Choi, V. Müller, G. Lole, R. Köhler, V. Karius, W. Viöl, C. Jooss, L. Ackermann, Photoinduced Heterogeneous C−H Arylation by a Reusable Hybrid Copper Catalyst. *Chem. Eur. J.* **2020**, *26*, 3509–3514.

[30] H. J. Kuhn, S. E. Braslavsky, R. Schmidt, Chemical actinometry (IUPAC Technical Report). *Pure Appl. Chem.* **2004**, *76*, 2105–2146.

11 NMR Spectra

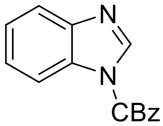

4e

^{1}H NMR (400 MHz, $CDCl_3$)

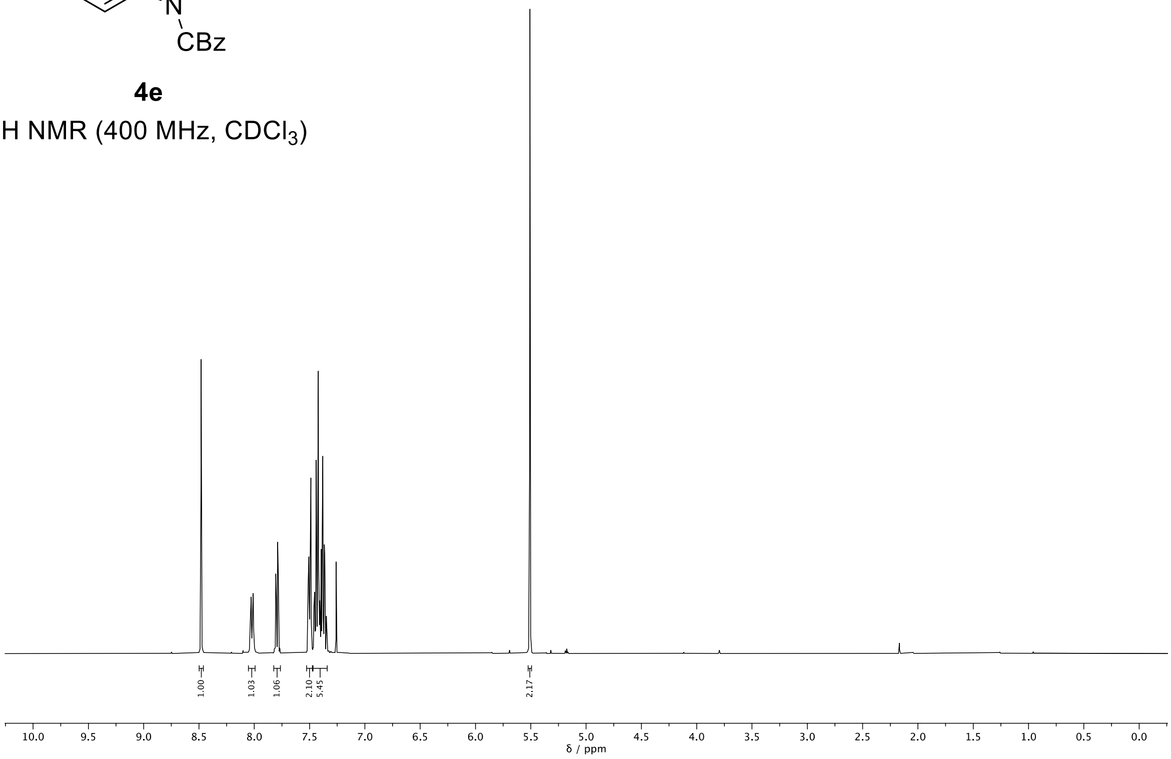

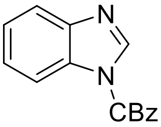

4e

^{13}C NMR (101 MHz, $CDCl_3$)

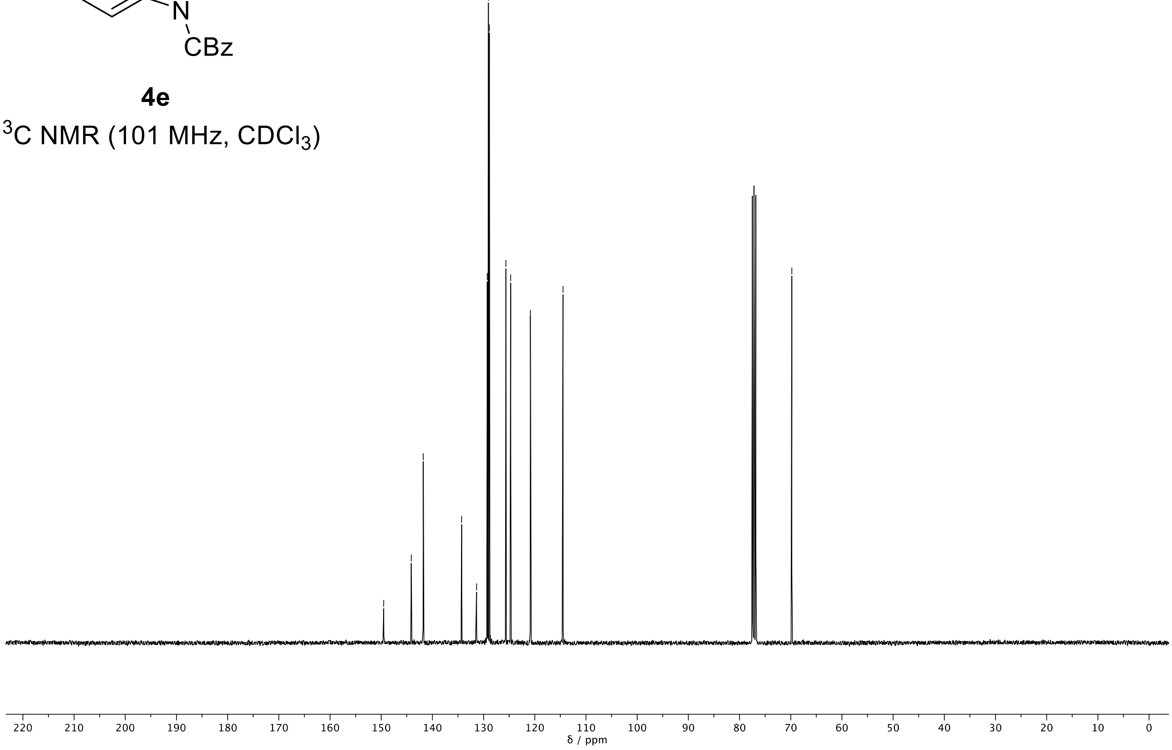

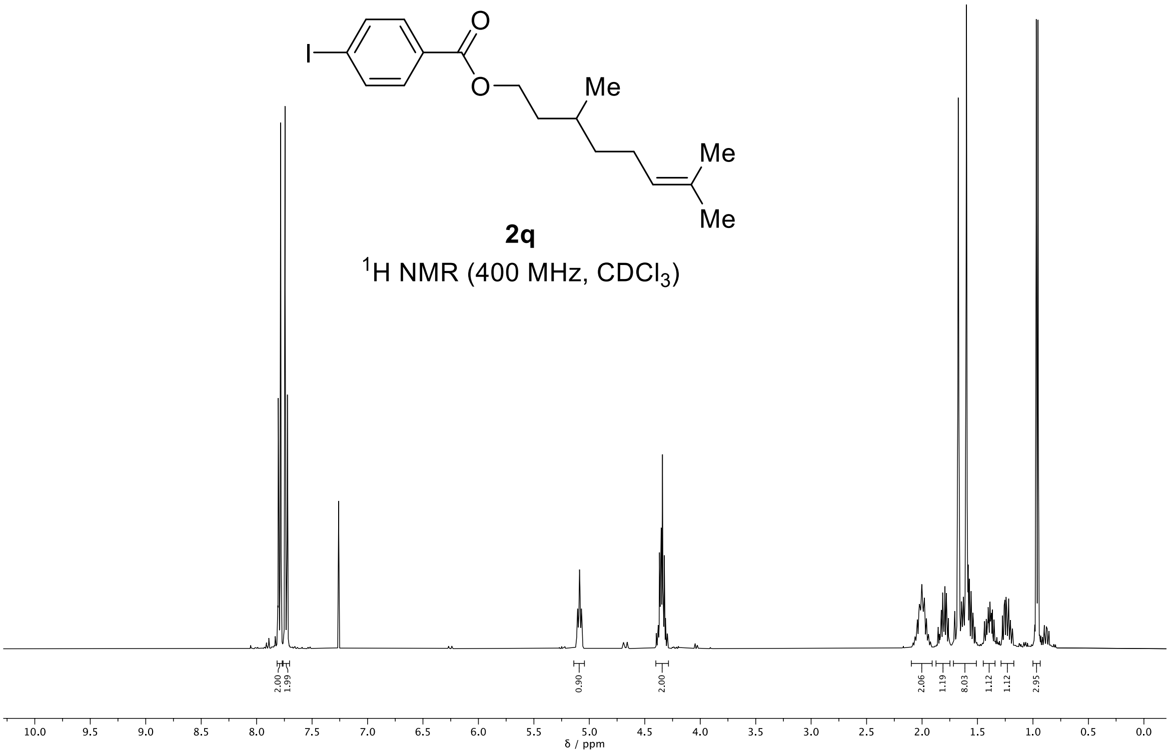
2q
^{1}H NMR (400 MHz, CDCl$_3$)
δ / ppm

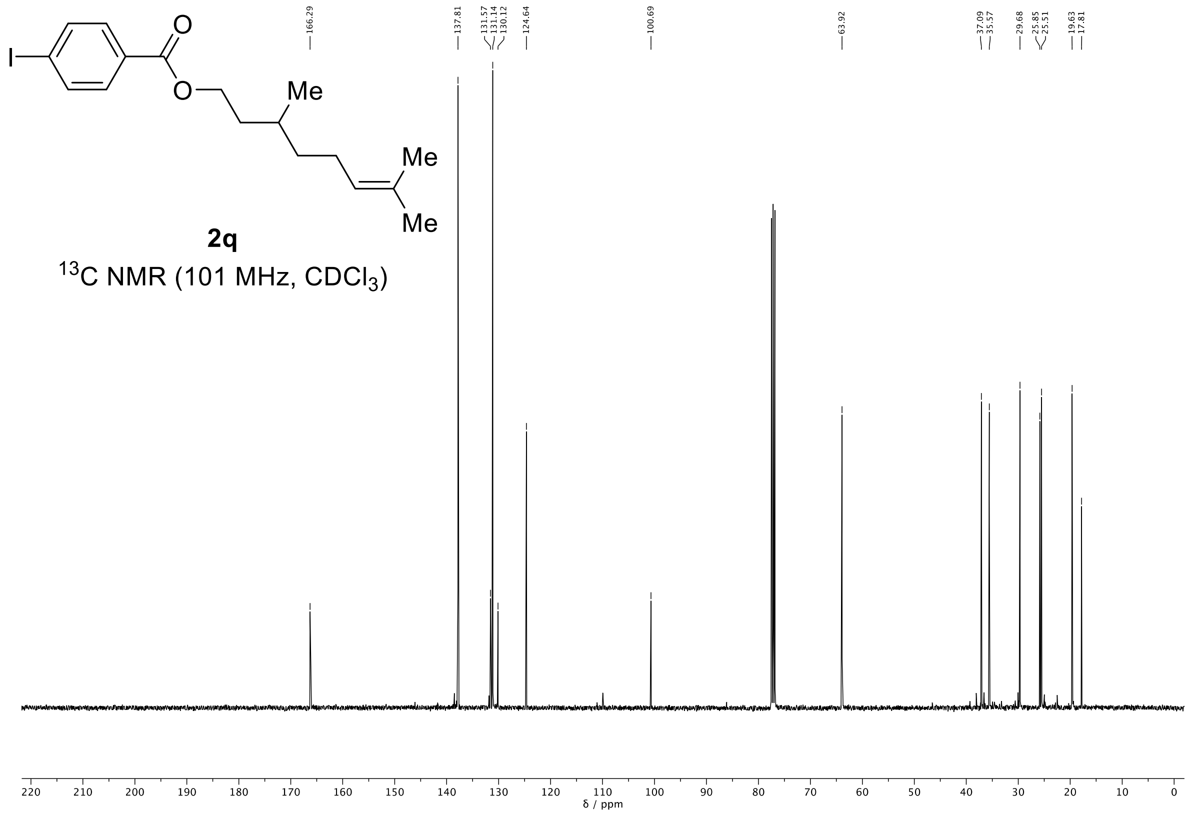
166.29
137.81
131.57
131.14
130.12
124.64
100.69
63.92
37.09
35.57
29.68
25.85
25.51
19.63
17.81
2q
^{13}C NMR (101 MHz, CDCl$_3$)
δ / ppm

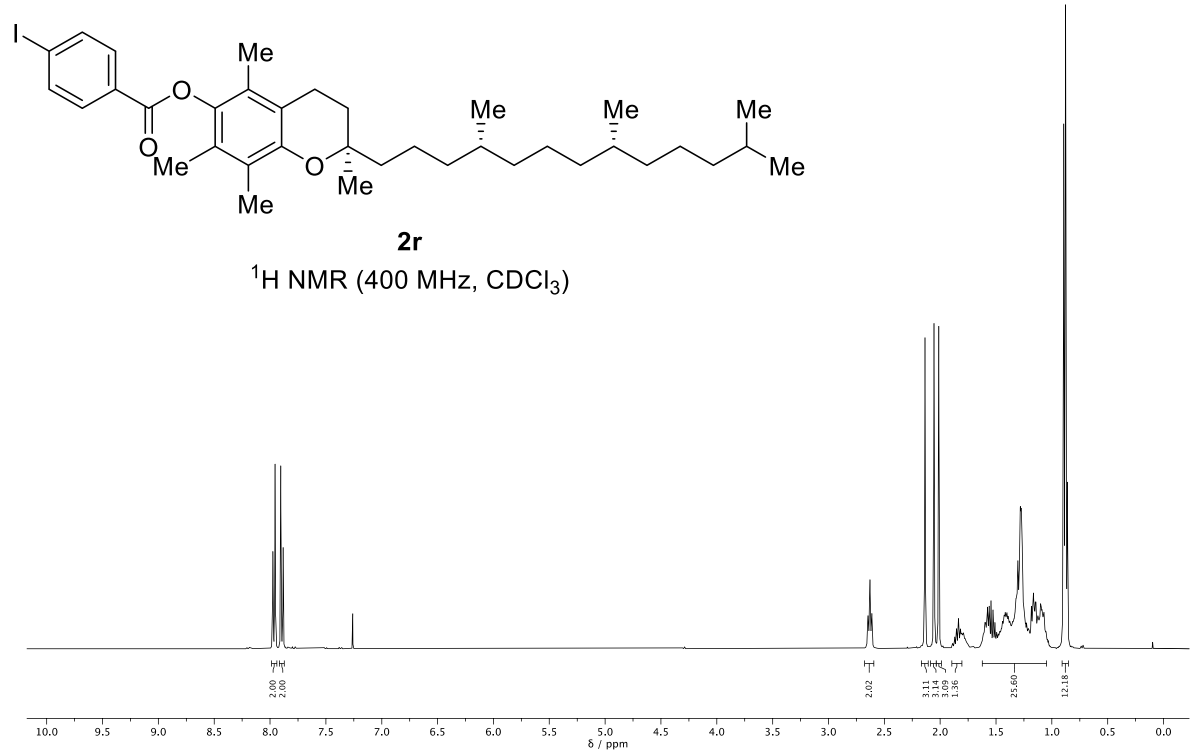
I
O
O
Me
Me
Me
Me
Me
Me
Me
Me
2r
¹H NMR (400 MHz, CDCl3)
2.00
2.00
2.02
3.11
3.14
3.09
1.36
25.60
12.18
δ / ppm

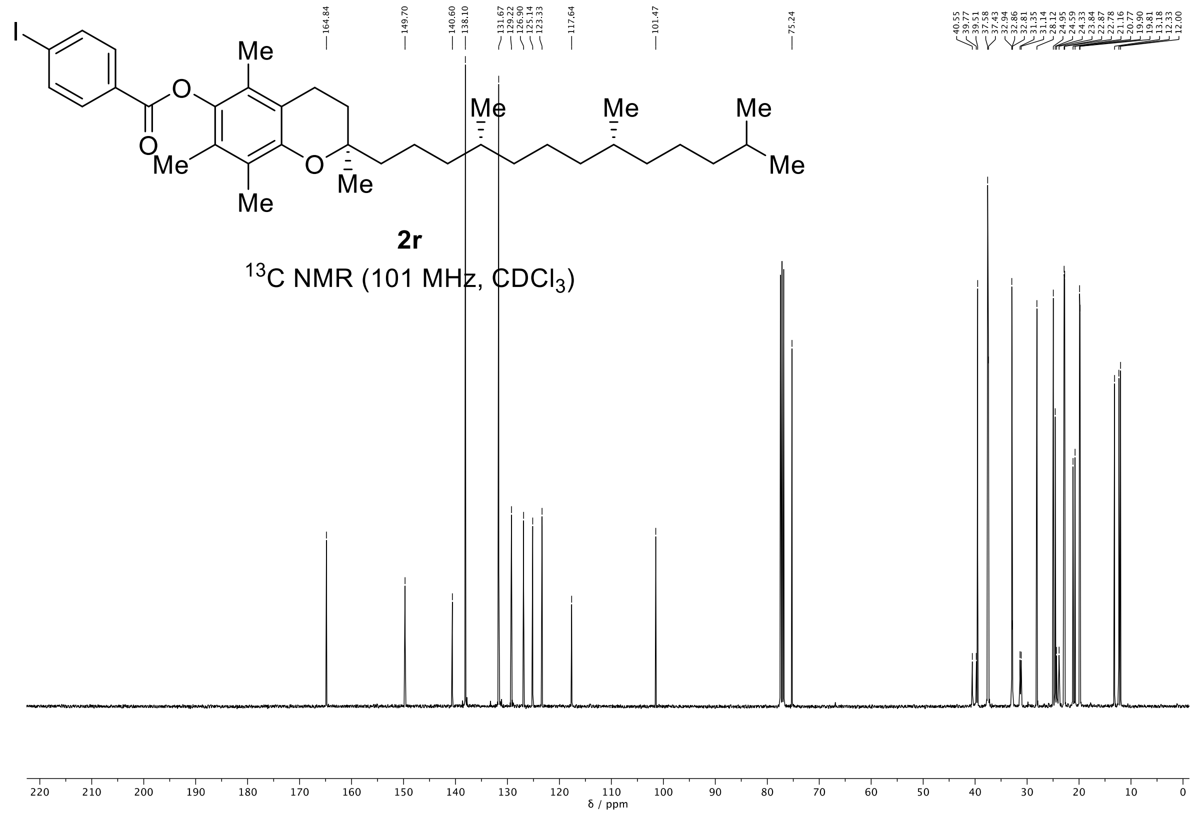
164.84
149.70
140.60
138.10
131.67
129.22
126.90
125.14
123.33
117.64
101.47
75.24
40.55
39.77
39.51
37.58
37.43
32.94
32.86
32.81
31.35
31.14
28.12
24.95
24.59
24.33
23.84
22.87
22.78
21.16
20.77
19.90
19.81
13.18
12.33
12.00
2r
¹³C NMR (101 MHz, CDCl3)
δ / ppm

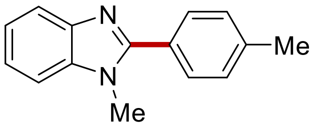

3a

^{1}H NMR (400 MHz, $CDCl_3$)

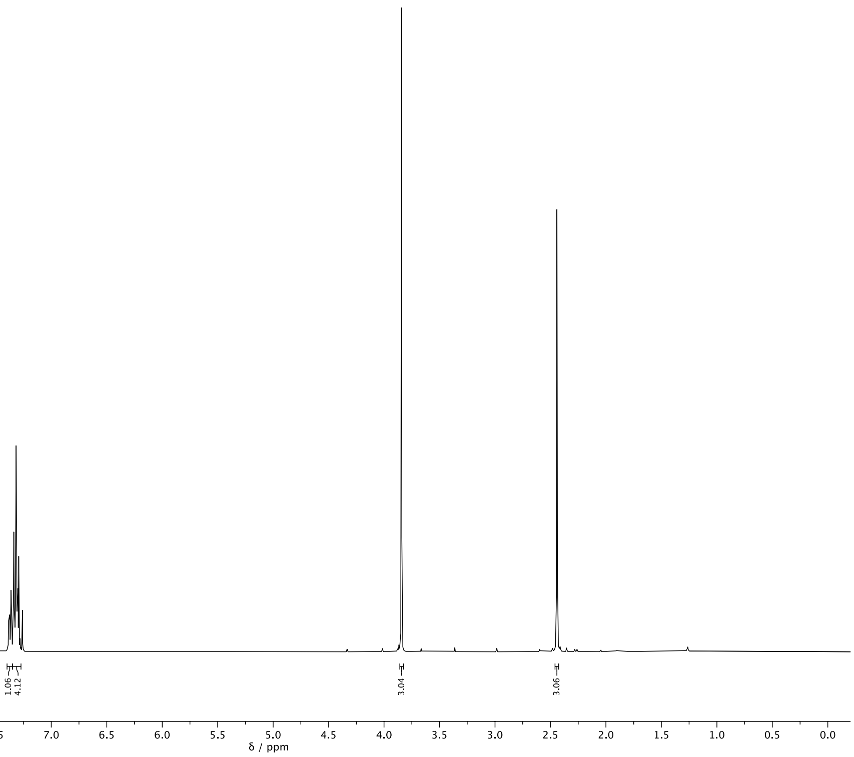

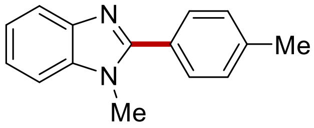

3a

^{13}C NMR (101 MHz, $CDCl_3$)

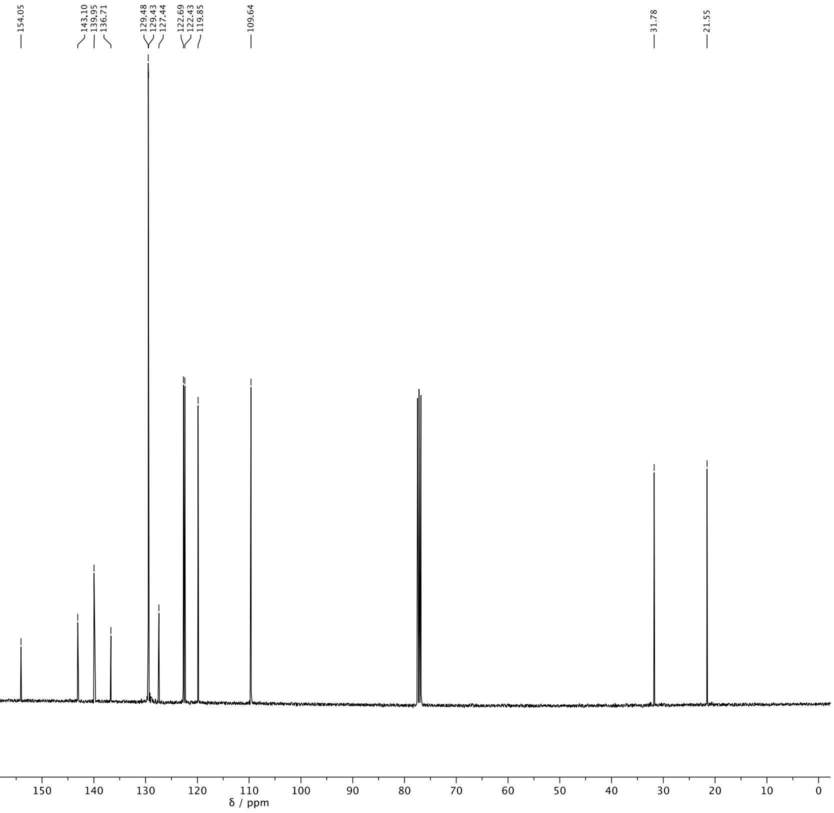

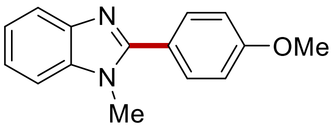

3b

^{1}H NMR (400 MHz, $CDCl_3$)

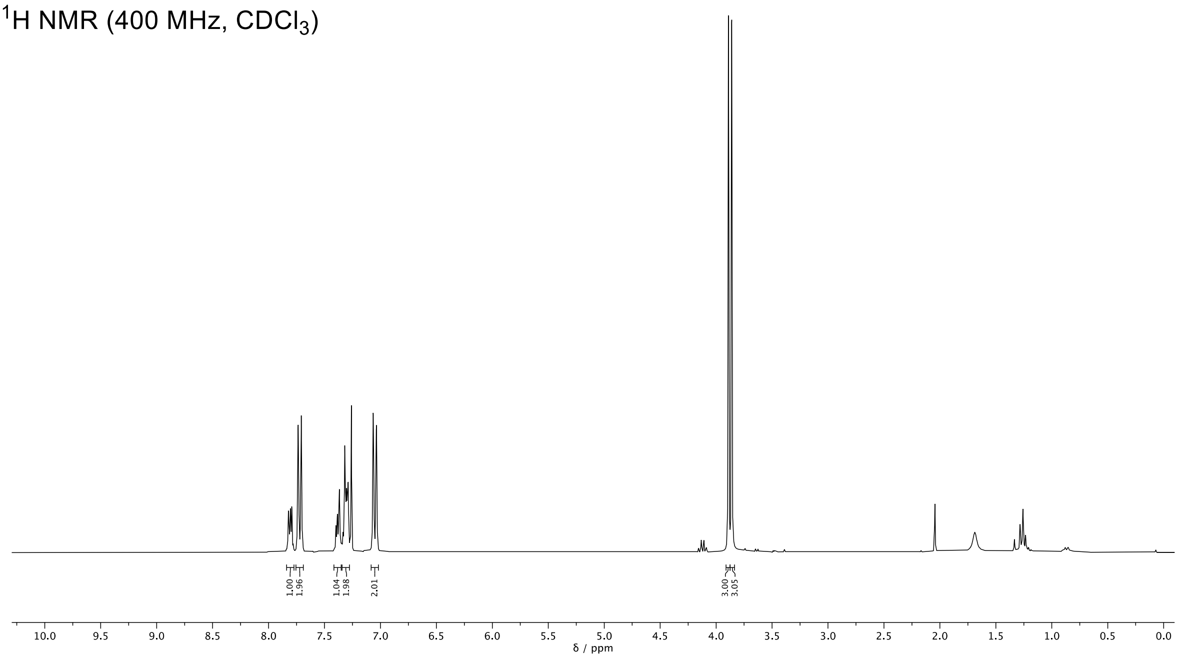

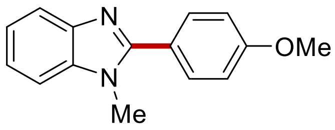

3b

^{13}C NMR (101 MHz, $CDCl_3$)

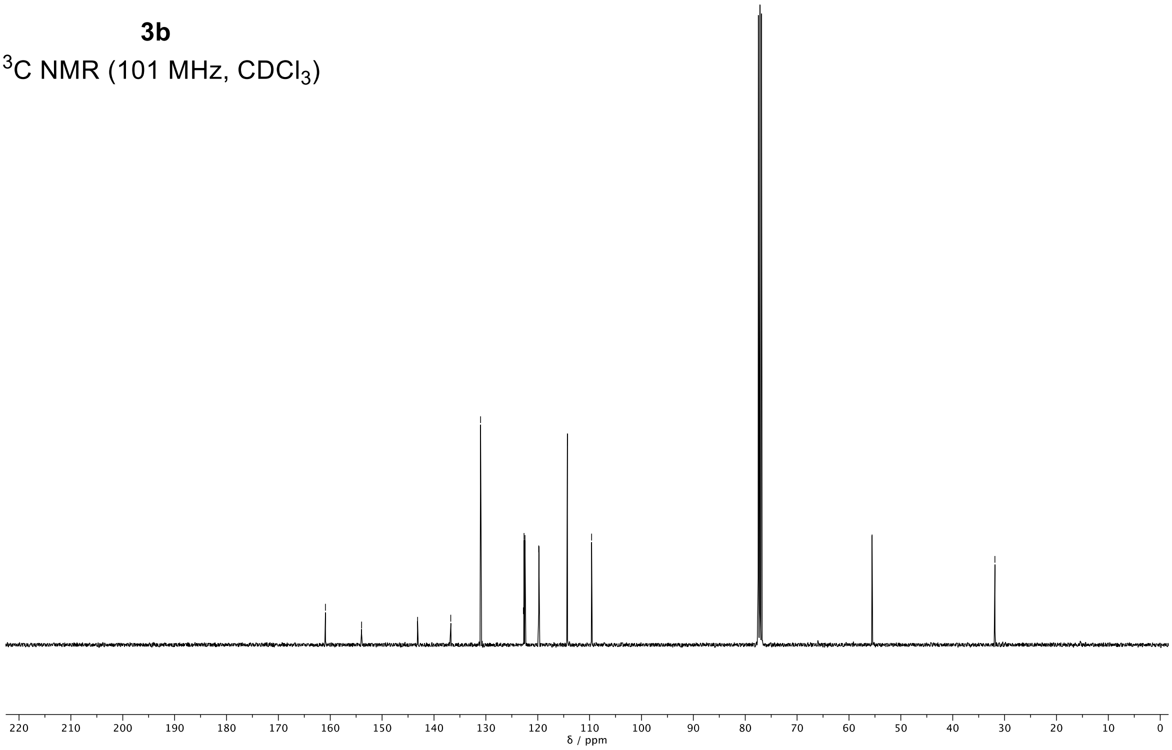

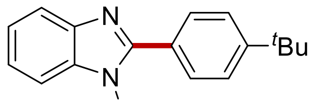

3c

^{1}H NMR (400 MHz, CDCl$_3$)

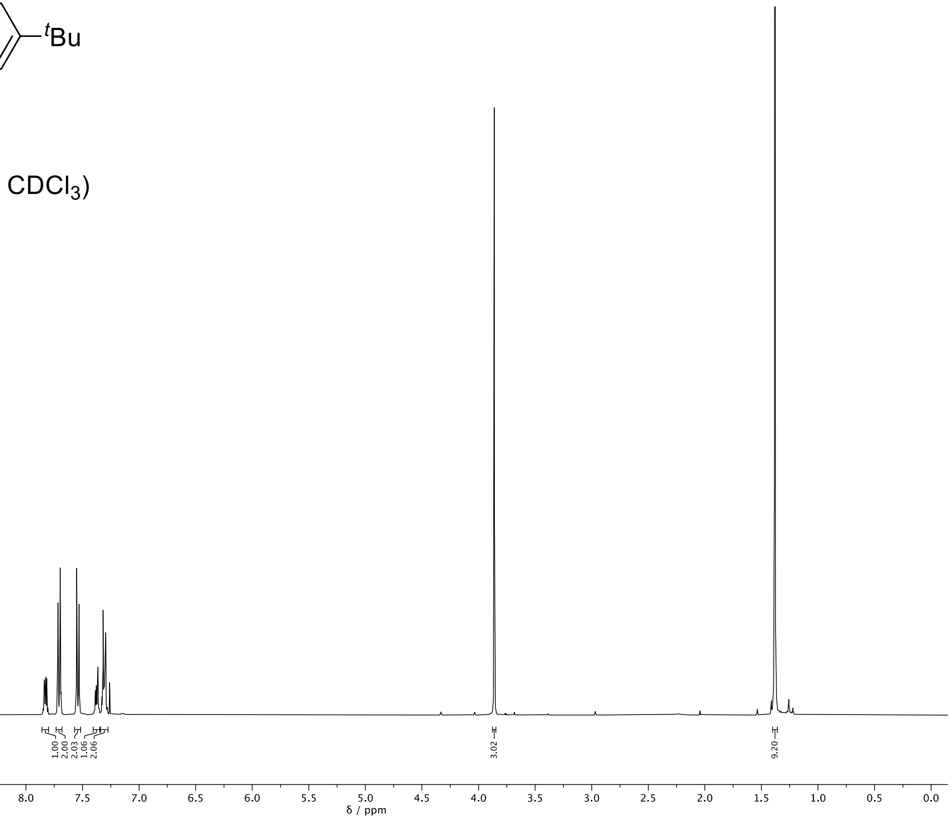

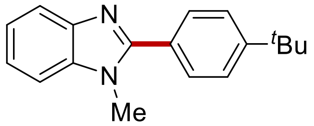

3c

^{13}C NMR (101 MHz, CDCl$_3$)

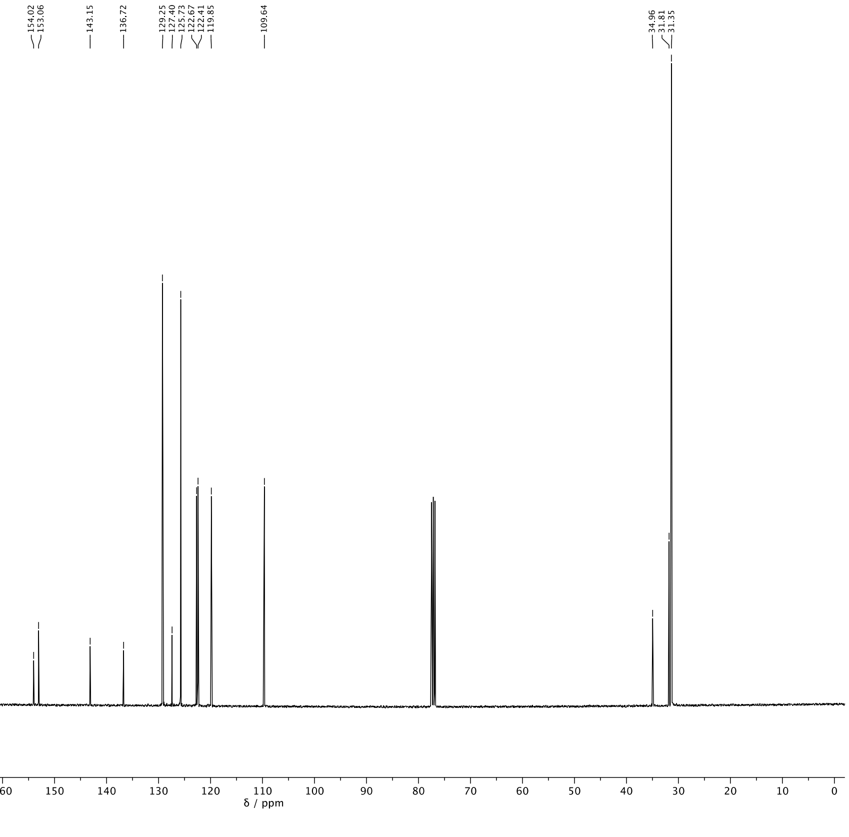

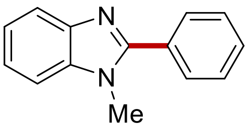

3d

^{1}H NMR (400 MHz, $CDCl_3$)

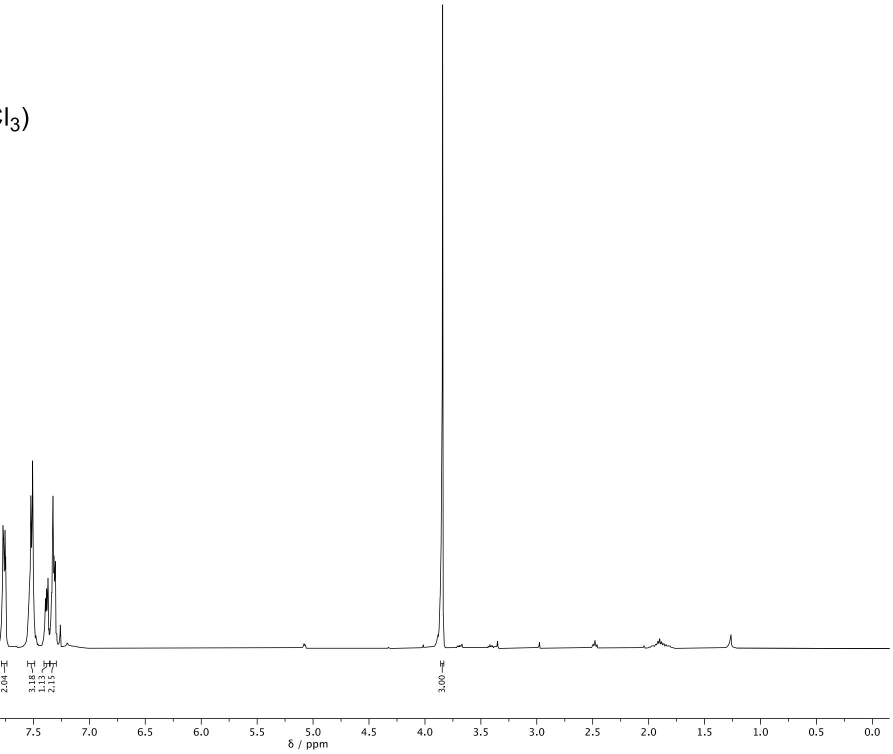

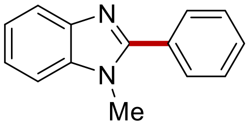

3d

^{13}C NMR (101 MHz, $CDCl_3$)

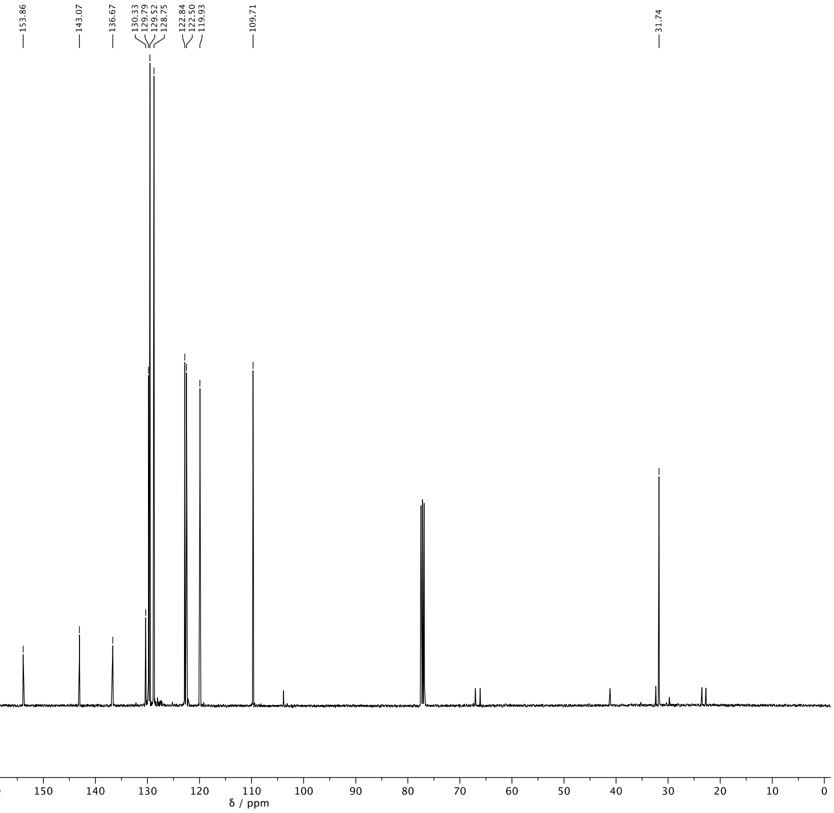

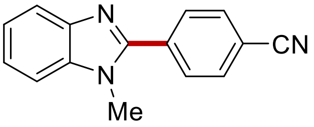

3e

^{1}H NMR (400 MHz, CDCl$_3$)

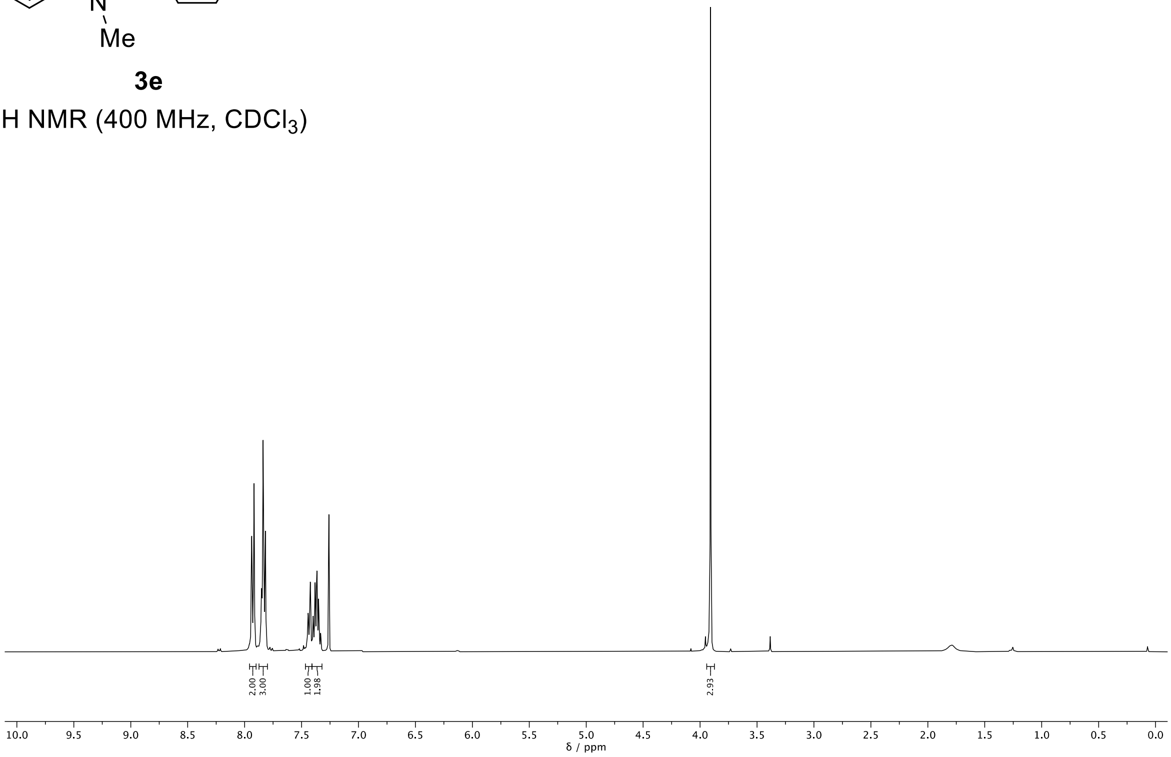

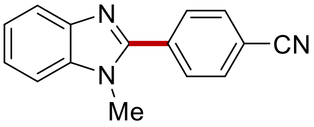

3e

^{13}C NMR (101 MHz, CDCl$_3$)

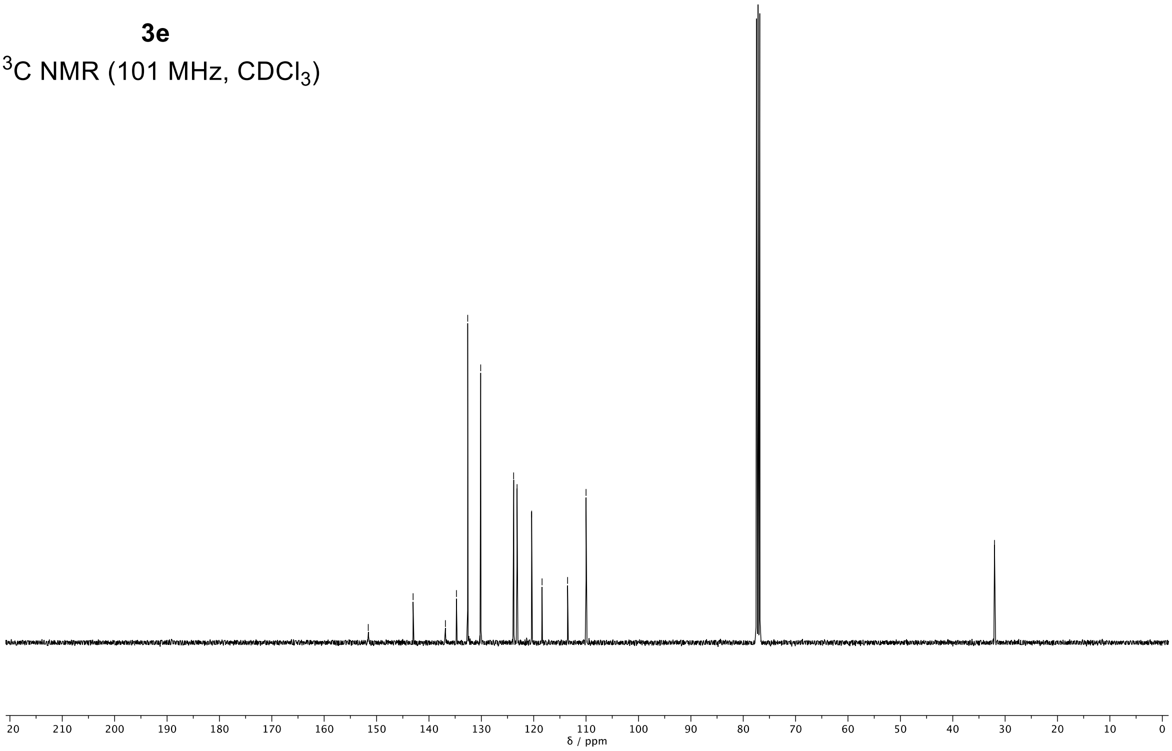

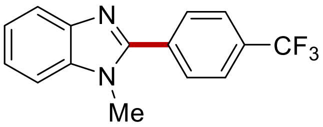

3f

^1H NMR (400 MHz, $CDCl_3$)

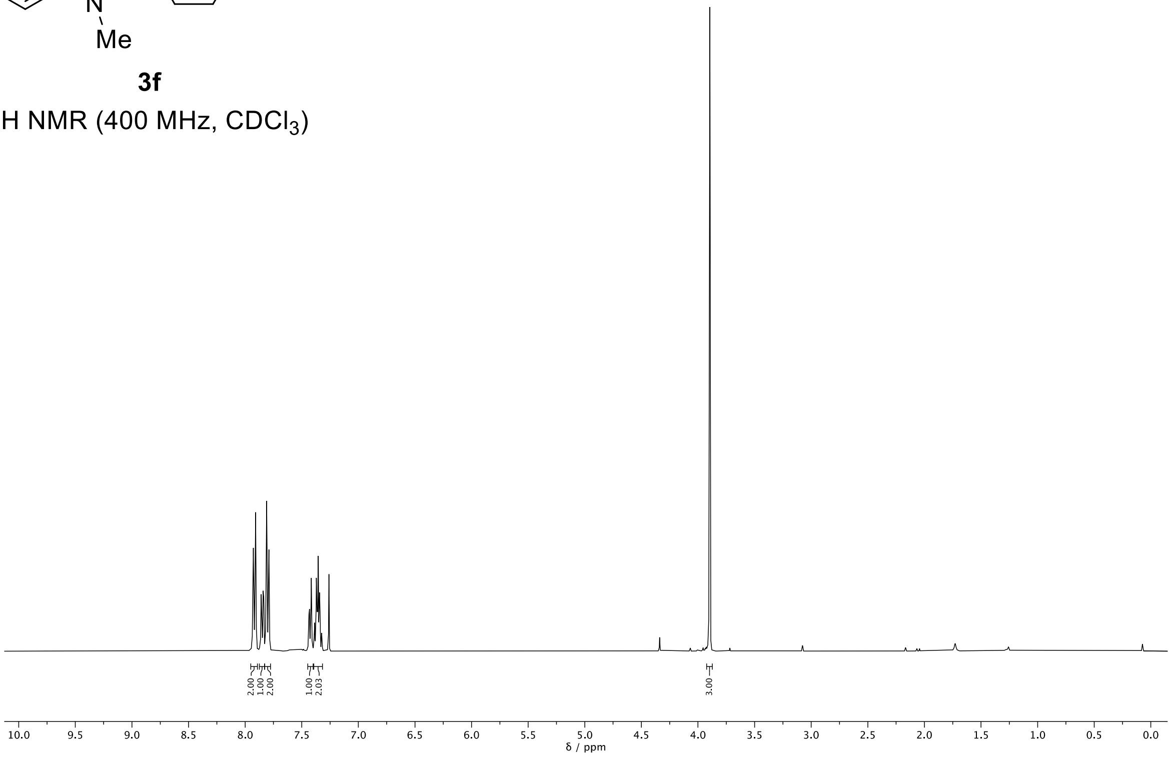

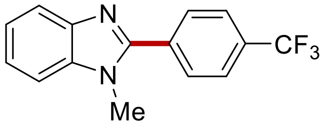

3f

^{13}C NMR (101 MHz, $CDCl_3$)

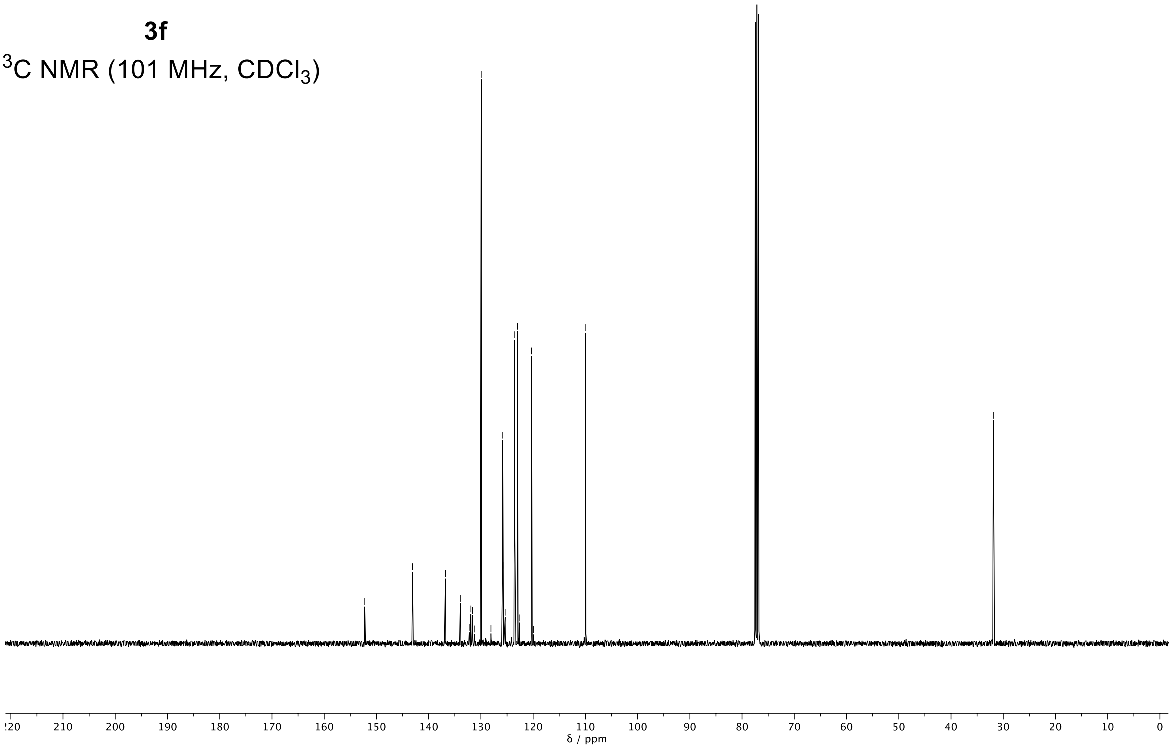

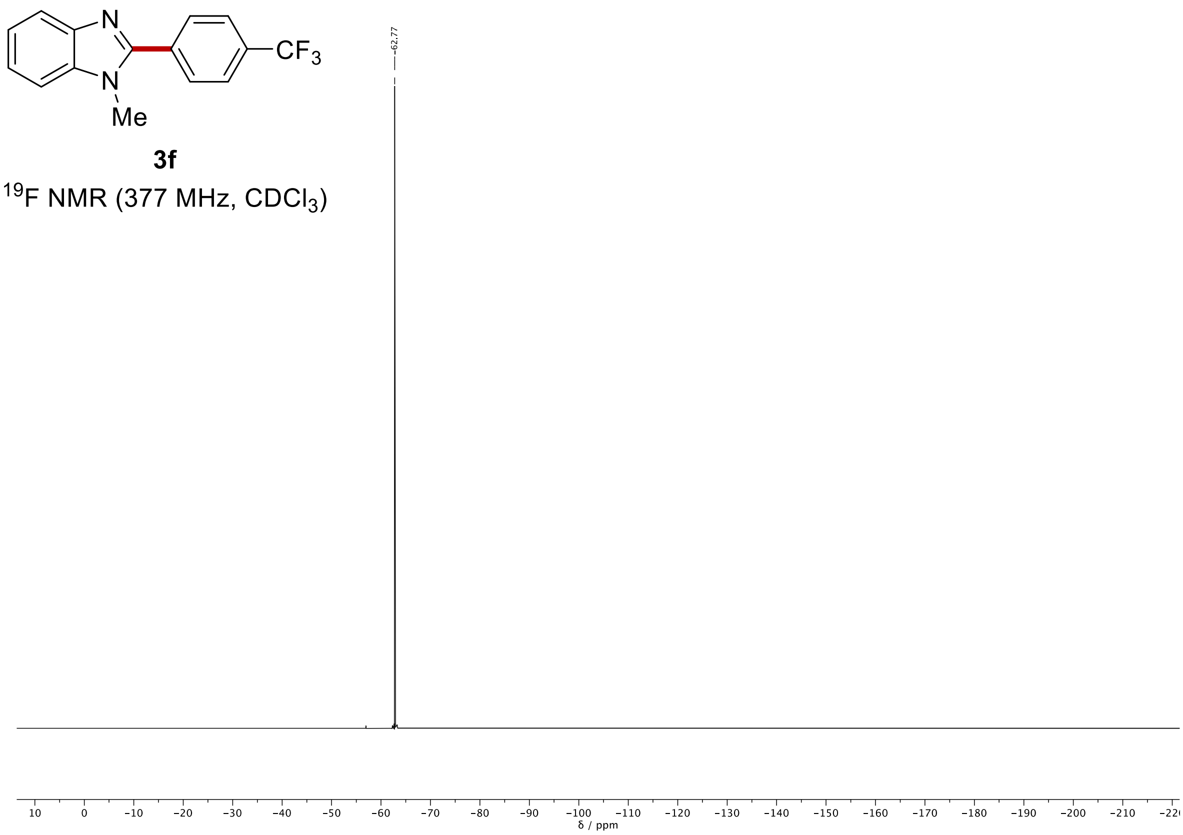
N
N
Me
CF3
3f
19F NMR (377 MHz, CDCl3)
-62.77
δ / ppm

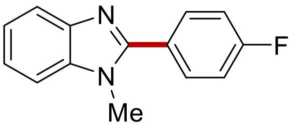

3g

^{1}H NMR (400 MHz, $CDCl_3$)

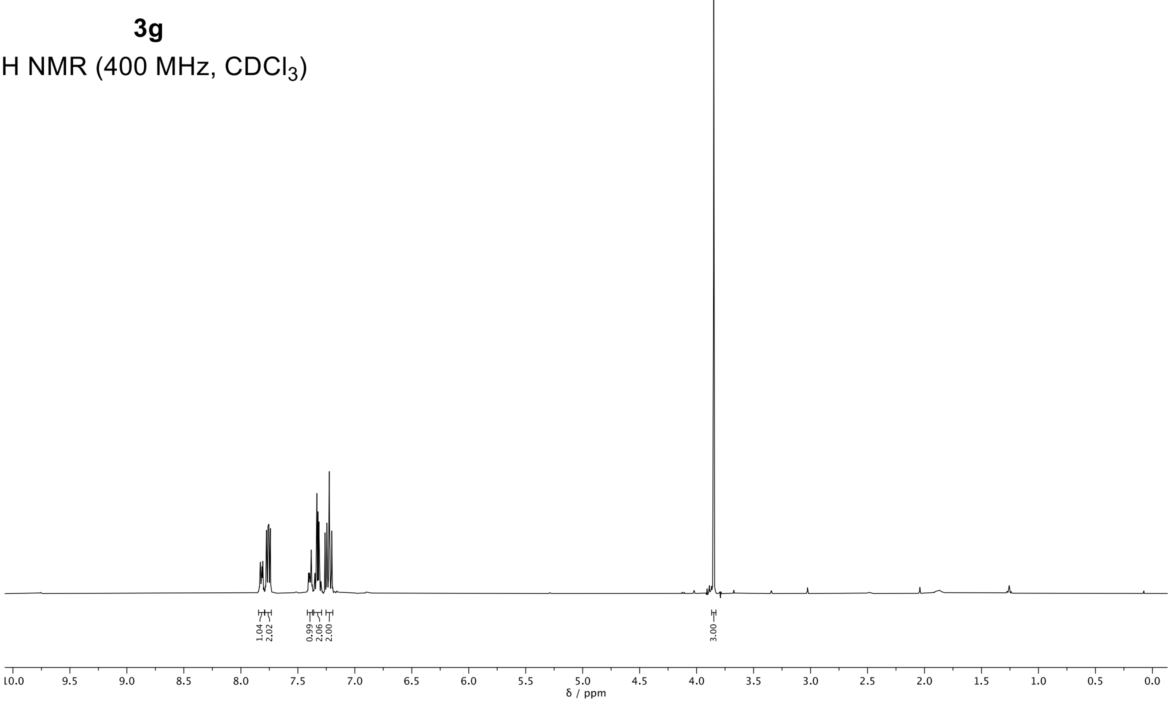

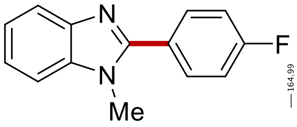

3g

^{13}C NMR (101 MHz, $CDCl_3$)

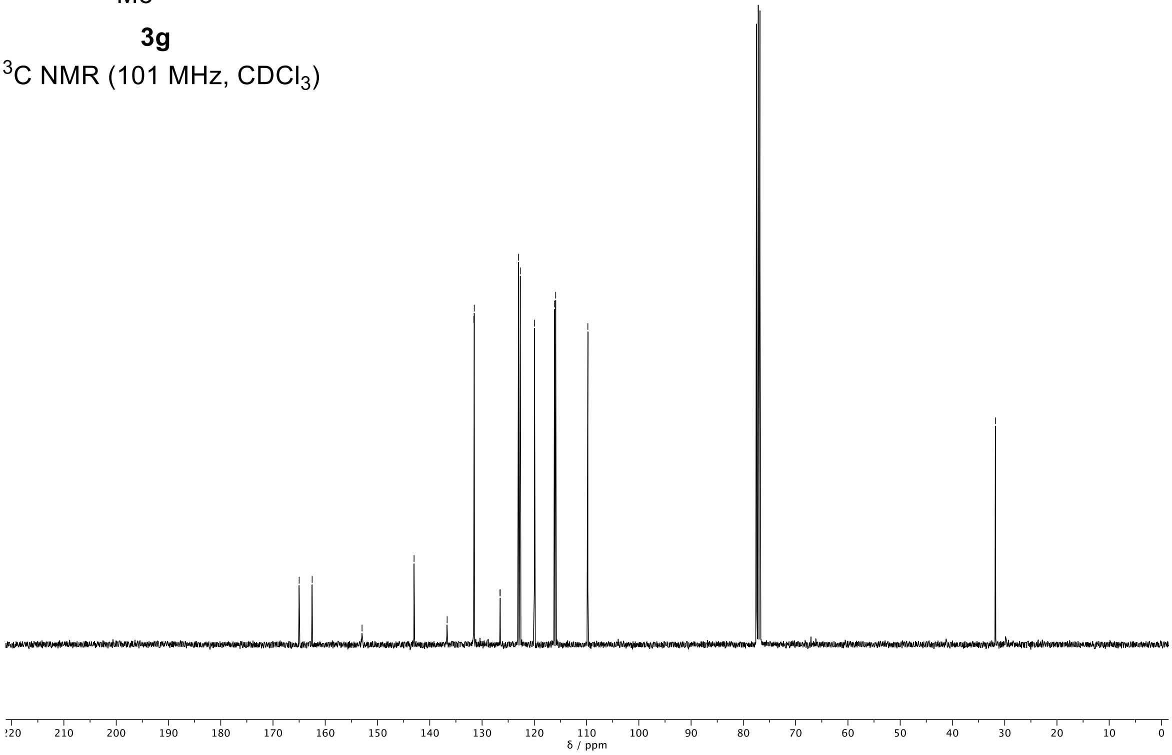

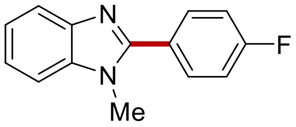

3g

^{19}F NMR (377 MHz, $CDCl_3$)

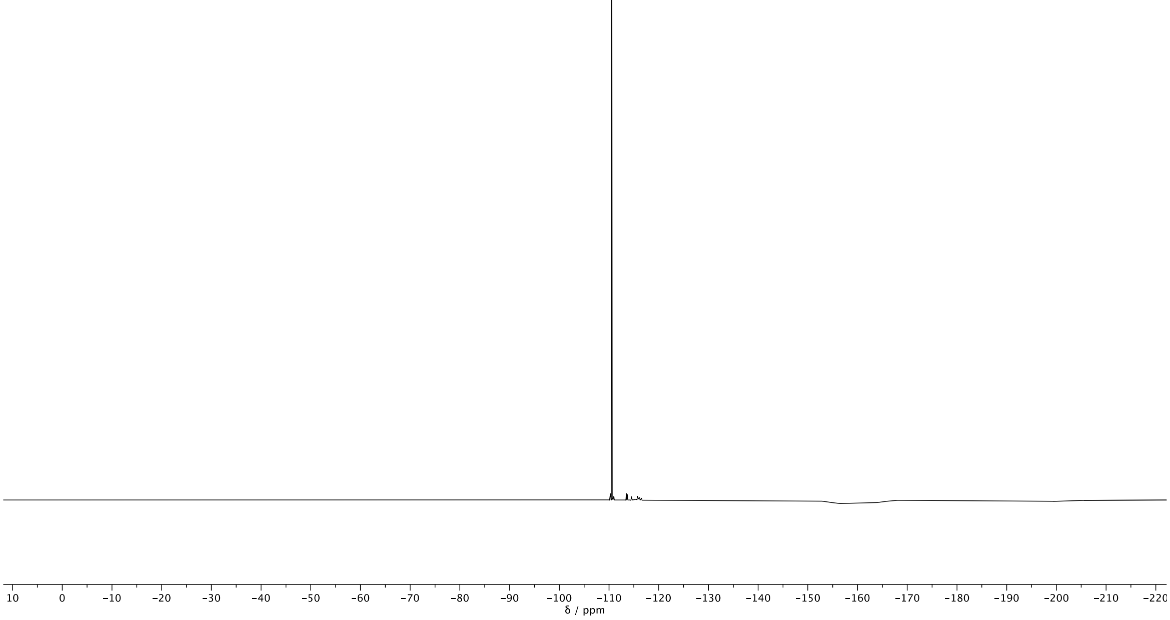

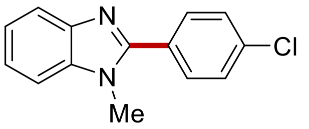

3h

^{1}H NMR (400 MHz, $CDCl_3$)

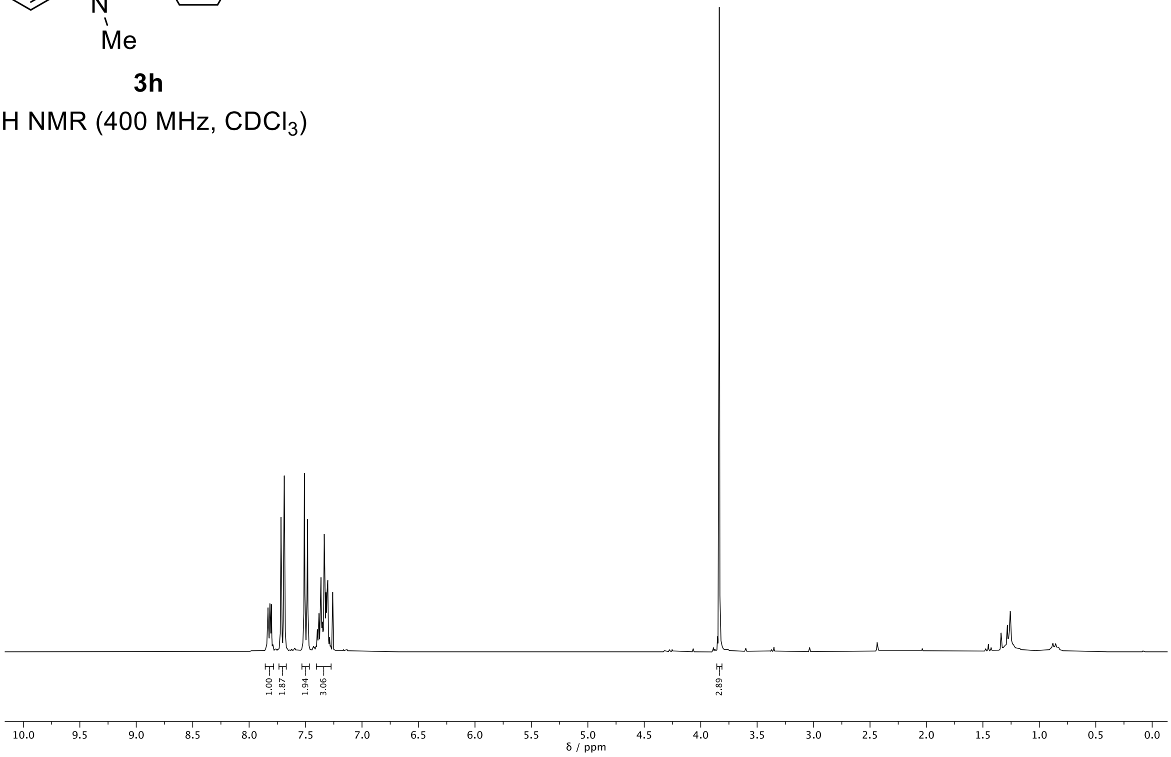

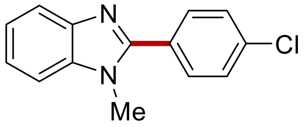

3h

^{13}C NMR (101 MHz, $CDCl_3$)

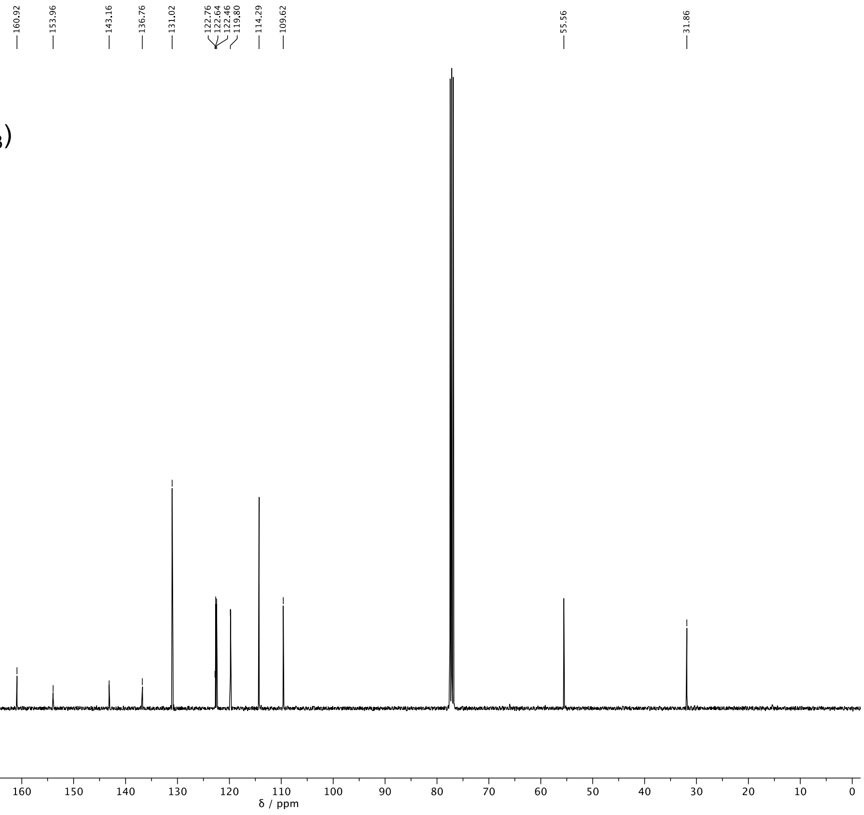

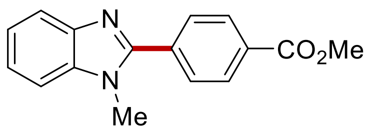

3i

^1H NMR (400 MHz, $CDCl_3$)

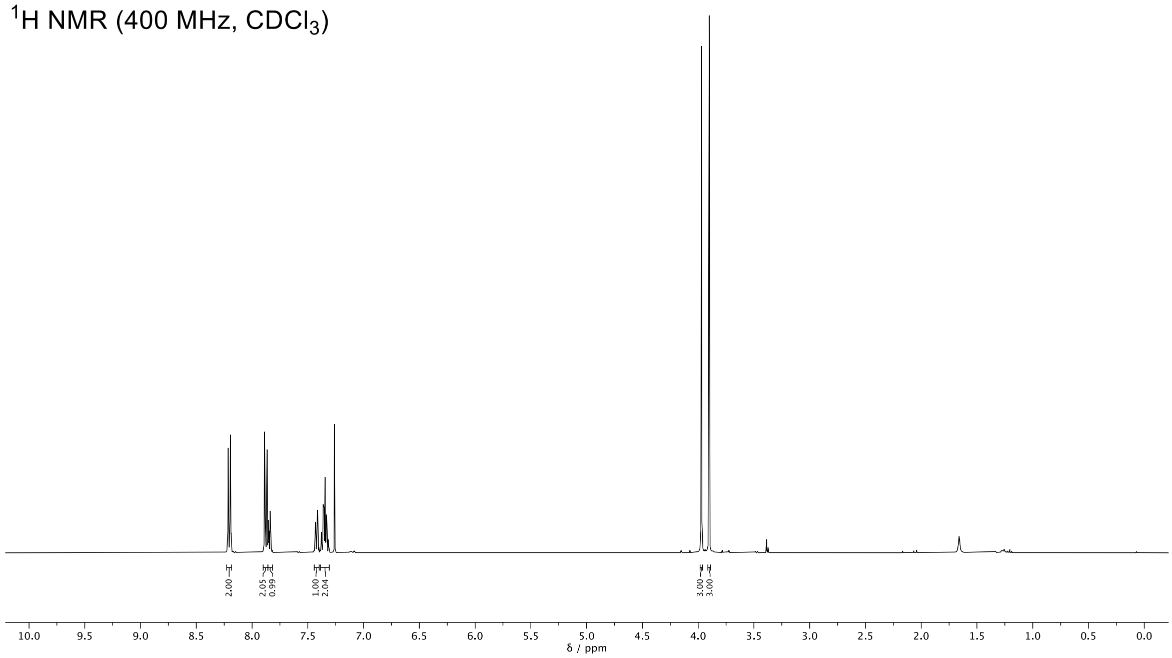

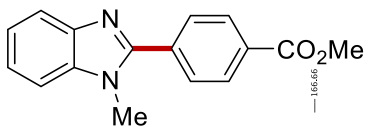

3i

^{13}C NMR (101 MHz, $CDCl_3$)

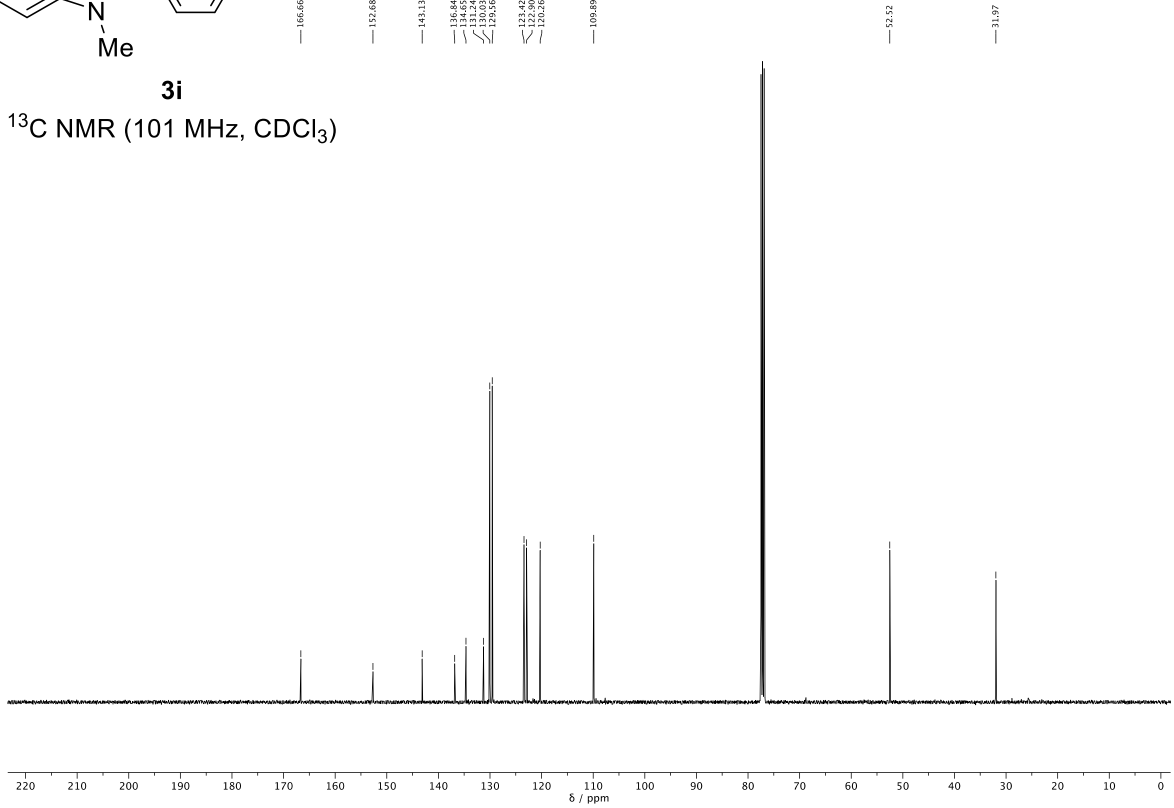

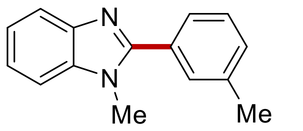

3j

^{1}H NMR (400 MHz, $CDCl_3$)

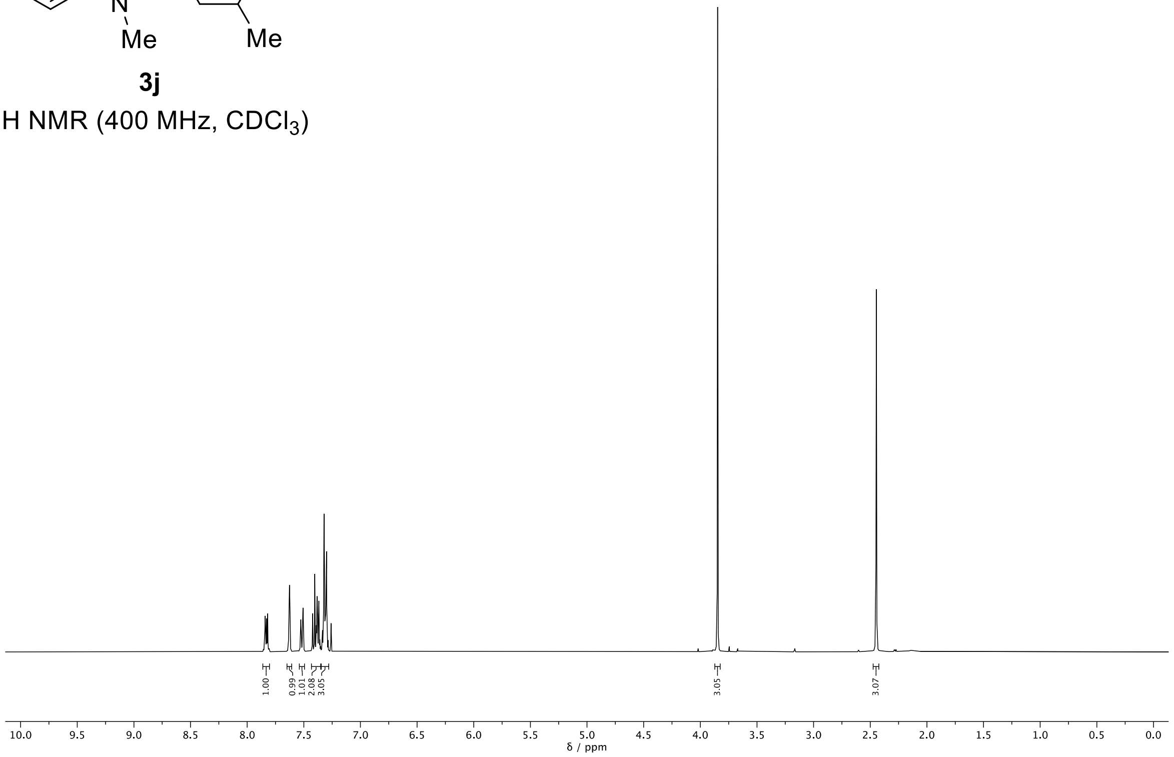

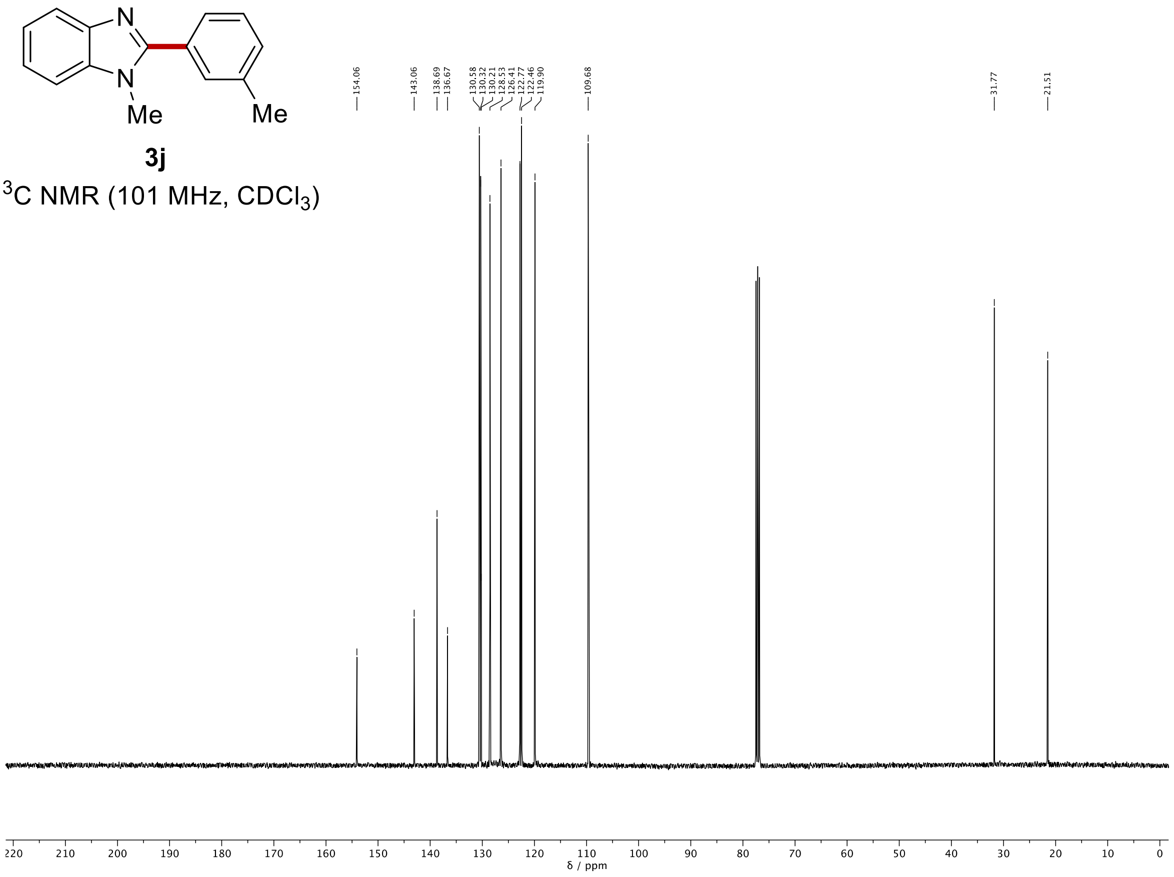

3j

^{13}C NMR (101 MHz, $CDCl_3$)

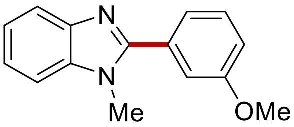

3k

^{1}H NMR (400 MHz, $CDCl_3$)

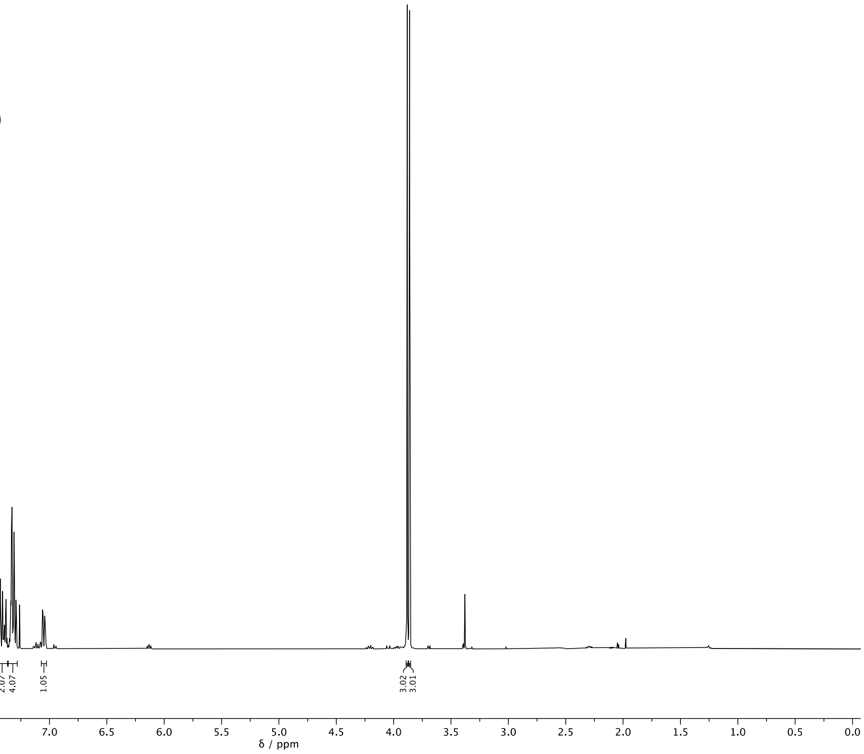

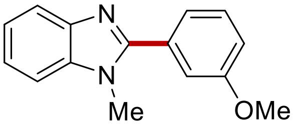

3k

^{13}C NMR (101 MHz, $CDCl_3$)

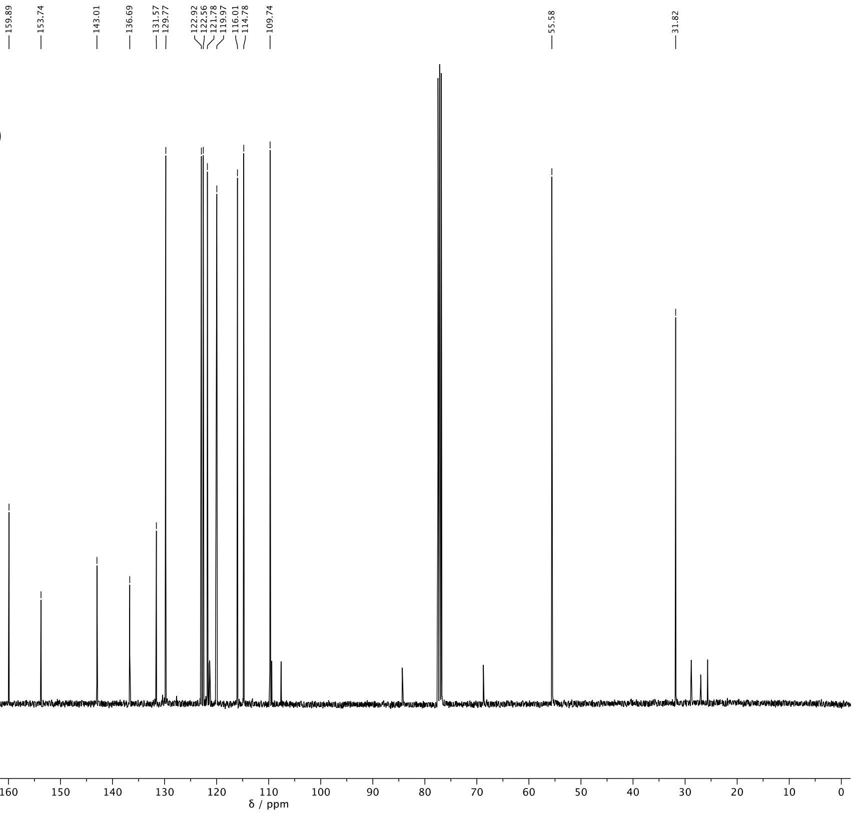

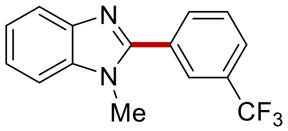

3l

^{1}H NMR (400 MHz, $CDCl_3$)

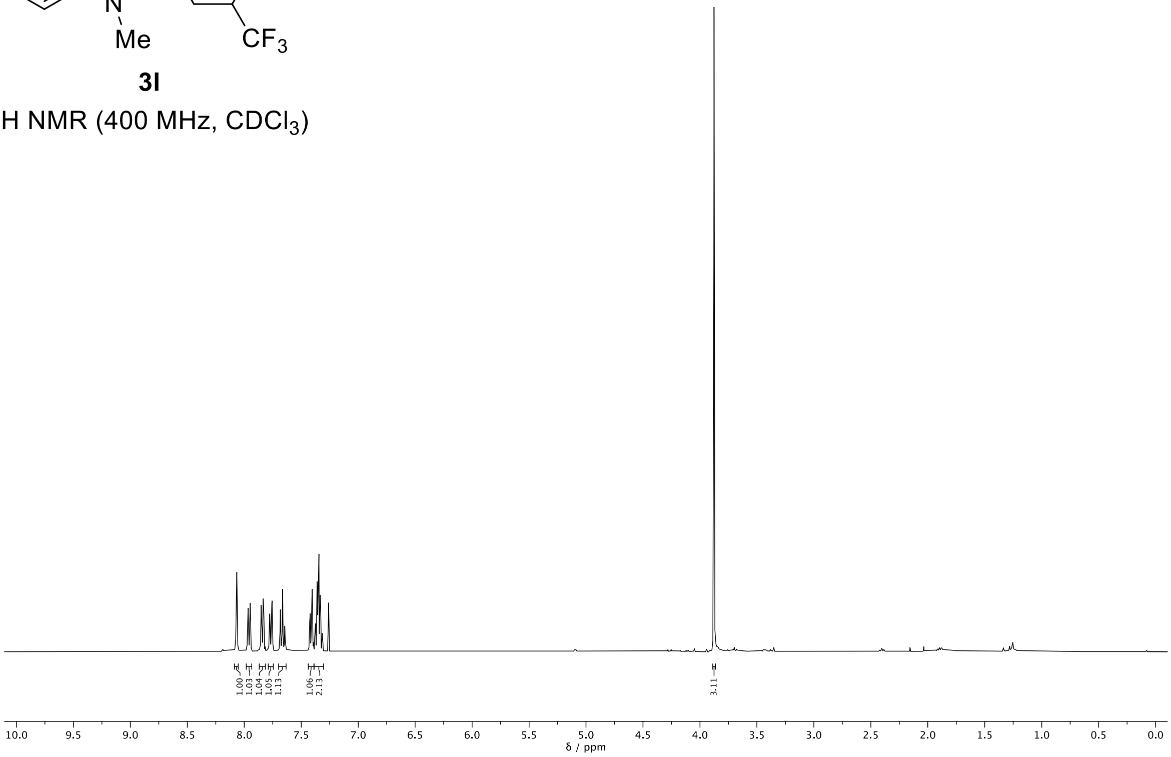

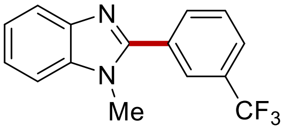

3l

^{13}C NMR (101 MHz, $CDCl_3$)

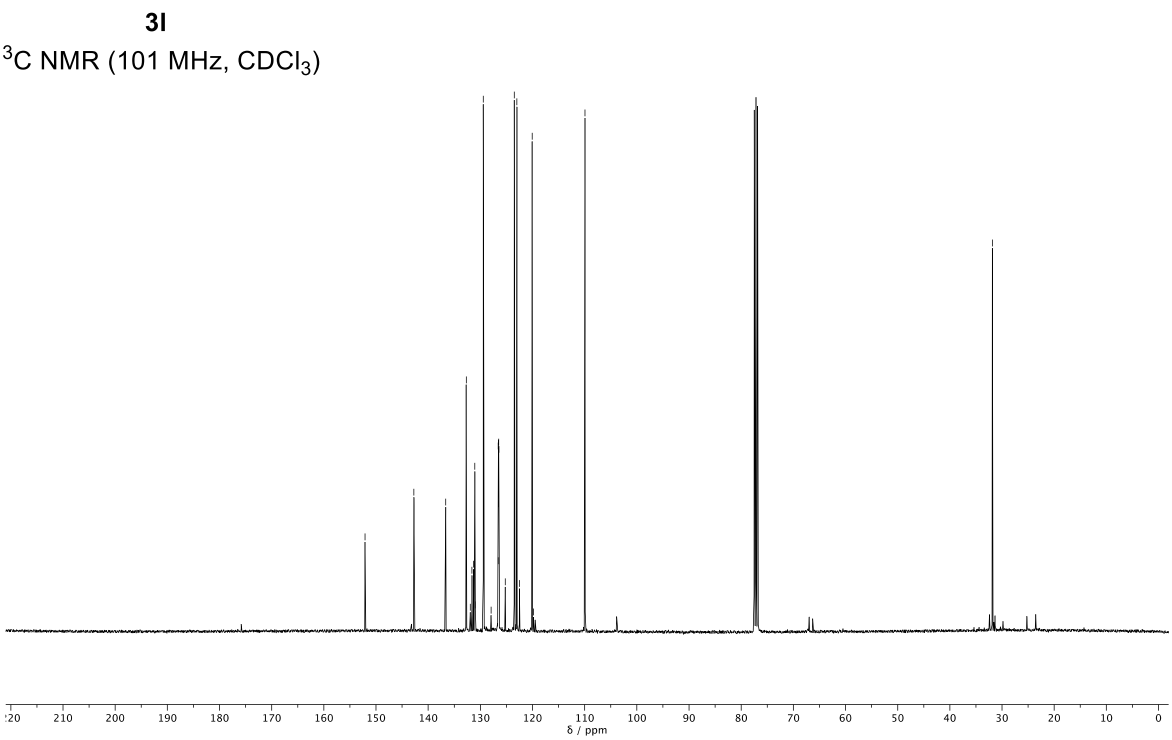

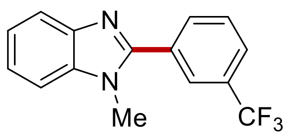

3l

^{19}F NMR (377 MHz, $CDCl_3$)

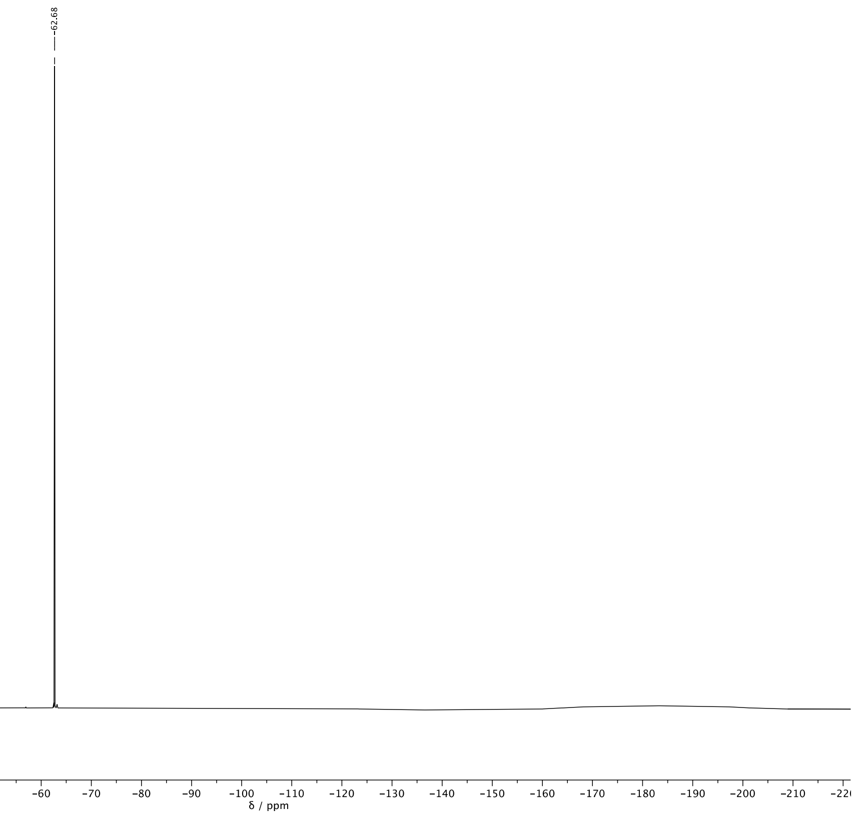

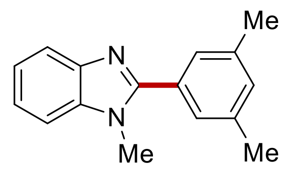

3m

^{1}H NMR (400 MHz, $CDCl_3$)

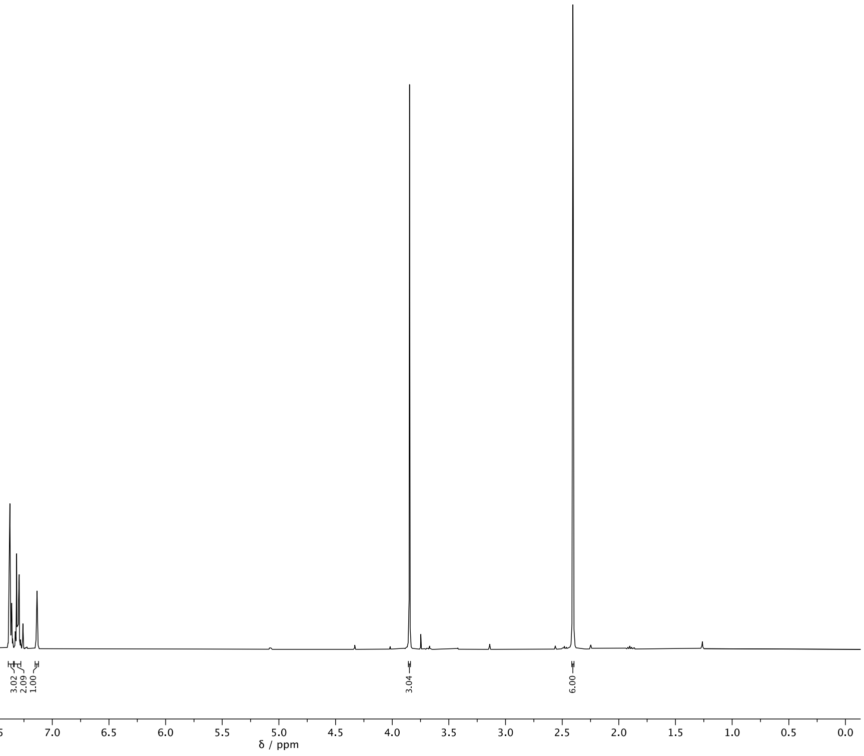

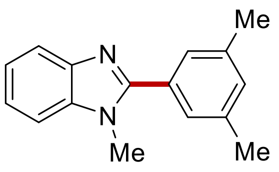

3m

^{13}C NMR (101 MHz, $CDCl_3$)

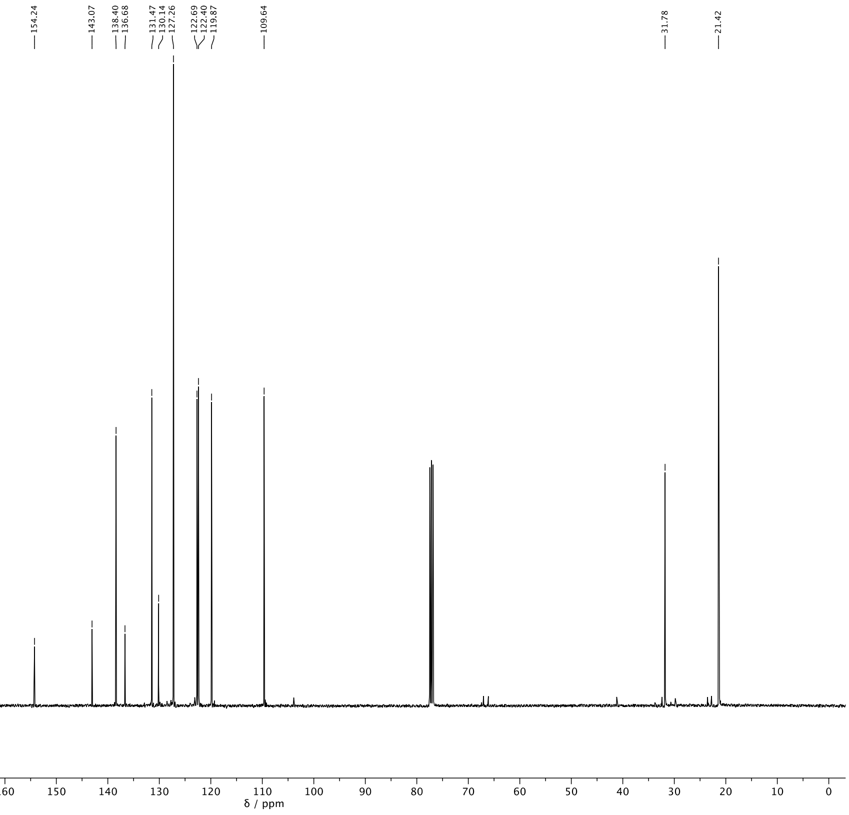

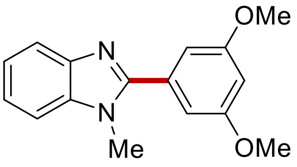

3n

^{1}H NMR (400 MHz, $CDCl_3$)

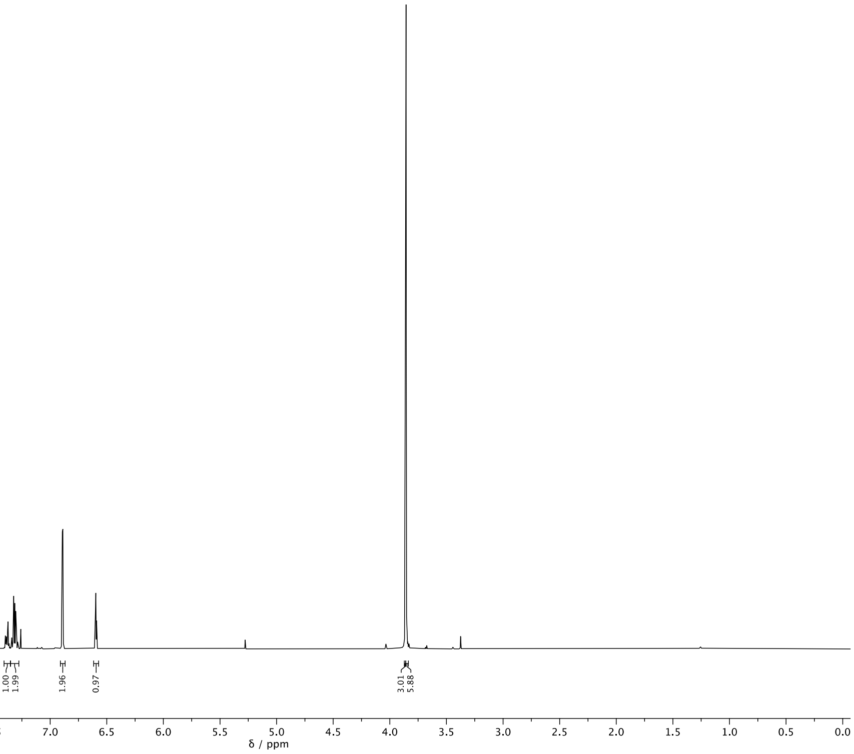

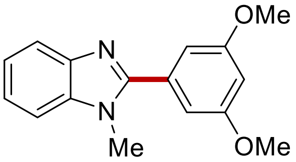

3n

^{13}C NMR (101 MHz, $CDCl_3$)

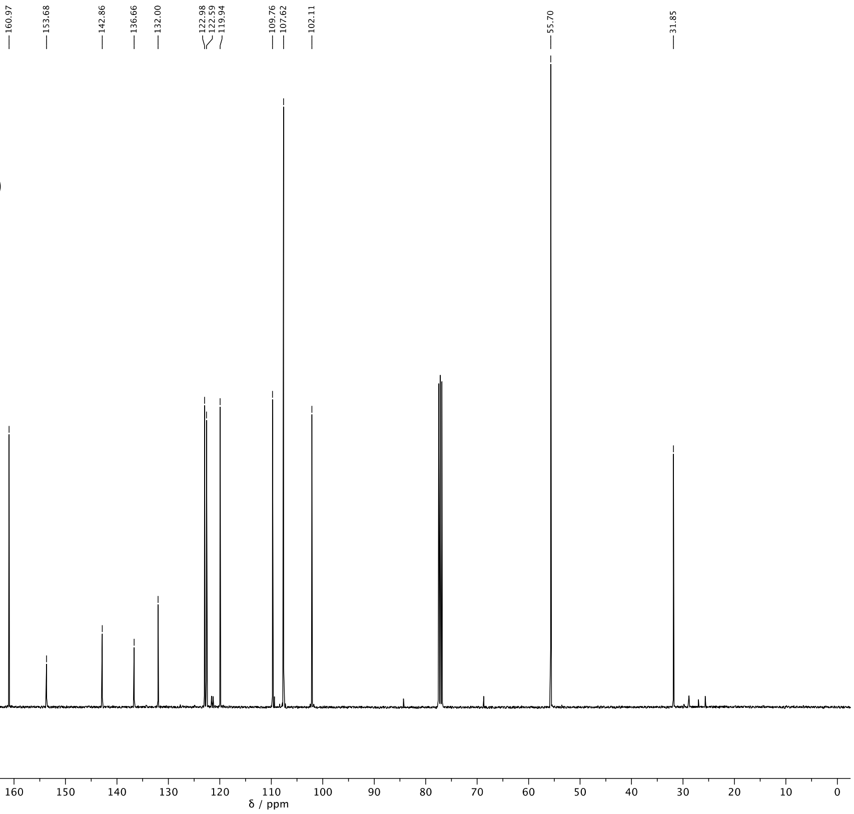

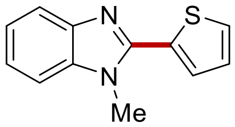

3o

^{1}H NMR (400 MHz, $CDCl_3$)

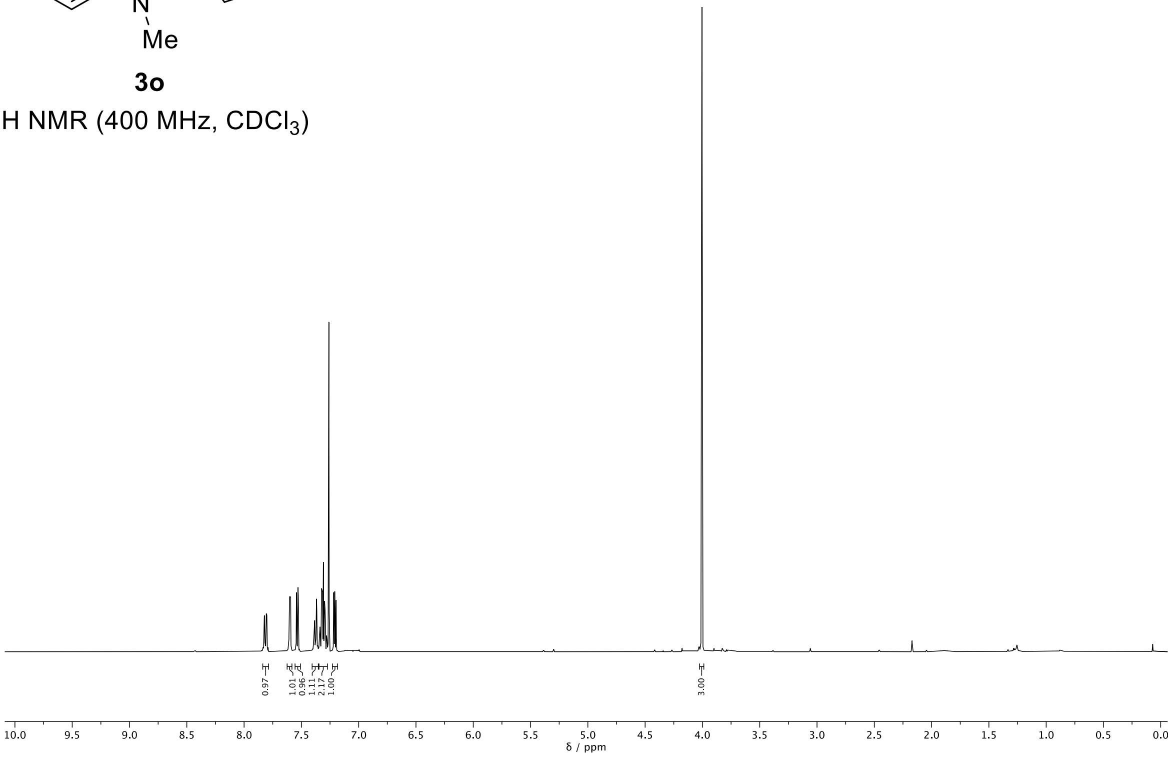

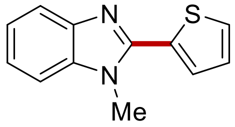

3o

^{13}C NMR (101 MHz, $CDCl_3$)

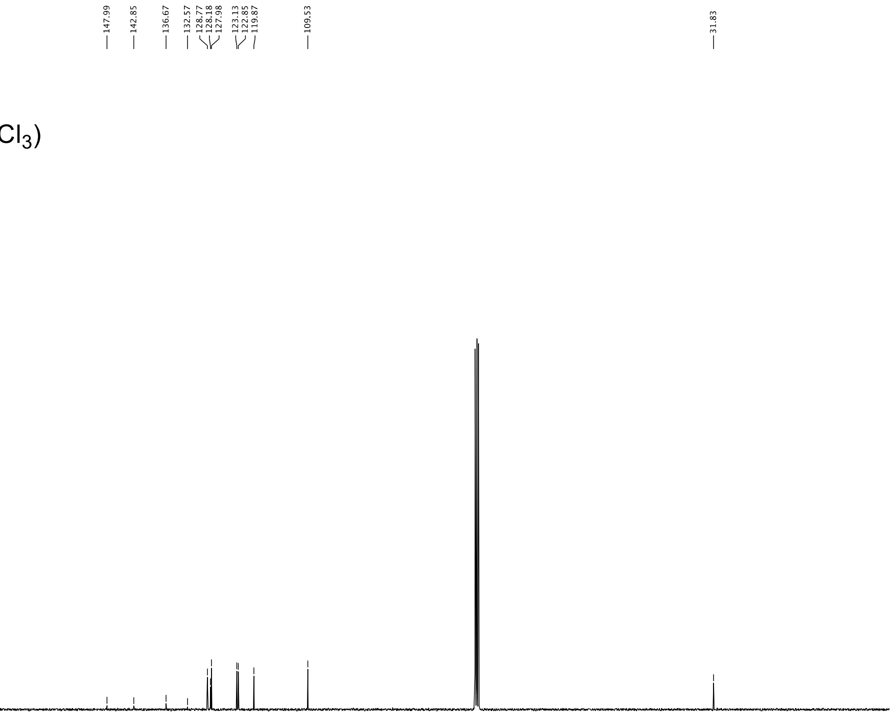

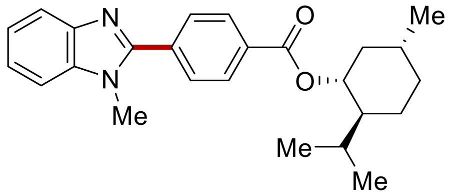

3p

^{1}H NMR (400 MHz, $CDCl_3$)

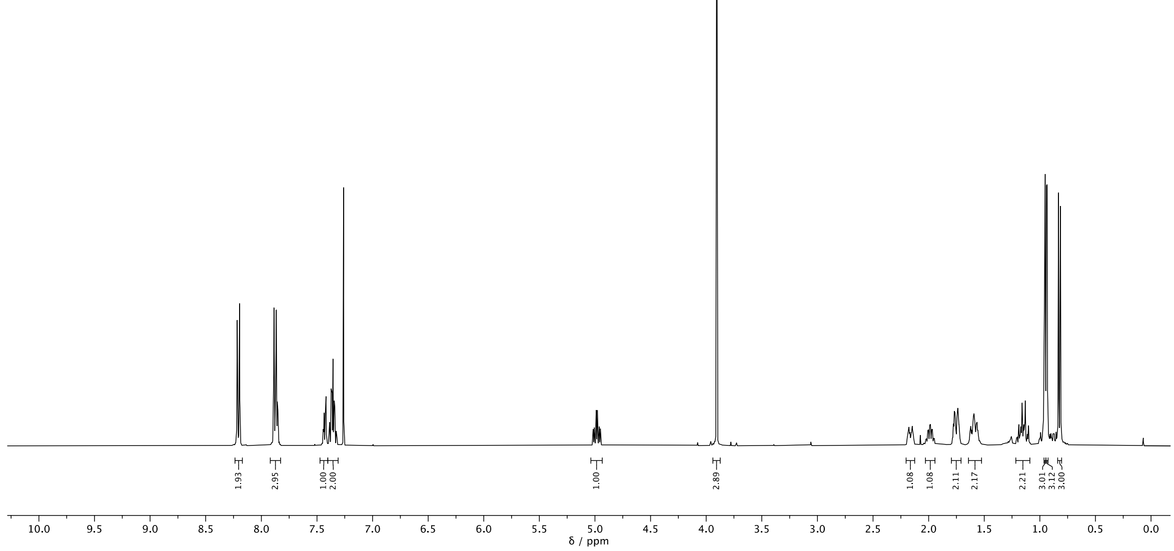

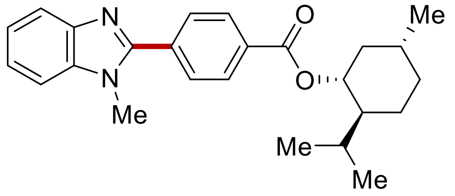

3p

^{13}C NMR (101 MHz, $CDCl_3$)

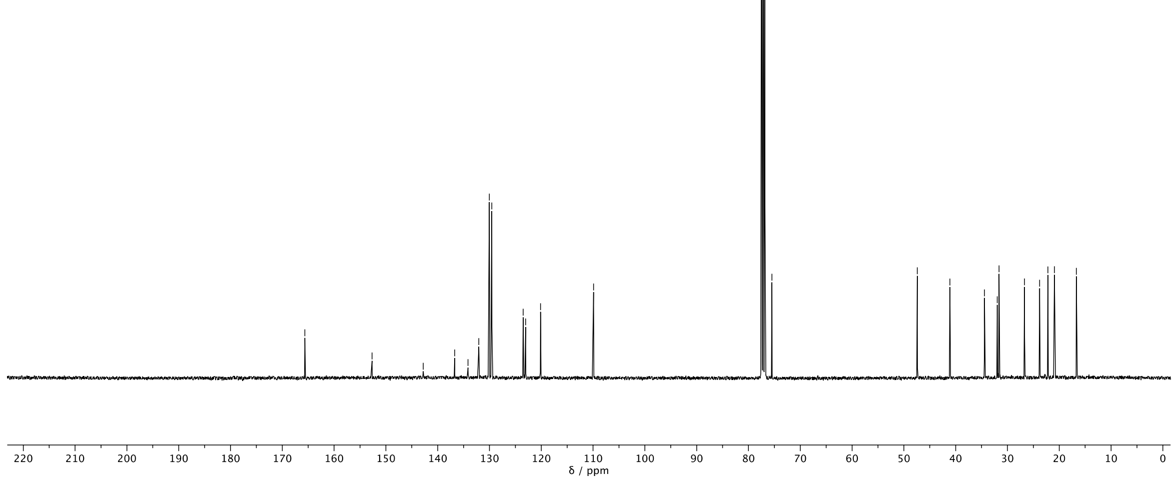

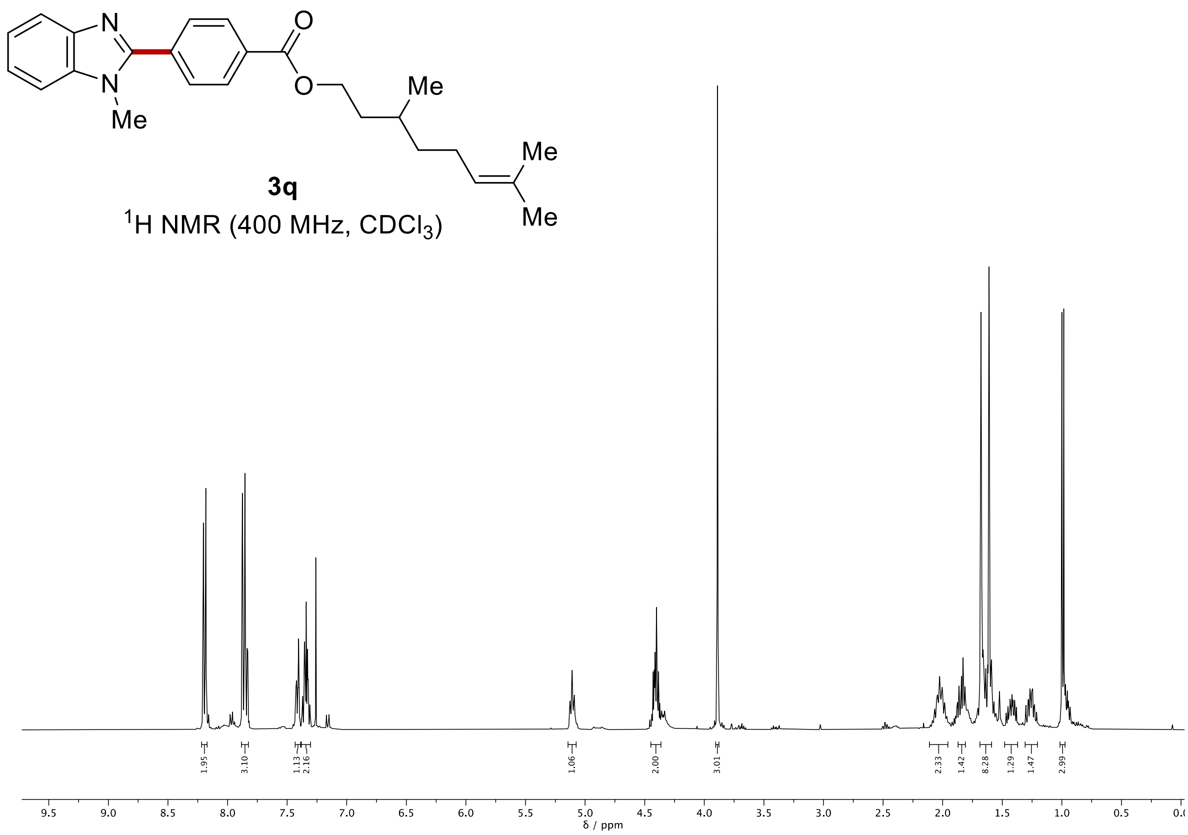
3q
1H NMR (400 MHz, CDCl3)
1.95 3.10 1.13 2.16 1.06 2.00 3.01 2.33 1.42 8.28 1.29 1.47 2.99
δ / ppm

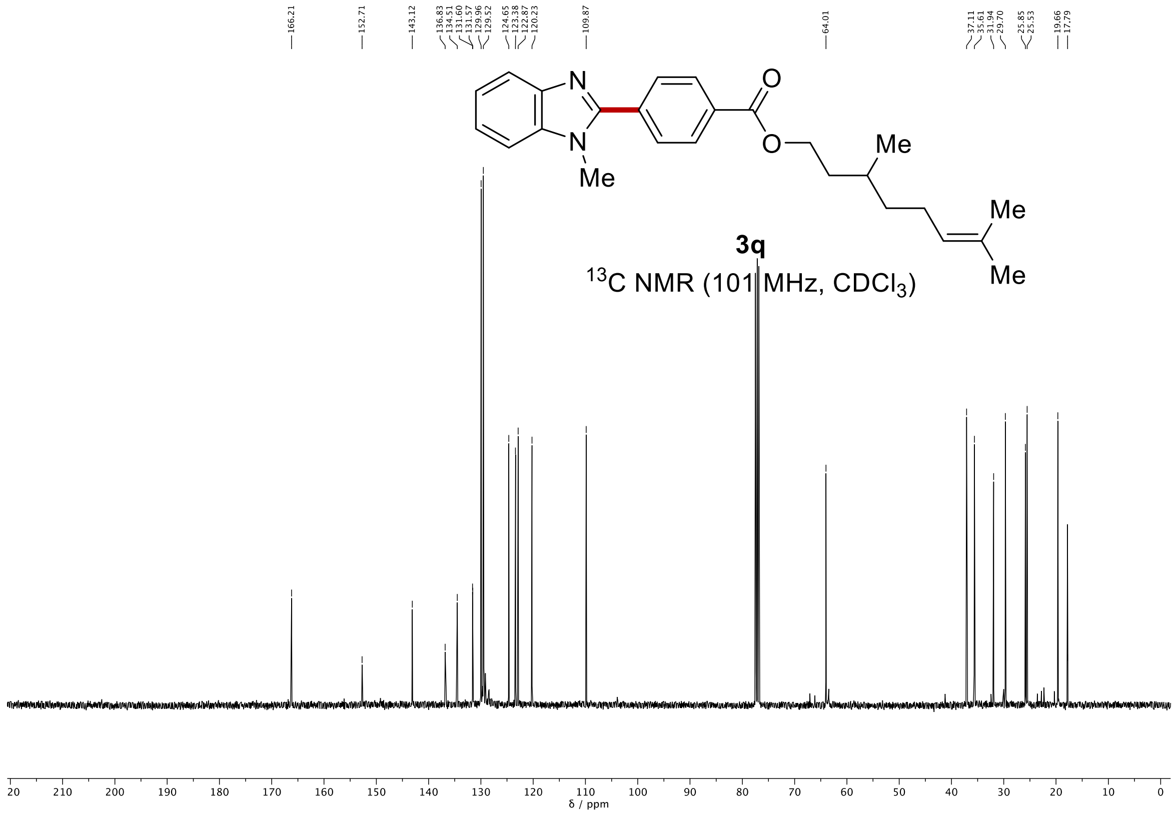
166.21 152.71 143.12 136.83 134.51 131.60 131.57 129.96 129.52 124.65 123.38 122.87 120.23 109.87 64.01 37.11 35.61 31.94 29.70 25.85 25.53 19.66 17.79
3q
13C NMR (101 MHz, CDCl3)
δ / ppm

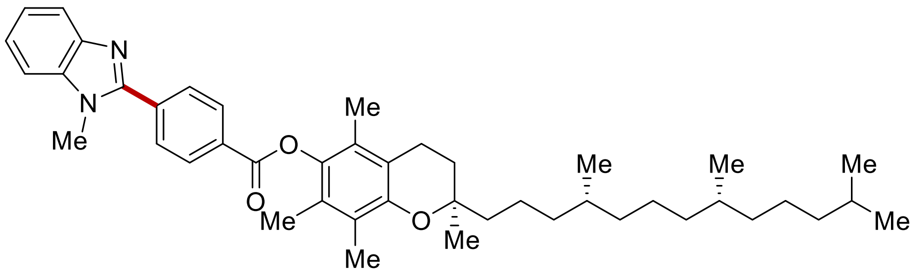

3r

^{1}H NMR (500 MHz, $CDCl_3$)

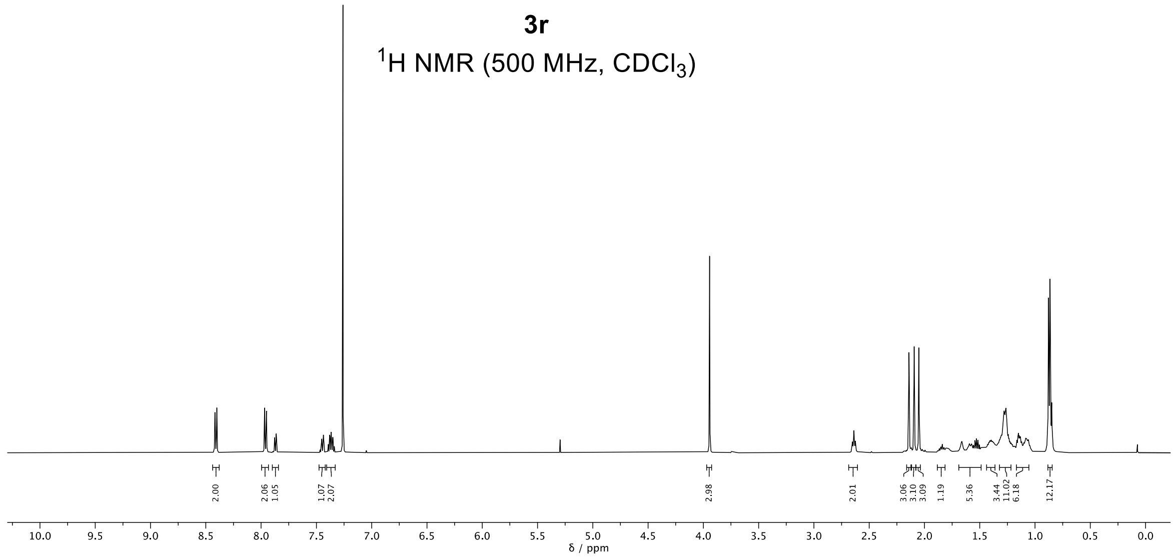

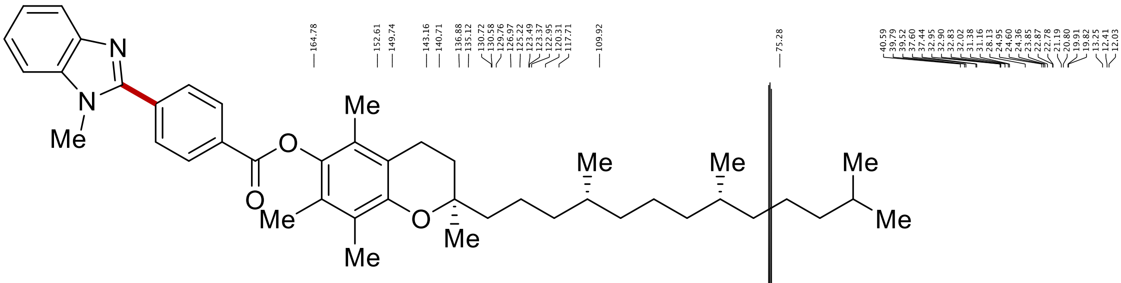

3r

^{13}C NMR (126 MHz, $CDCl_3$)

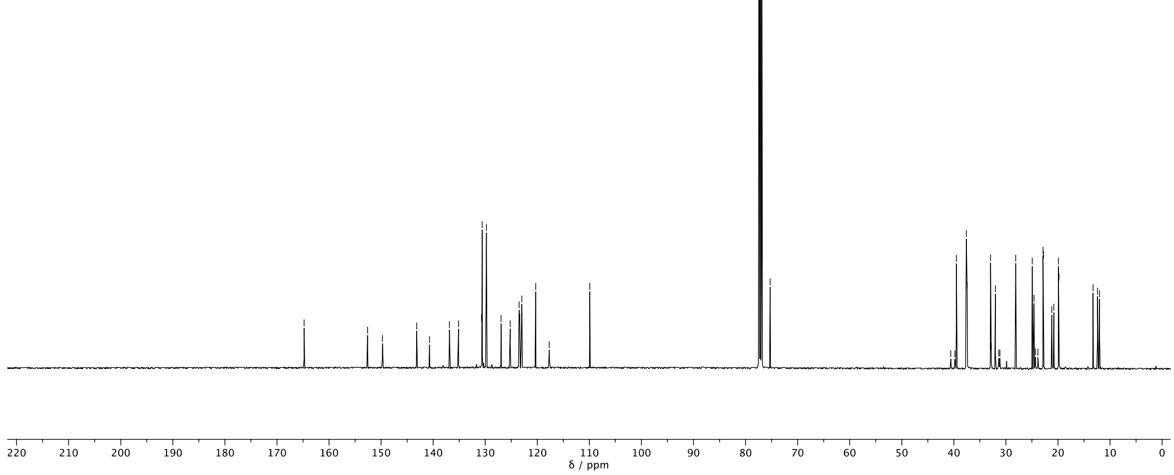

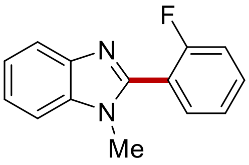

3s

^{1}H NMR (400 MHz, $CDCl_3$)

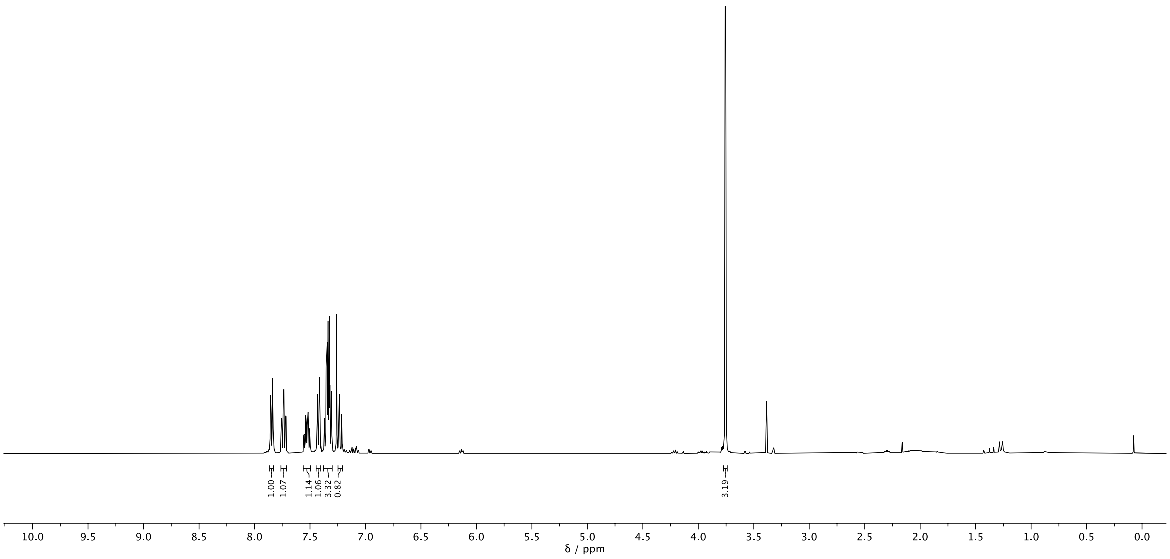

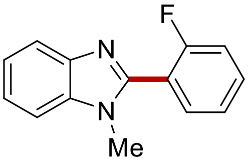

3s

^{13}C NMR (101 MHz, $CDCl_3$)

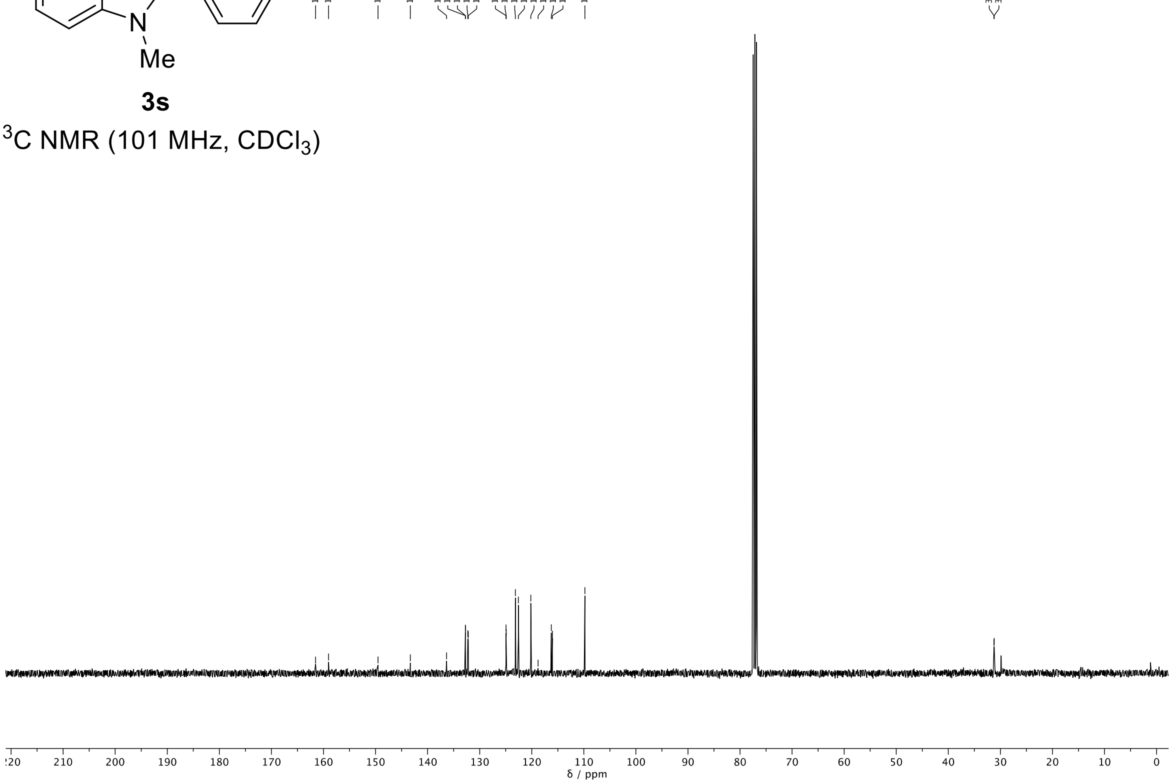

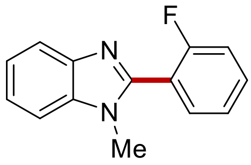

3s

^{19}F NMR (377 MHz, $CDCl_3$)

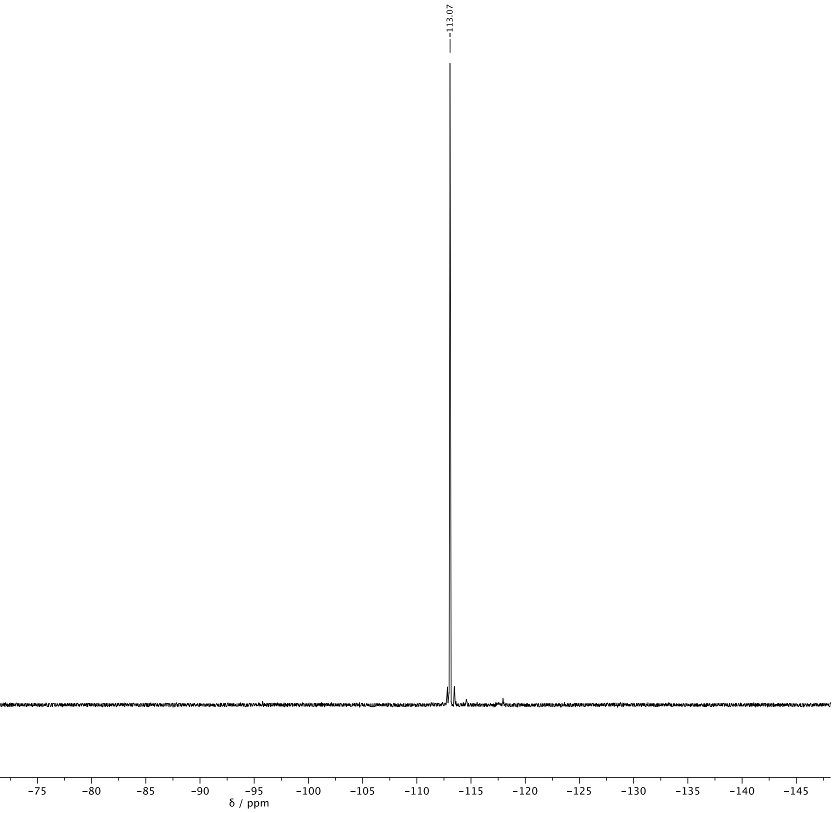

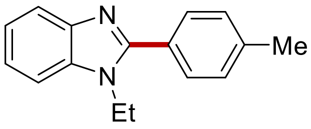

5a

^{1}H NMR (400 MHz, $CDCl_3$)

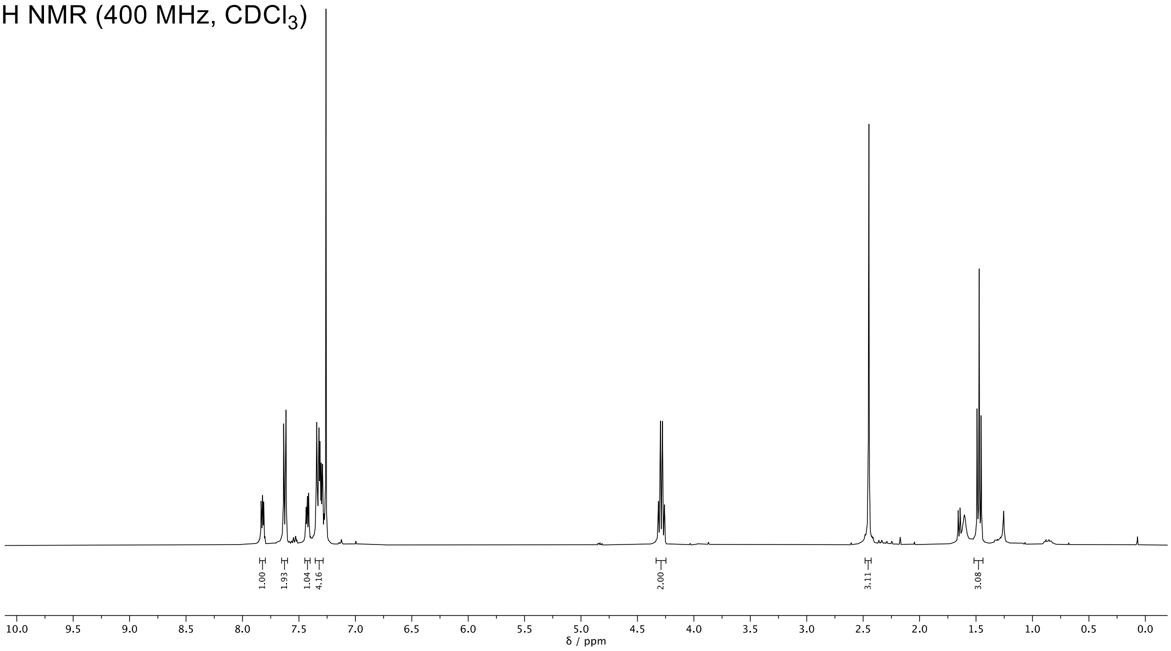

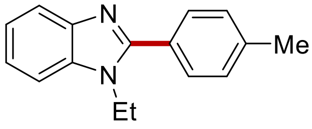

5a

^{13}C NMR (101 MHz, $CDCl_3$)

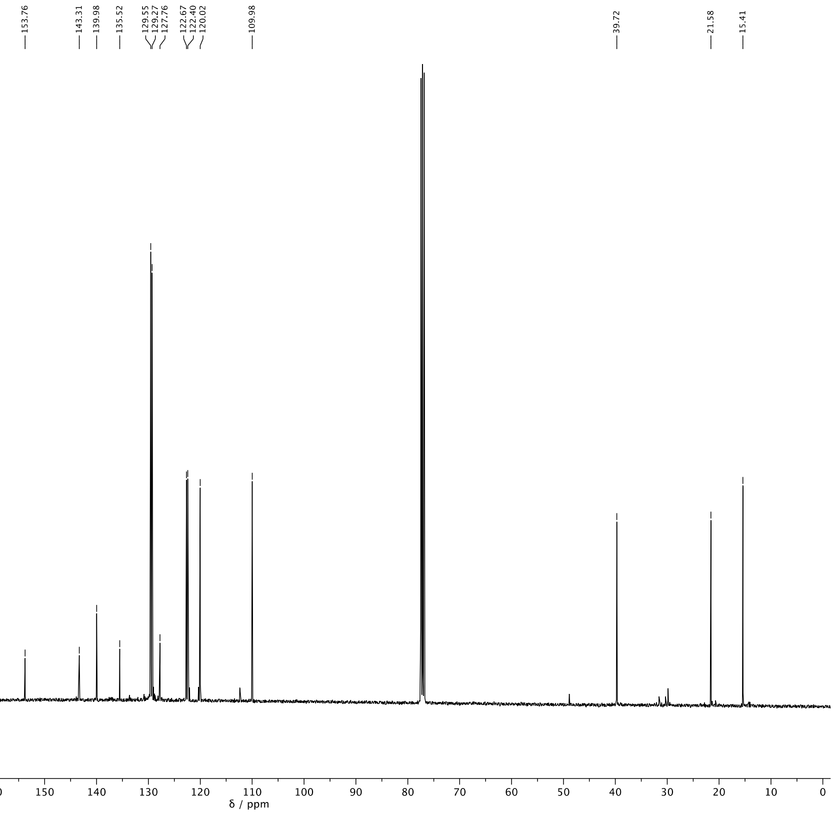

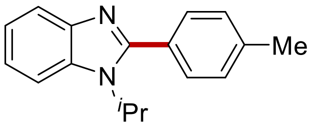

5b

^{1}H NMR (400 MHz, $CDCl_3$)

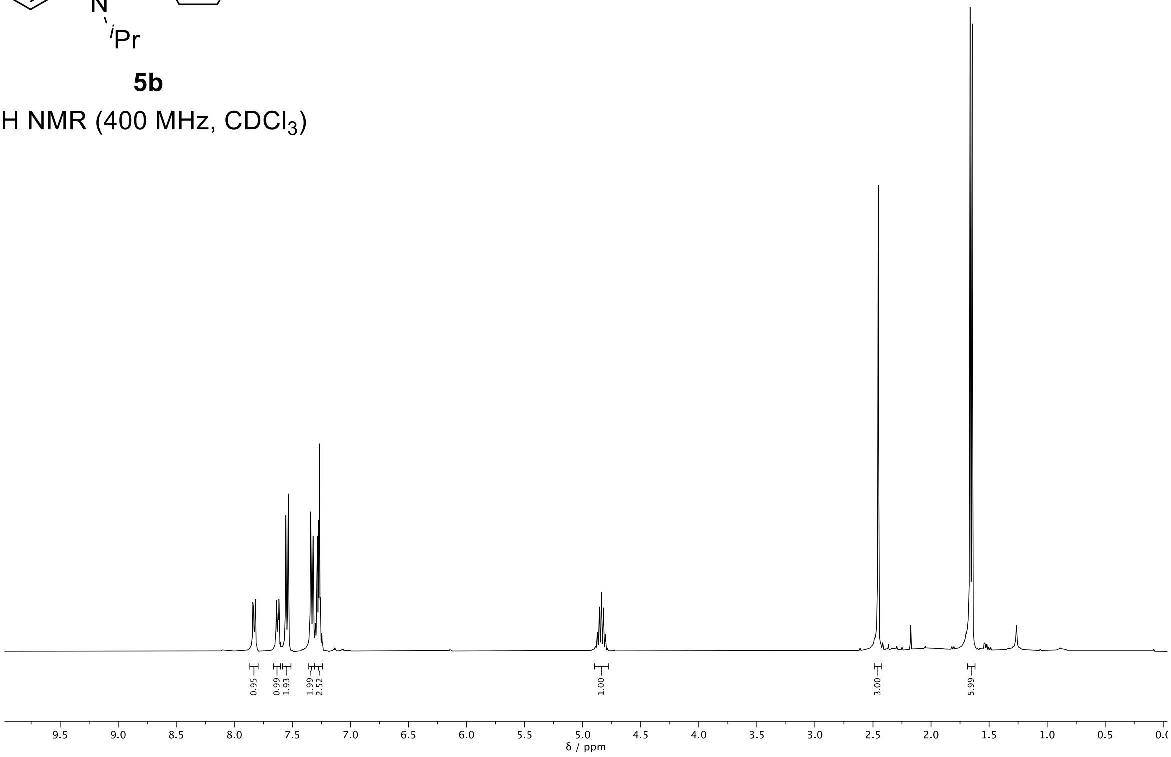

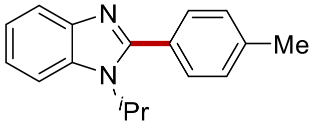

5b

^{13}C NMR (101 MHz, $CDCl_3$)

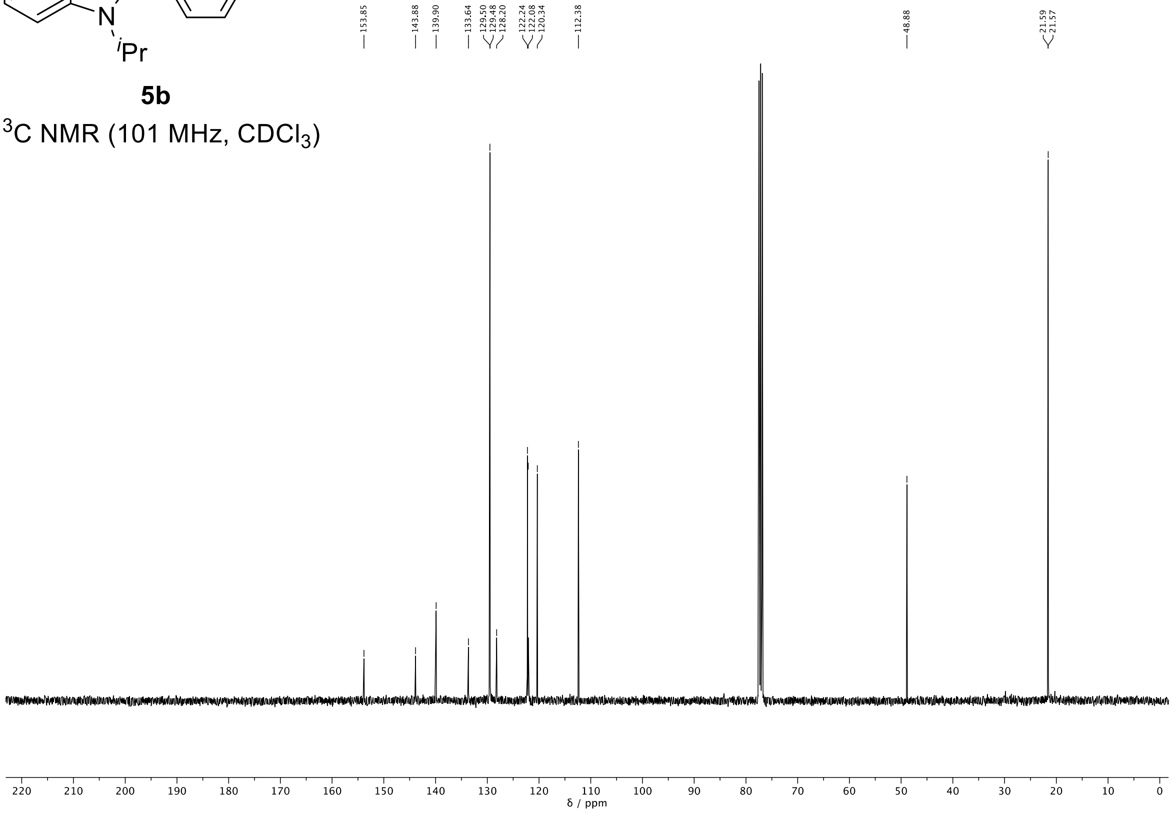

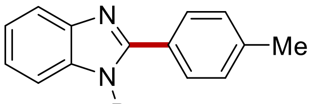

5c

^{1}H NMR (400 MHz, $CDCl_3$)

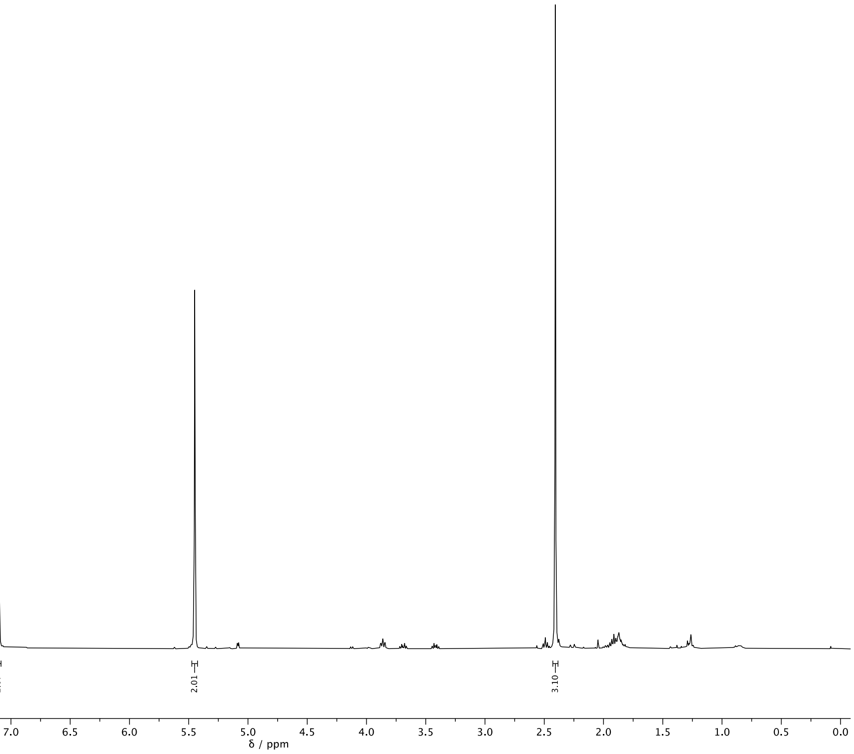

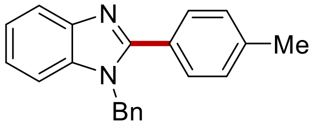

5c

^{13}C NMR (101 MHz, $CDCl_3$)

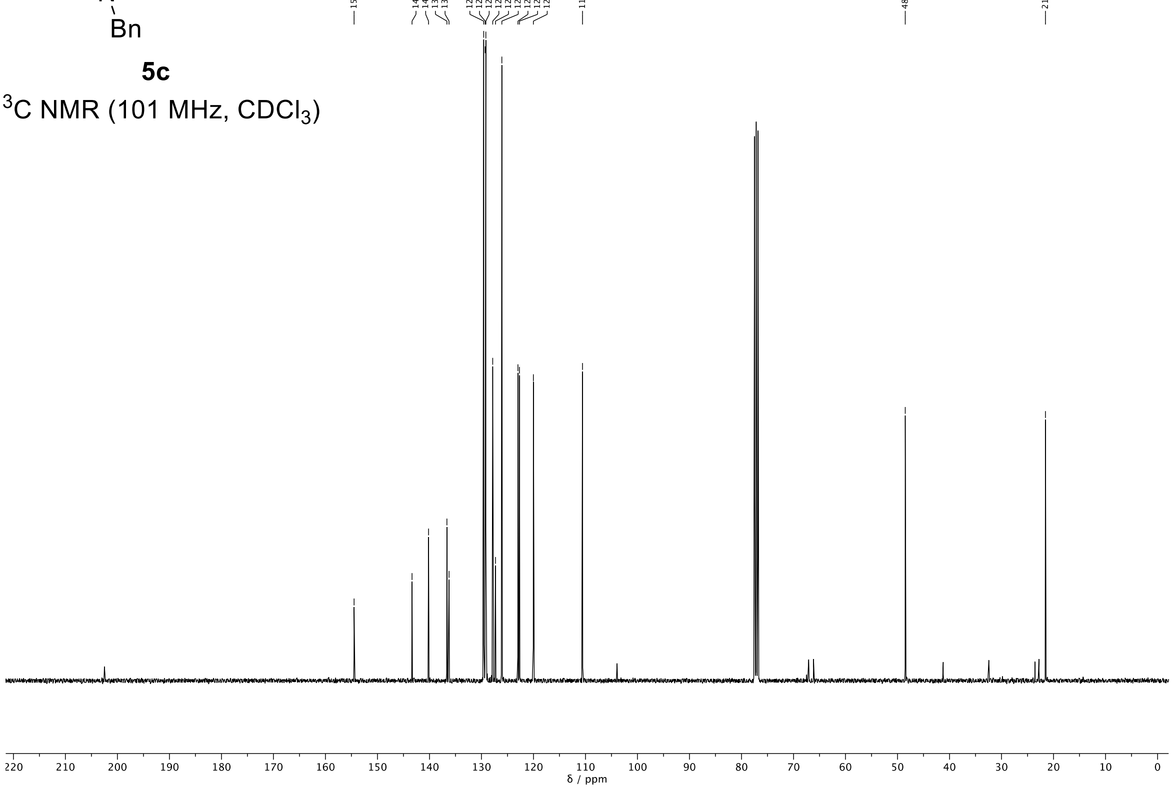

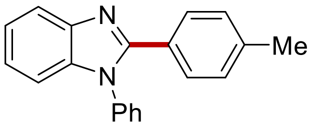

5d

^{1}H NMR (400 MHz, $CDCl_3$)

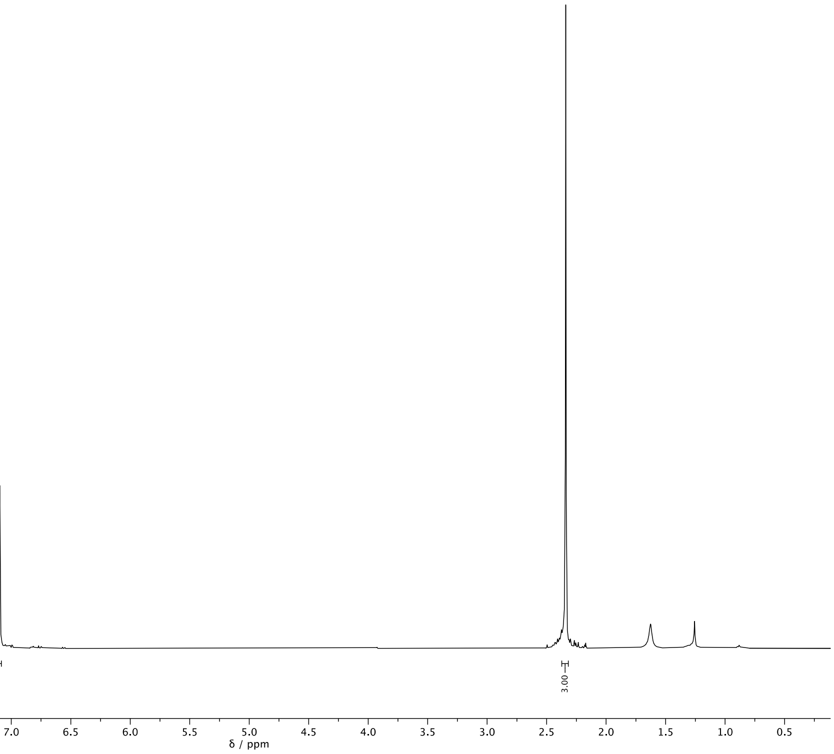

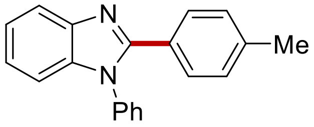

5d

^{13}C NMR (101 MHz, $CDCl_3$)

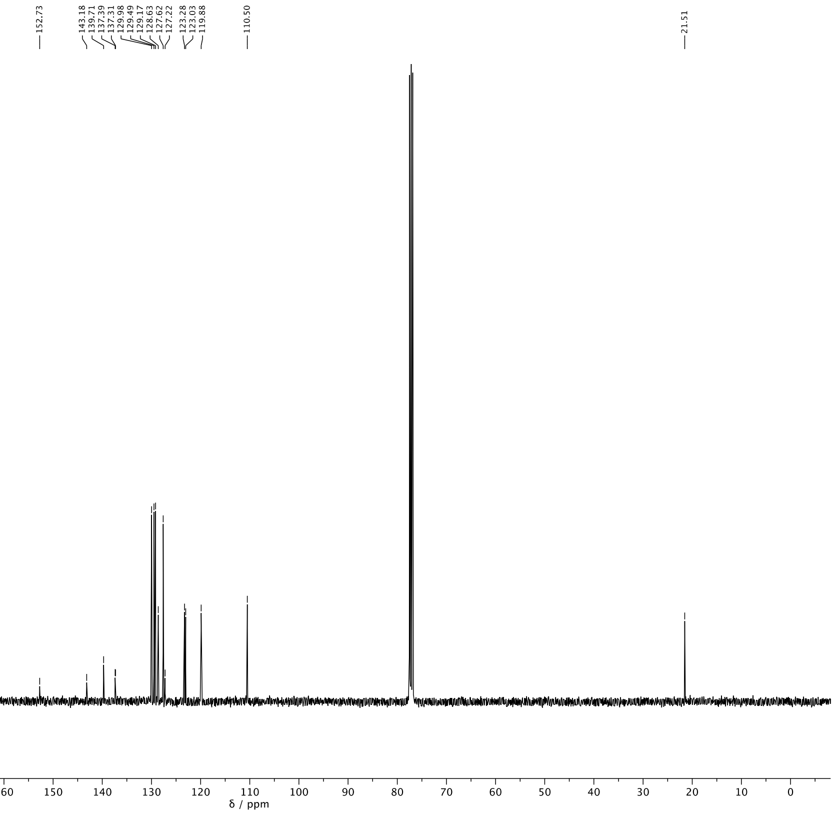

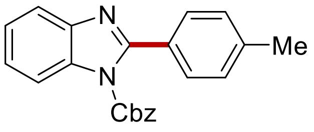

5e

^{1}H NMR (400 MHz, $CDCl_3$)

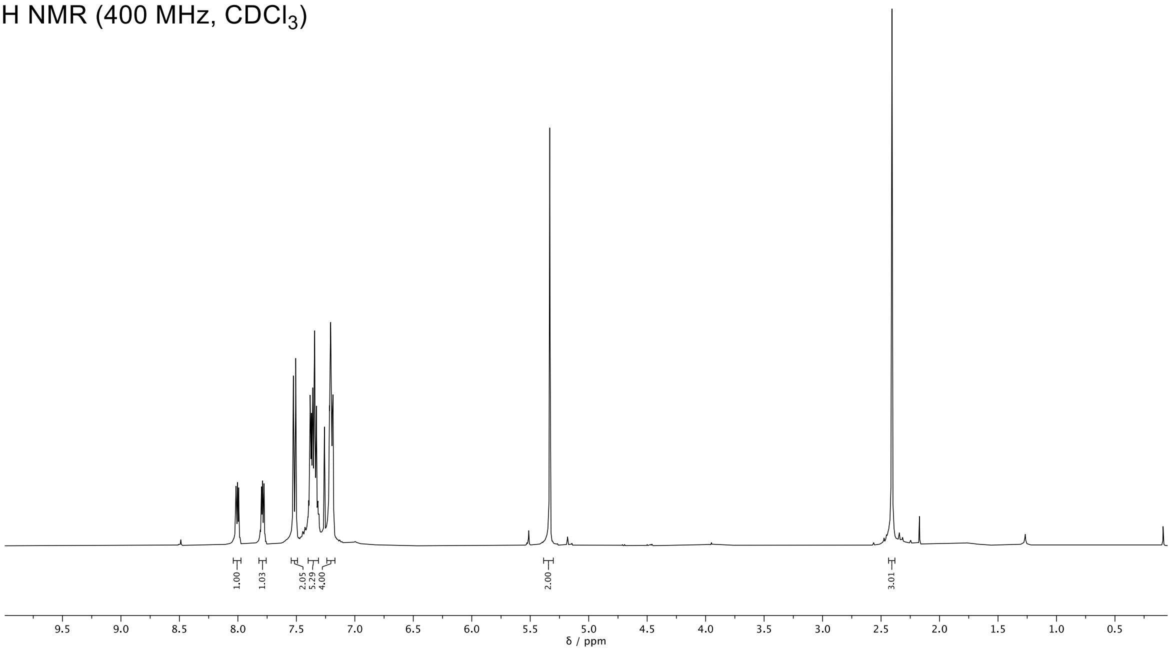

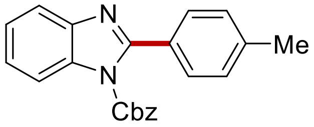

5e

^{13}C NMR (101 MHz, $CDCl_3$)

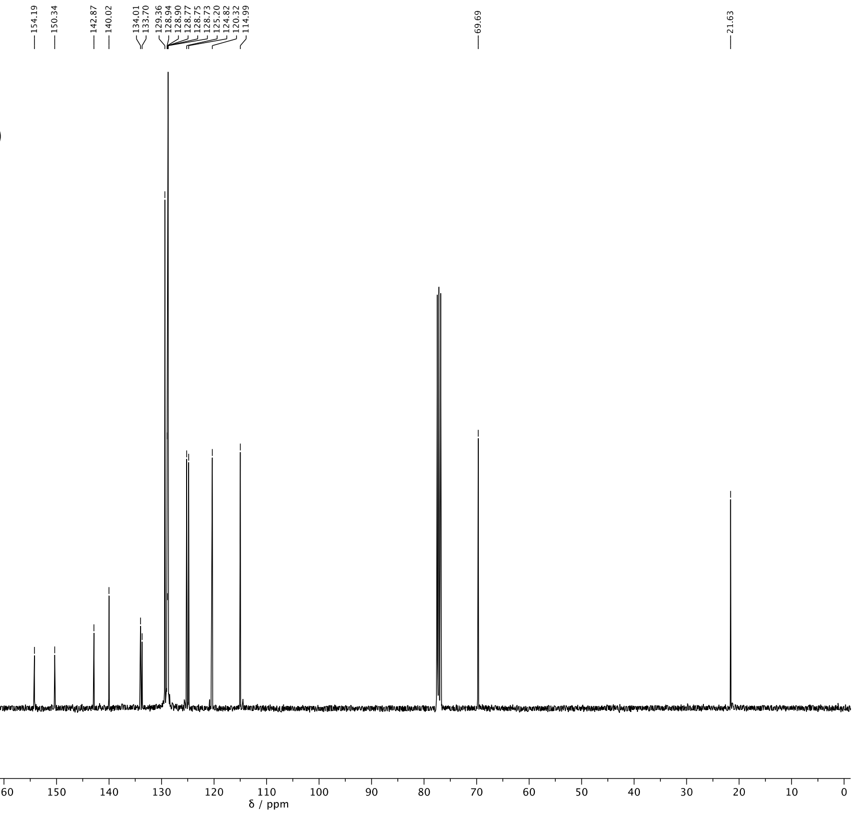

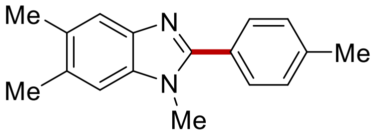

5f

^{1}H NMR (400 MHz, $CDCl_3$)

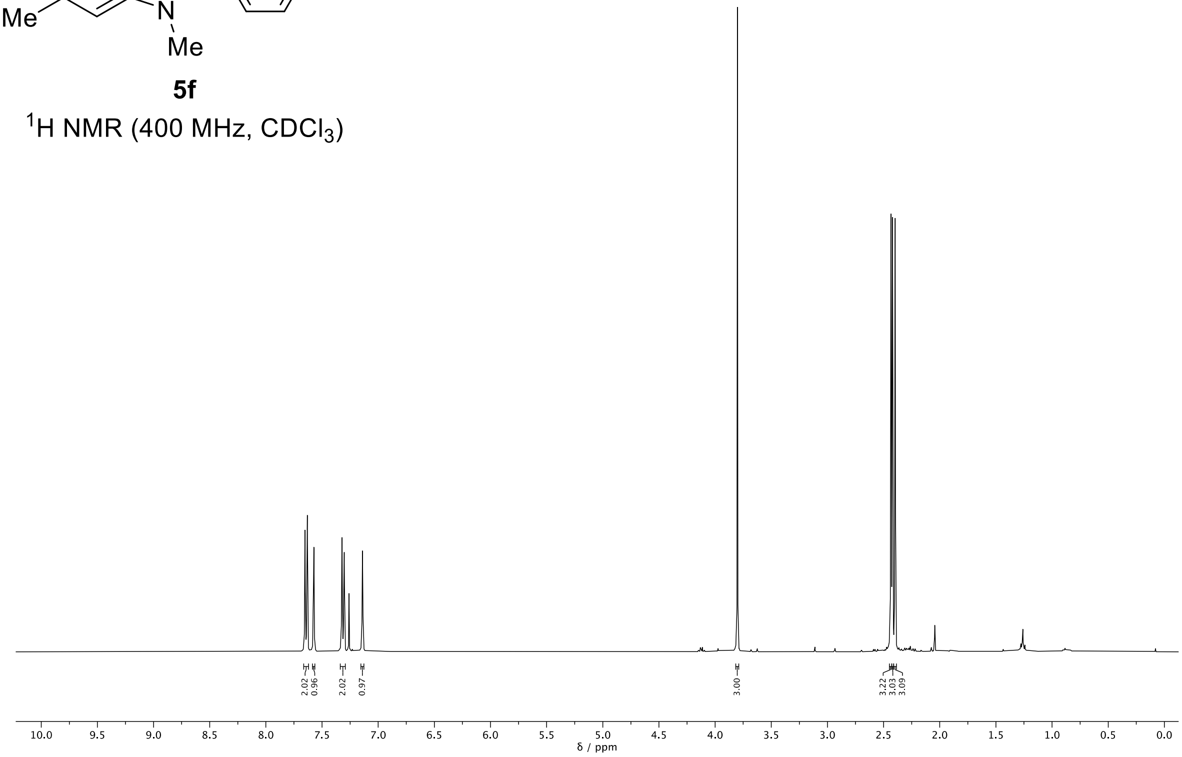

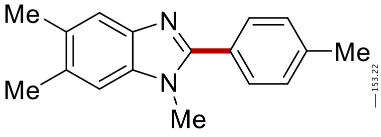

5f

^{13}C NMR (101 MHz, $CDCl_3$)

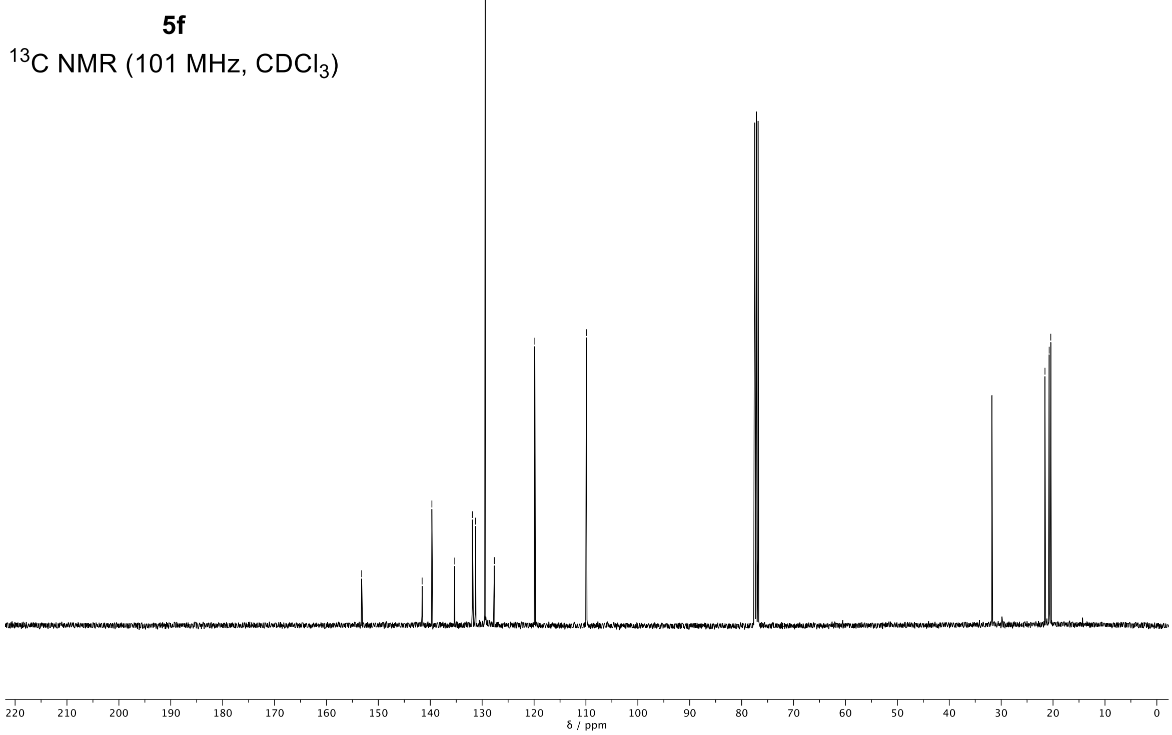

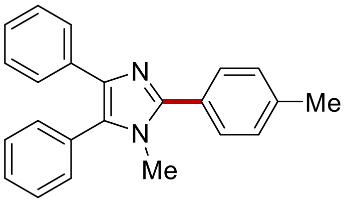

5g

^{1}H NMR (400 MHz, $CDCl_3$)

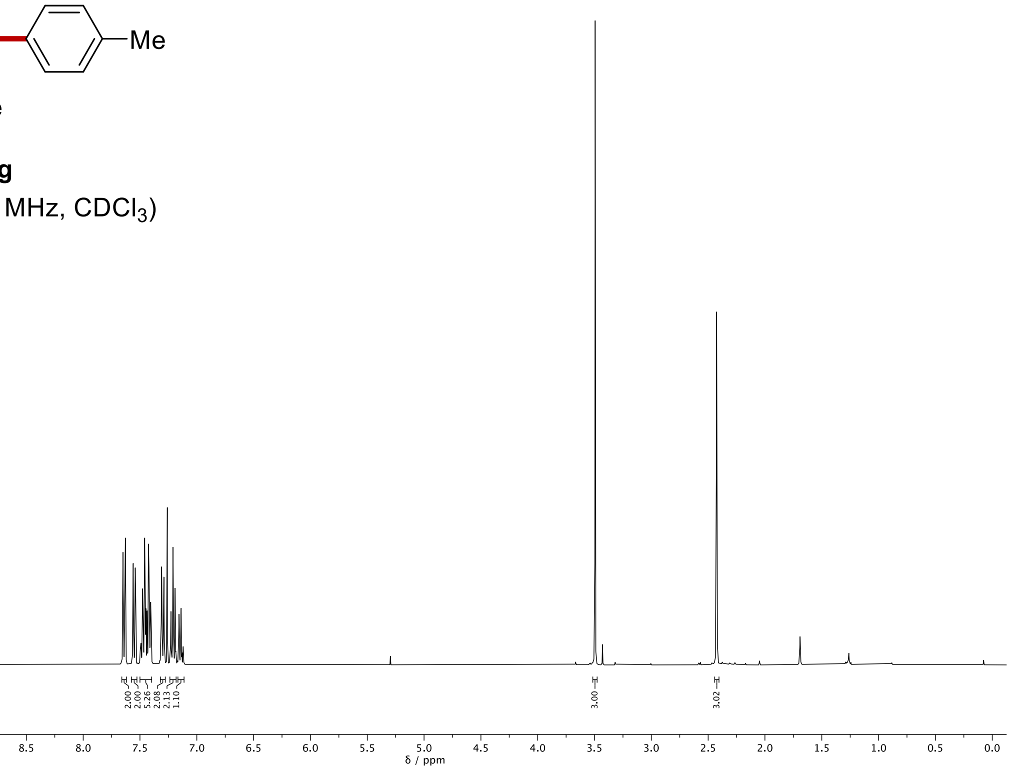

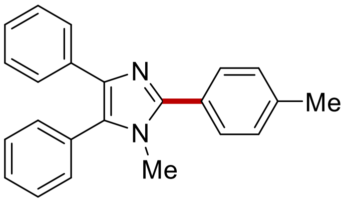

5g

^{13}C NMR (101 MHz, $CDCl_3$)

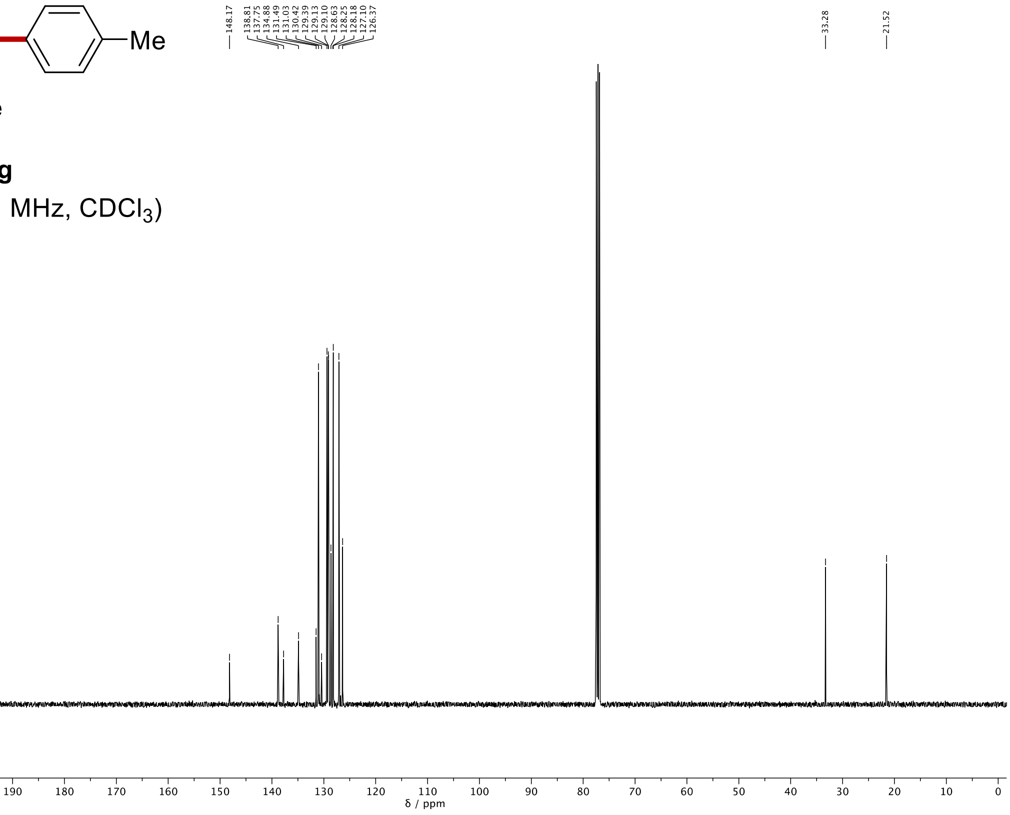

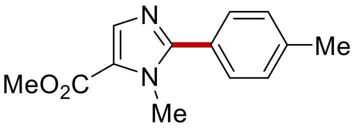

5h

^{1}H NMR (400 MHz, $CDCl_3$)

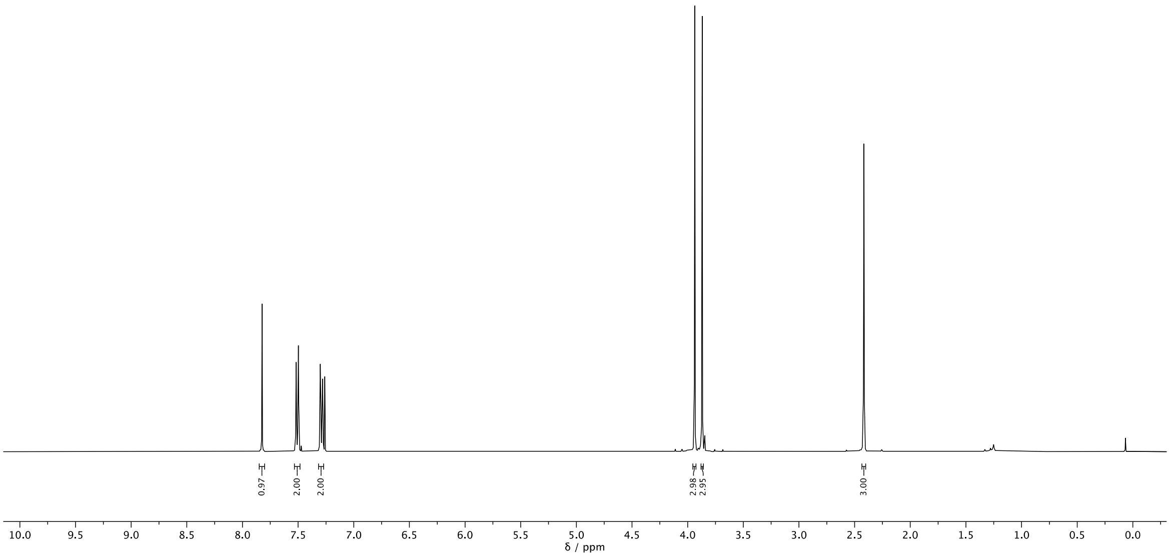

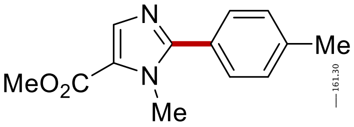

5h

^{13}C NMR (101 MHz, $CDCl_3$)

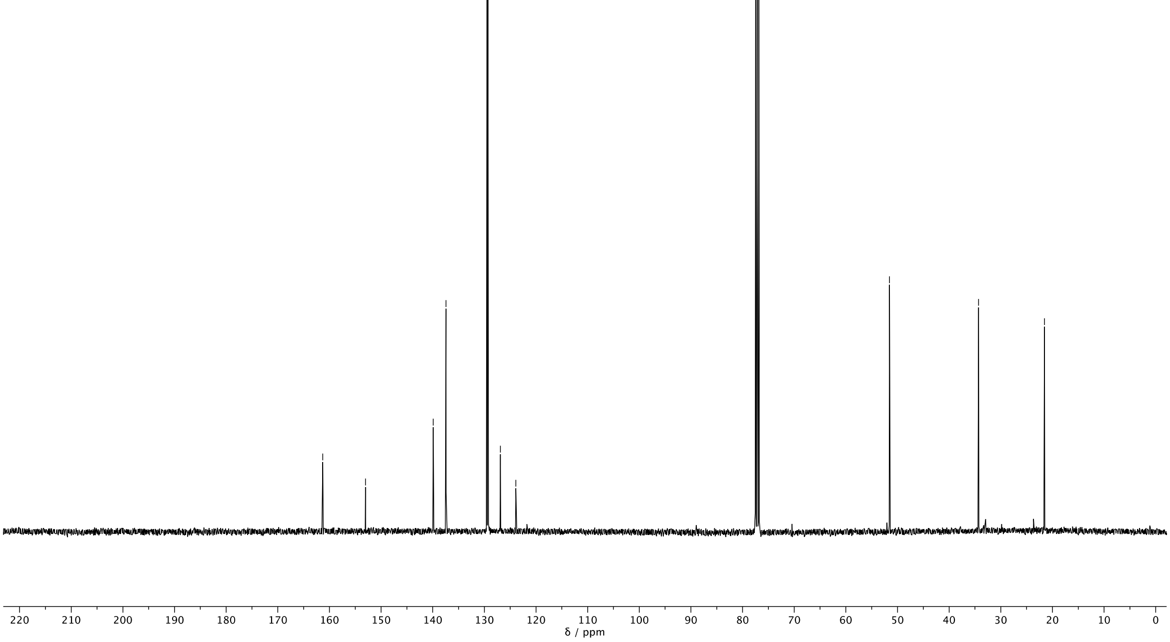

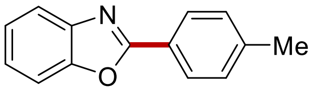

5i

^{1}H NMR (400 MHz, $CDCl_3$)

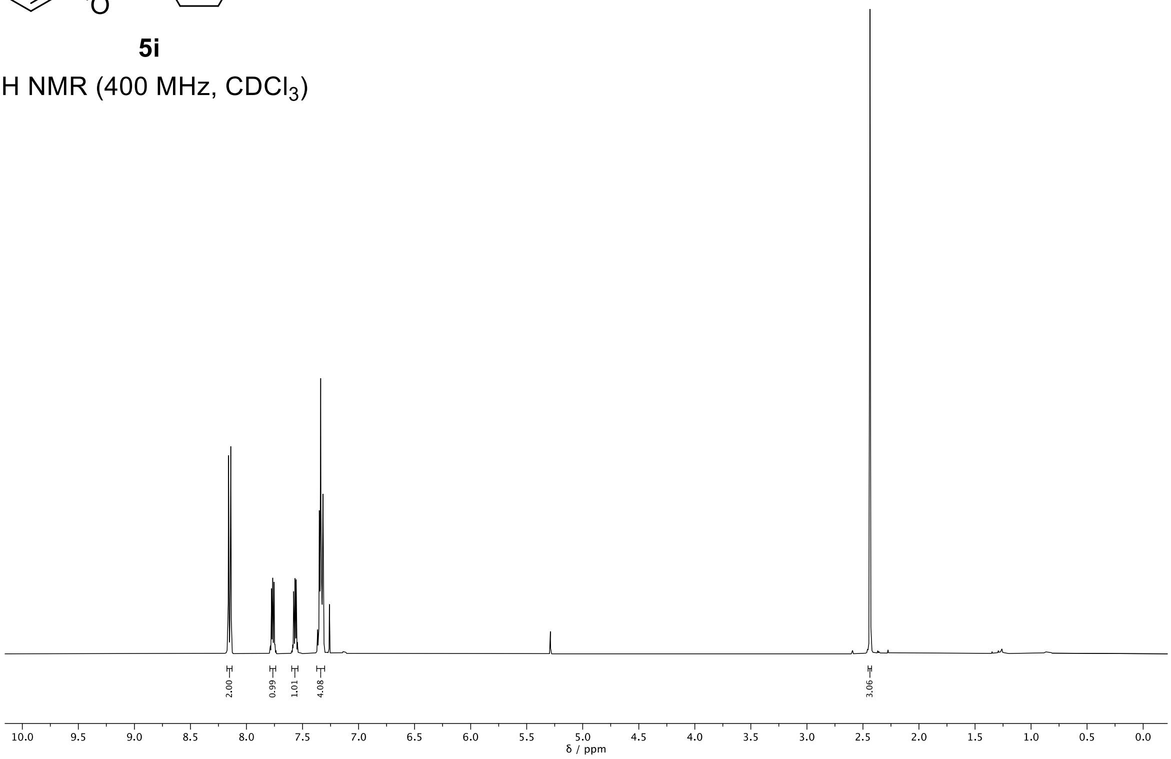

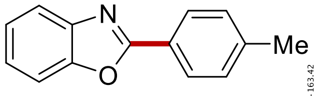

5i

^{13}C NMR (101 MHz, $CDCl_3$)

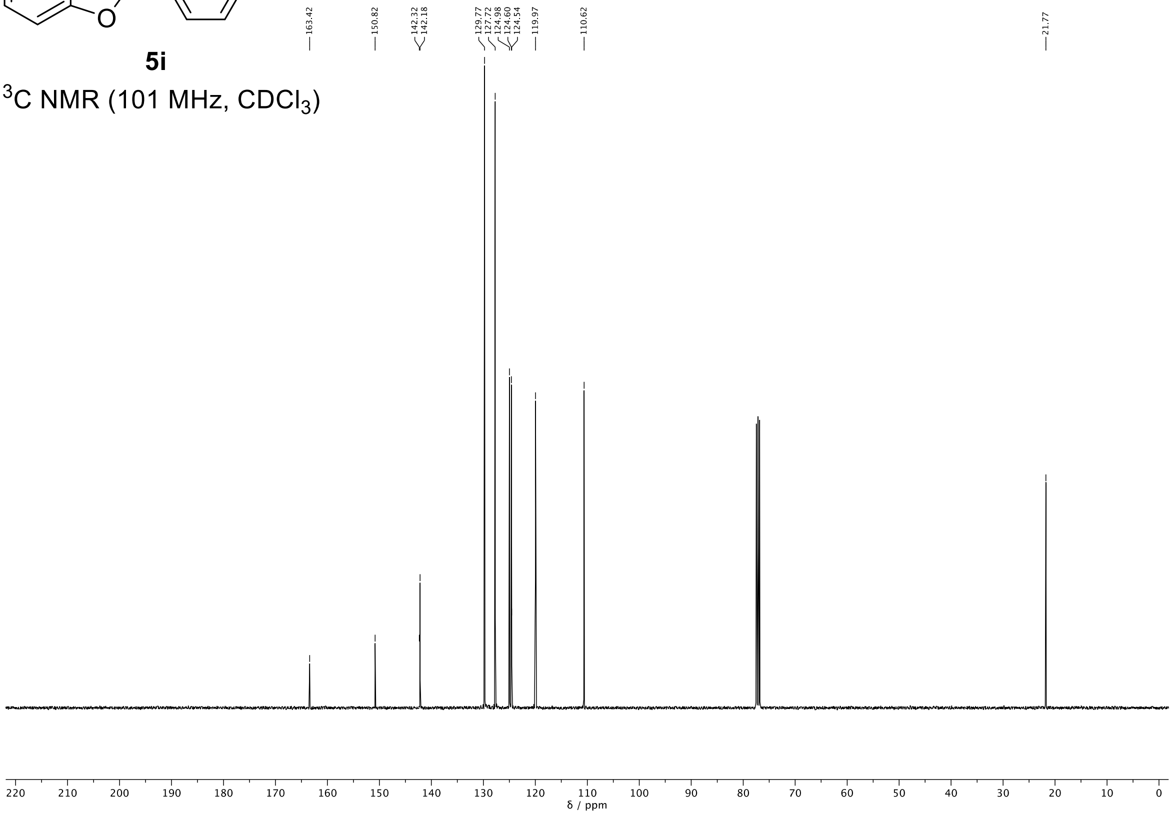

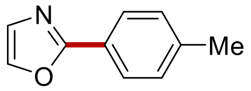

5j

^{1}H NMR (400 MHz, $CDCl_3$)

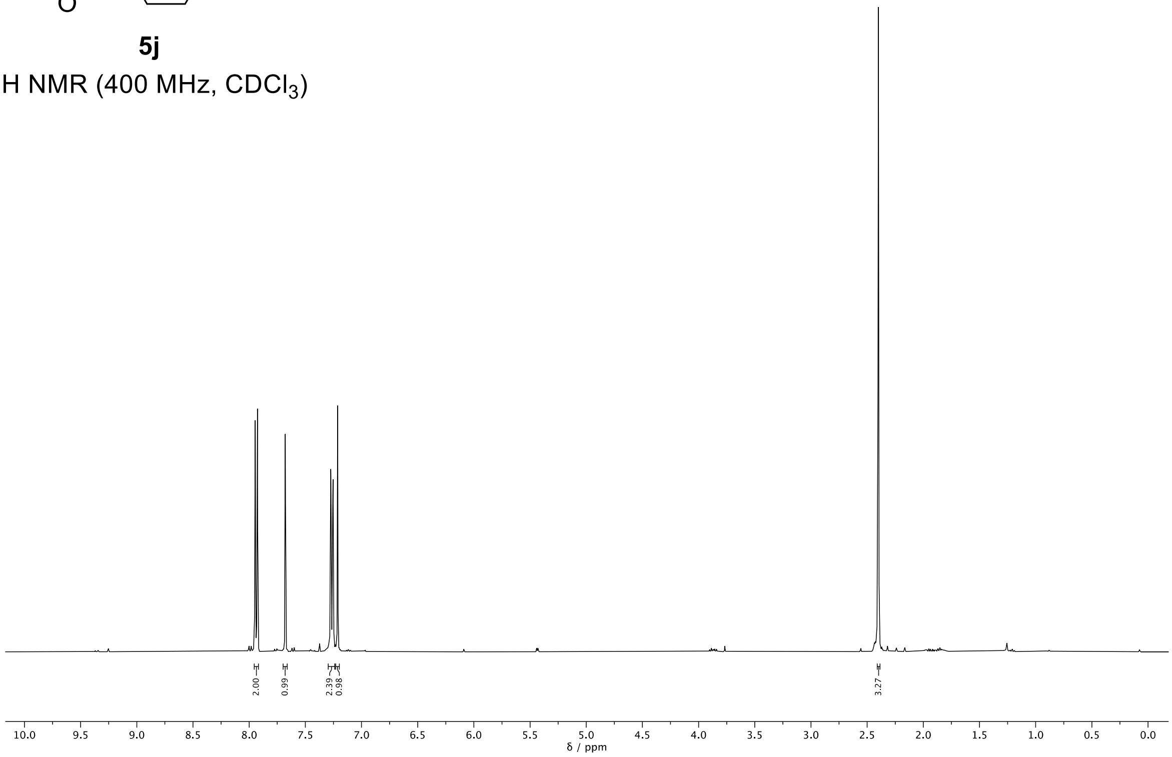

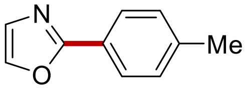

5j

^{13}C NMR (101 MHz, $CDCl_3$)

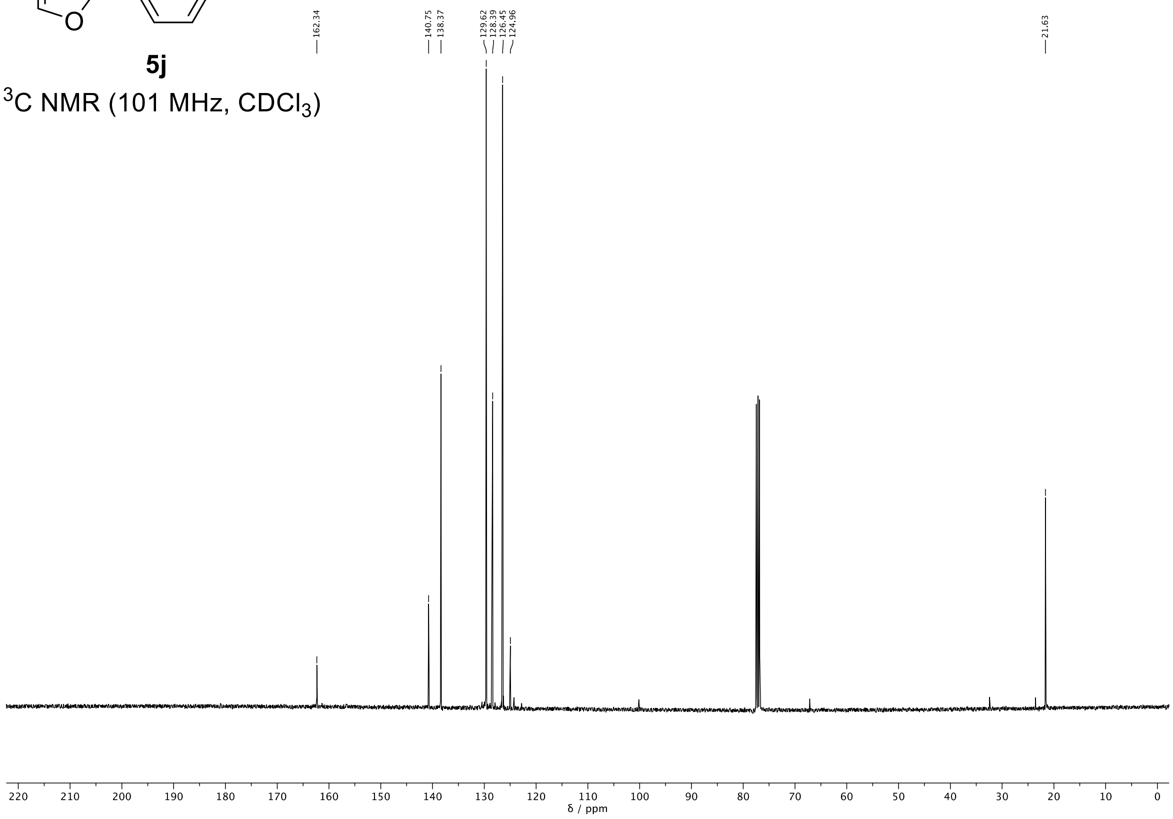

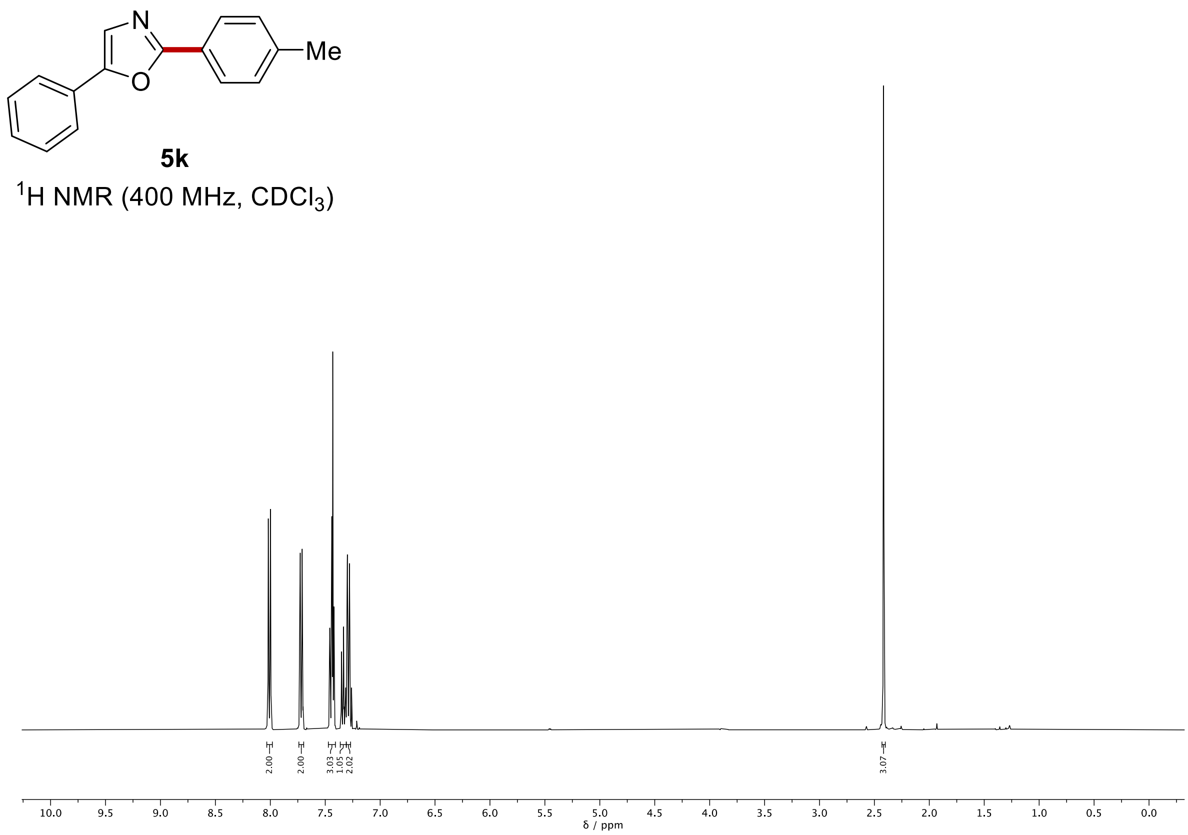
Me
N
O
5k
^{1}H NMR (400 MHz, $CDCl_3$)
δ / ppm

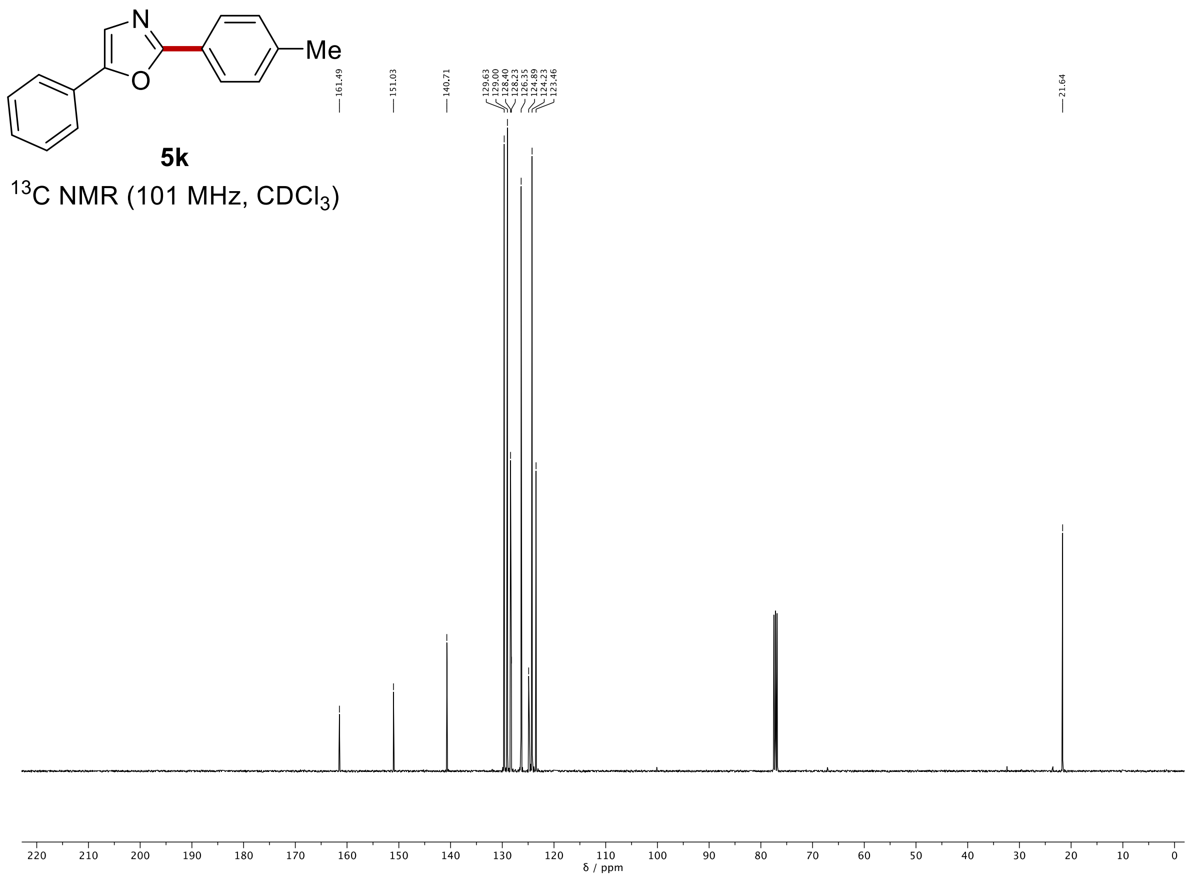
Me
N
O
5k
^{13}C NMR (101 MHz, $CDCl_3$)
δ / ppm

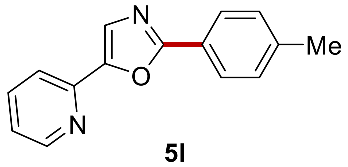

5l

^{1}H NMR (400 MHz, $CDCl_3$)

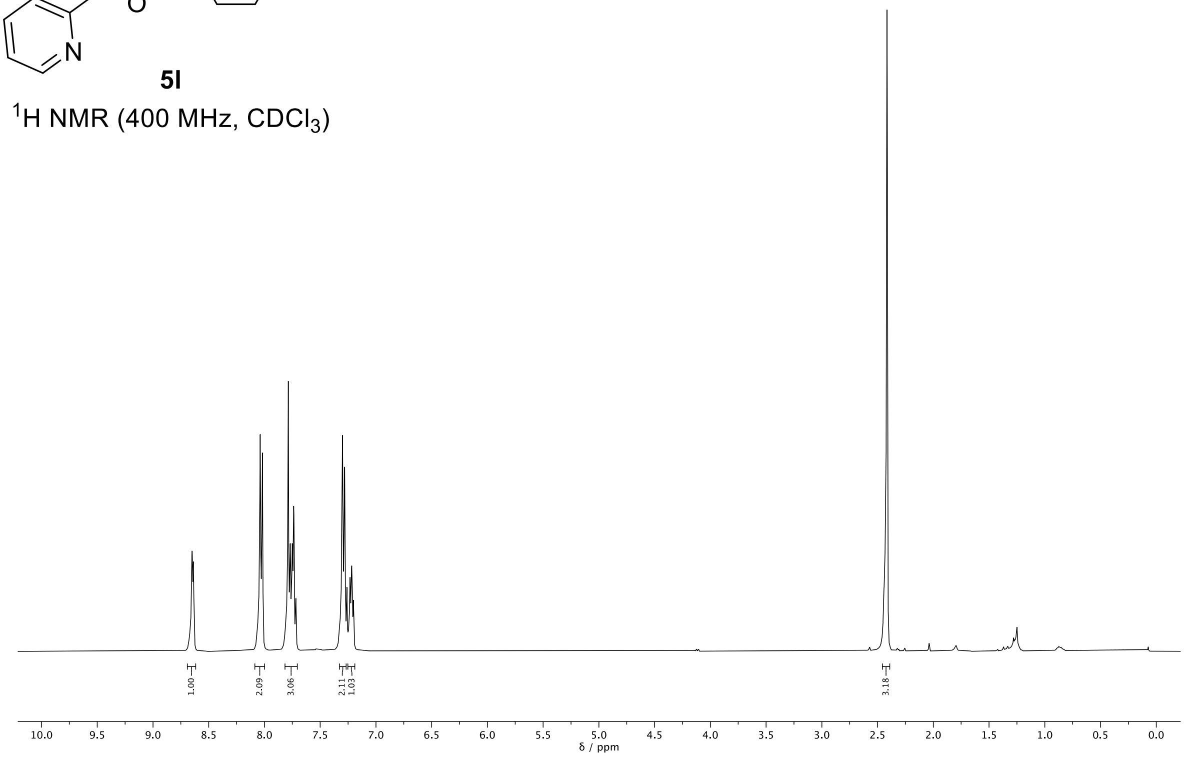

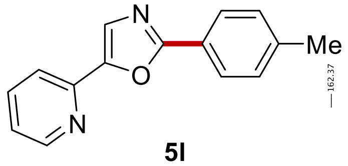

5l

^{13}C NMR (101 MHz, $CDCl_3$)

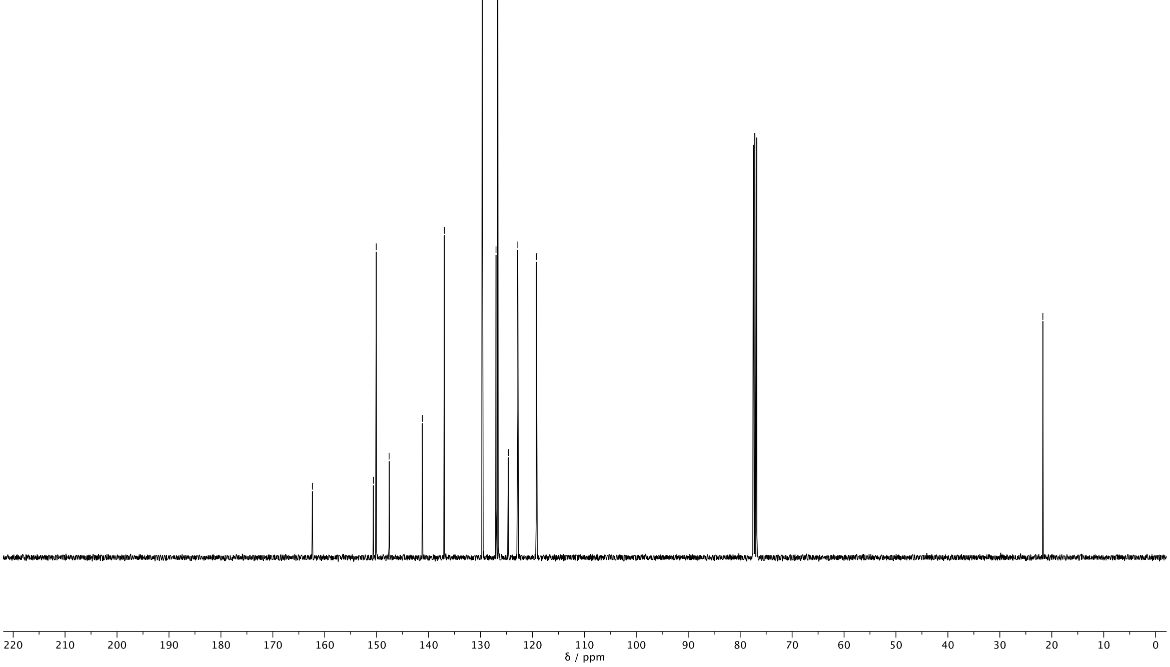

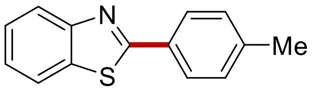

5m

^{1}H NMR (400 MHz, $CDCl_3$)

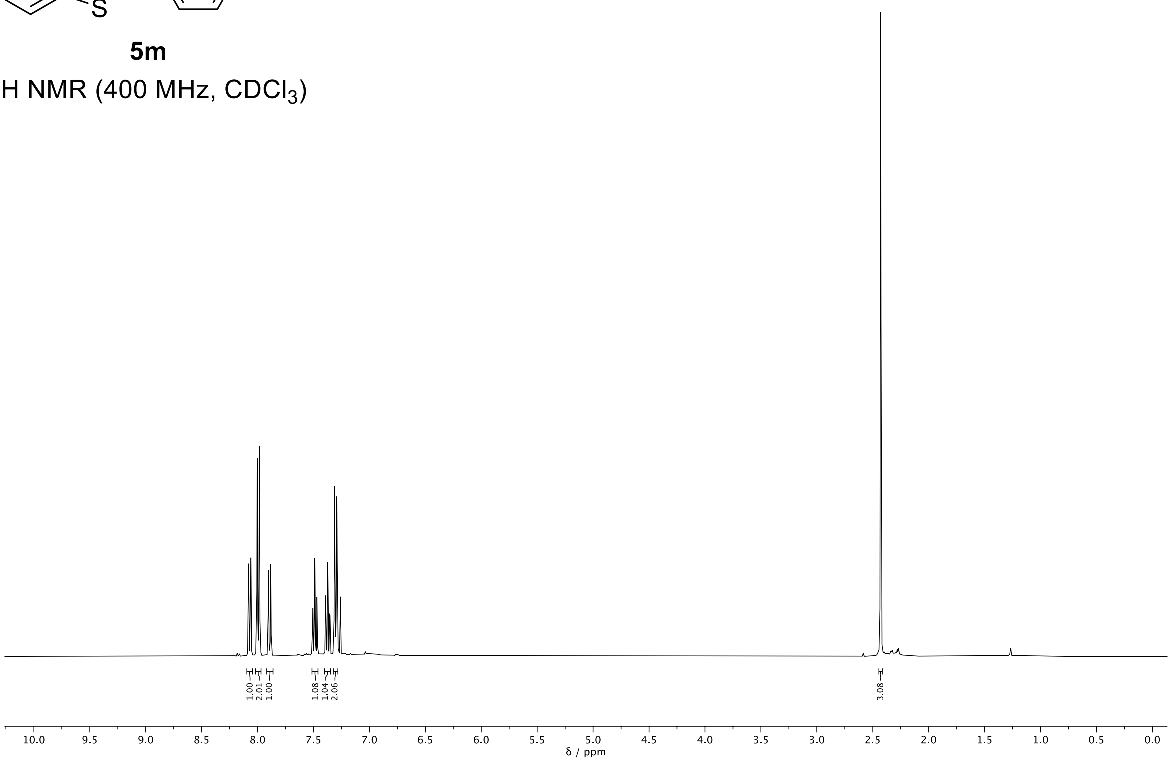

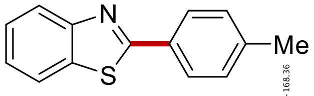

5m

^{13}C NMR (101 MHz, $CDCl_3$)

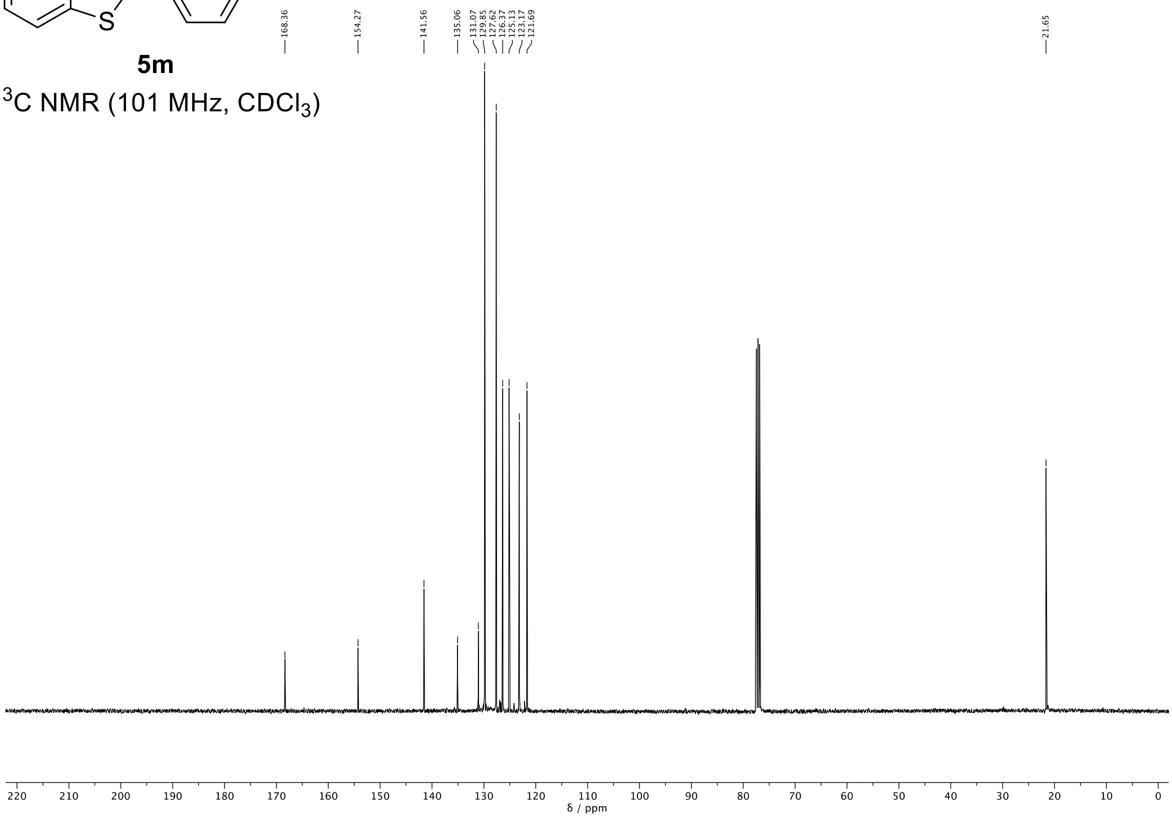

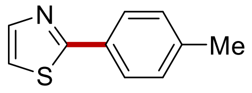

5n

^{1}H NMR (400 MHz, $CDCl_3$)

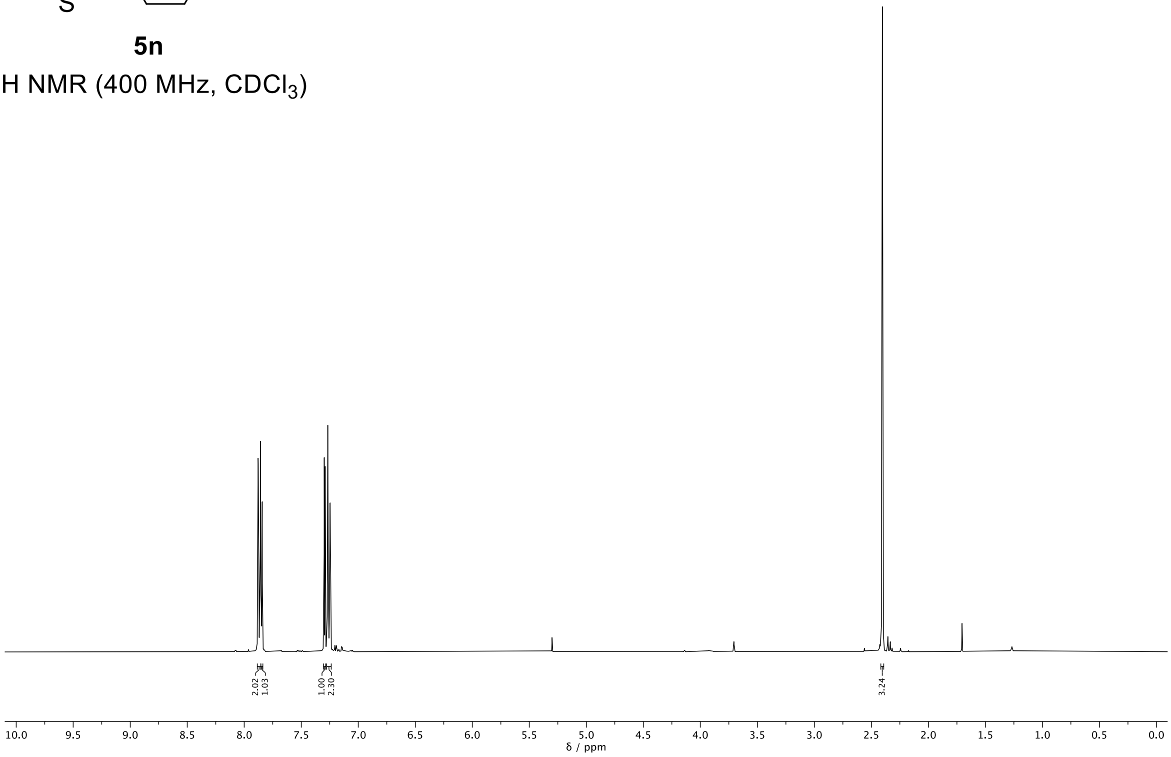

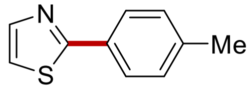

5n

^{13}C NMR (101 MHz, $CDCl_3$)

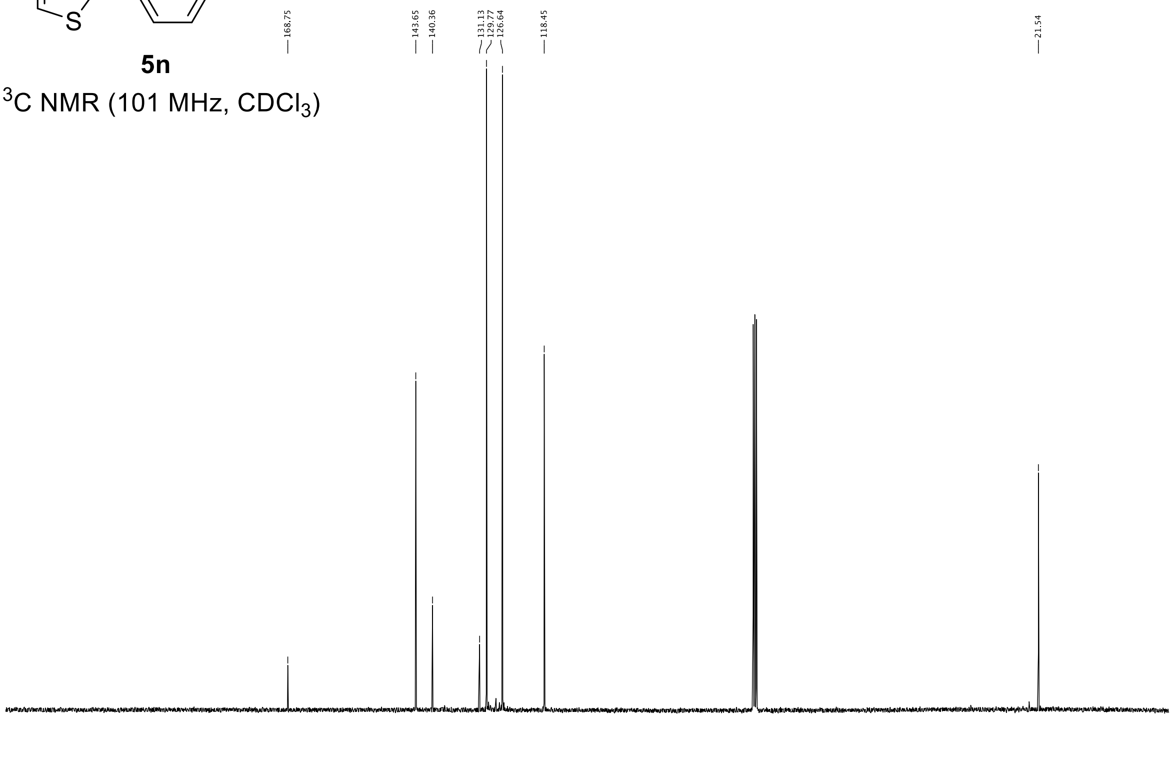

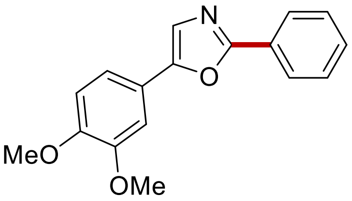

5o

^{1}H NMR (400 MHz, $CDCl_3$)

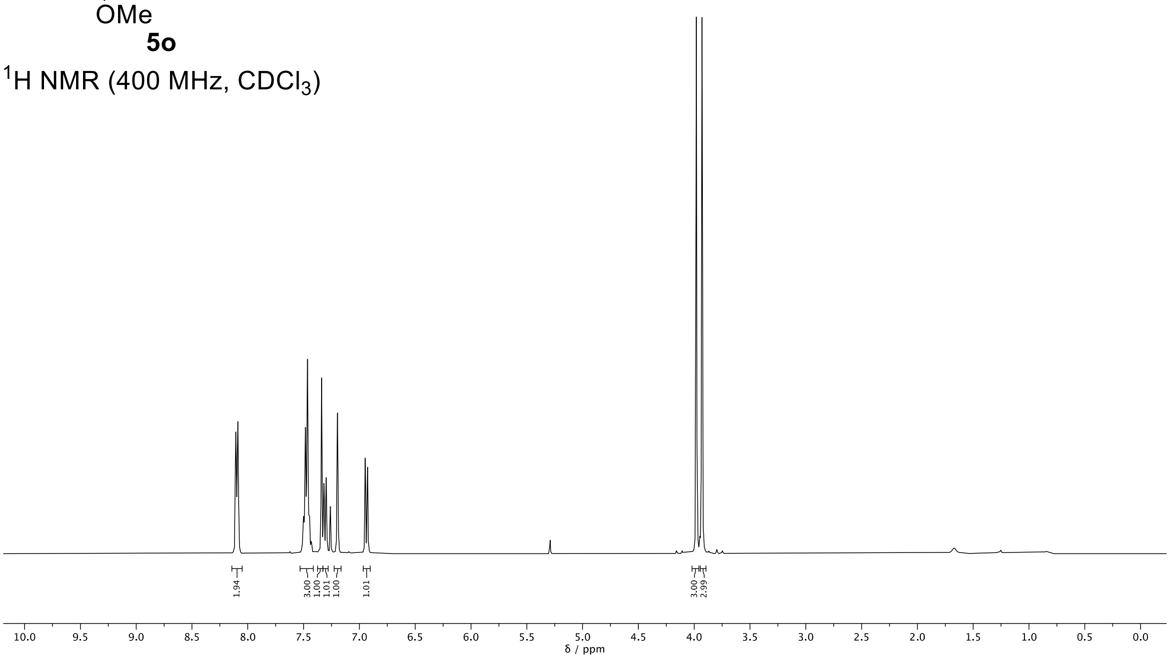

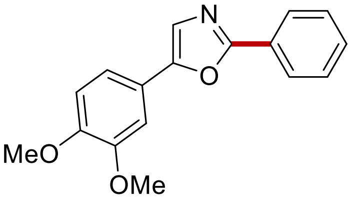

5o

^{13}C NMR (101 MHz, $CDCl_3$)

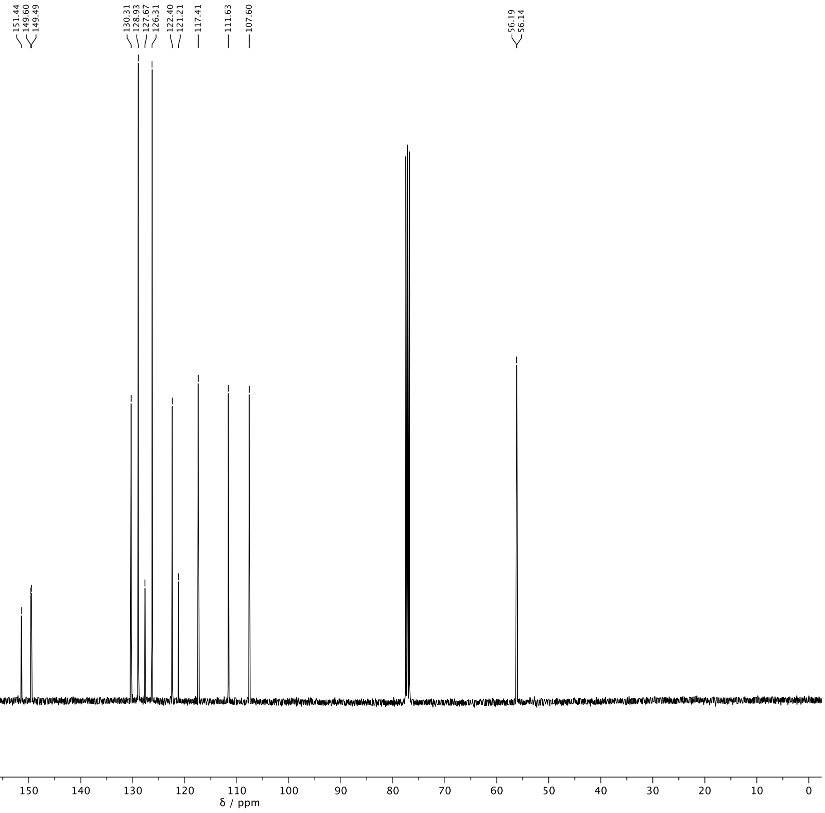

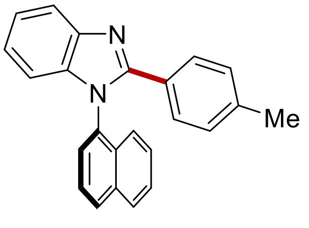

7

^{1}H NMR (400 MHz, $CDCl_3$)

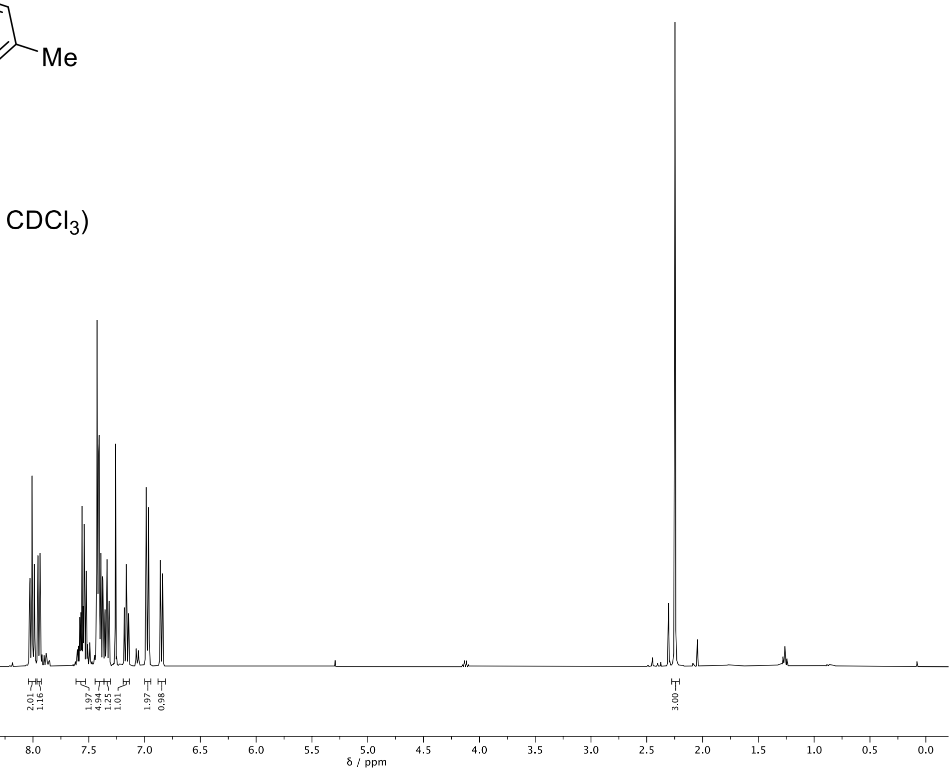

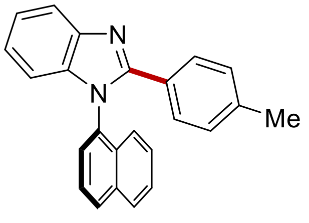

7

^{13}C NMR (101 MHz, $CDCl_3$)

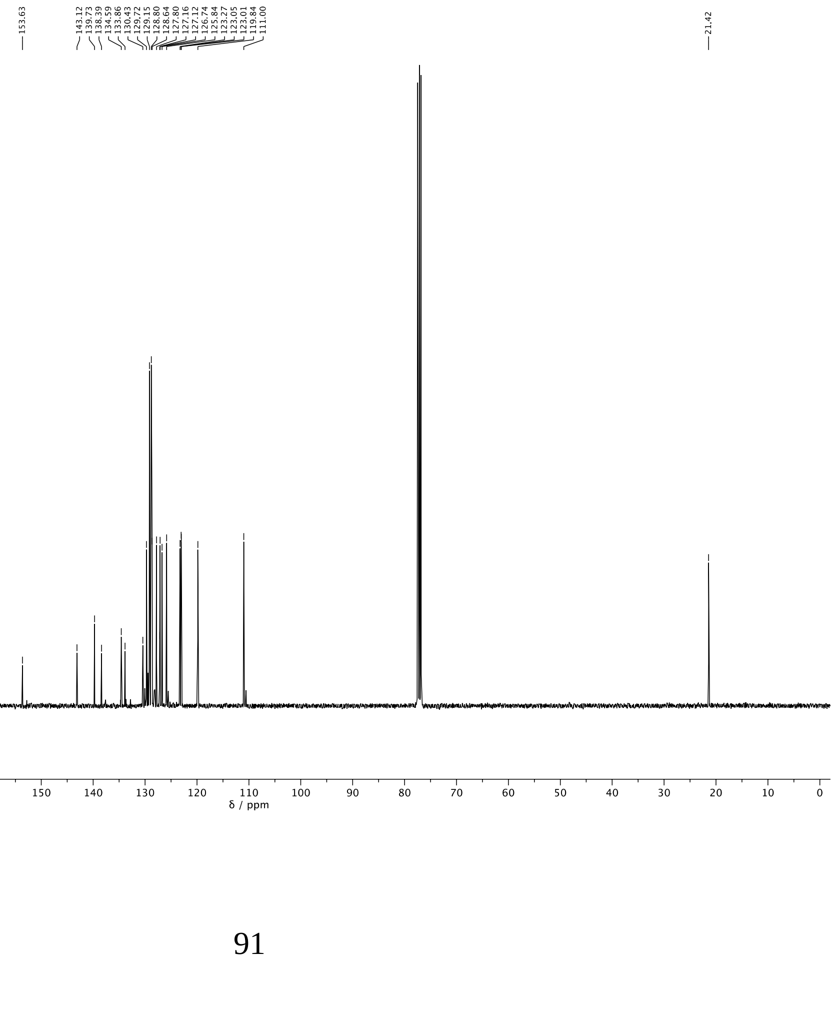

Supplement: SC-015-D4SC00393D-s001 [file SC-015-D4SC00393D-s001.pdf]
